# Long-term data on the proposed adalimumab biosimilar BCD-057 in patients with moderate to severe psoriasis: A randomized controlled trial

**DOI:** 10.1371/journal.pone.0263214

**Published:** 2022-02-07

**Authors:** Alexey V. Samtsov, Andrey L. Bakulev, Vladislav R. Khairutdinov, Muza M. Kokhan, Tat’yana V. Korotaeva, Iskander K. Minullin, Olga A. Vylegzhanina, Valery V. Dubenskiy, Bulat V. Khalilov, Alkes A. Khotko, Olga S. Zykova, Irina V. Chumachenko, Alexander M. Lukyanov, Antonina V. Artemeva, Polina P. Pukhtinskaia

**Affiliations:** 1 Department of Dermatology, S.M. Kirov Military Medical Academy, Saint-Petersburg, Russia; 2 Department of Dermatology, V.I. Razumovskiy Saratov State Medical University, Saratov, Russia; 3 Scientific Clinical Department, Ural Research Institute of Dermatovenereology and Immunopathology, Yekaterinburg, Russia; 4 Spondyloarthritis and Psoriatic Arthritis Laboratory, V.A. Nasonova Research Institute for Rheumatology, Moscow, Russia; 5 Head of Institution, Republican Clinical Dermatology and Venereology Clinic, Kazan, Russia; 6 Diagnostic Center, Siberian Regional Medical Center of the Federal Medical and Biological Agency, Novosibirsk, Russia; 7 Dermatovenerology Department, Tver State Medical University, Tver, Russia; 8 Dermatovenerology Department, Kazan State Medical University, Kazan, Russia; 9 Department of Dermatology, Clinical Dermatology and Venereology Clinic of the Ministry of Healthcare of Krasnodar Region, Krasnodar, Russia; 10 Department of Dermatology, Vitebsk Regional Clinical Center for Dermatology, Venereology and Cosmetology, Vitebsk, Republic of Belarus; 11 Department of Dermatology, Mogilev Regional Dermatology and Venereology Clinic, Mogilev, Republic of Belarus; 12 Department of Dermatology, City Clinical Dermatology and Venereology Clinic, Minsk, Republic of Belarus; 13 Clinical research department, JSC BIOCAD, Saint Petersburg, Russia; Monash University, AUSTRALIA

## Abstract

**Introduction:**

The objective of this study was to demonstrate that BCD-057 is similar to innovator adalimumab (iADA) in terms of efficacy, safety, and pharmacokinetics in steady state in the target population of patients with moderate to severe plaque psoriasis (NCT02762955).

**Methods:**

Patients were randomized in 1:1 ratio to receive 80 mg of BCD-057 or iADA at week 0 and 40 mg thereafter every other week from week 1. At week 24 patients from iADA group were re-randomized (1:1) to continue iADA or to be switched to BCD-057. The primary efficacy endpoint was 75% improvement in Psoriasis Area and Severity Index from baseline (PASI 75), secondary endpoints included PASI percent improvement and relative change in affected Body Surface Area (BSA) from baseline at weeks 16, 24, 33, and 55. Safety was assessed through monitoring of adverse events (AEs) and antidrug antibodies. Pharmacokinetics was evaluated at steady state.

**Results:**

Overall, 346 adult patients were included in the study (174/172 patients in BCD-057/iADA arms, respectively). At week 16 PASI 75 was achieved by 60.34% and 63.37% of patients in BCD-057 and iADA arms, respectively (p = 0.5622). Bounds of the calculated 95% confidence interval (CI) for the difference between PASI 75 responses in arms [-13.26%; 7.2%] fall within the equivalence margin [-15% to 15%] demonstrating equivalent efficacy of BCD-057 and iADA. At week 55 81.61%, 85.56%, and 80.49% of patients in BCD-057, iADA and iADA/BCD-057 arms achieved PASI 75. Comparison of the secondary endpoints did not show significant differences between arms. A comparable pharmacokinetics was shown at steady state. Safety profiles and proportions of patients with antidrug antibodies were similar between arms. The switch from the iADA to BCD-057 did not affect the immunogenicity profile.

**Conclusion:**

Obtained data demonstrate that BCD-057 and iADA are highly similar in clinical efficacy, pharmacokinetics, safety, and immunogenicity in patients with moderate to severe plaque psoriasis.

## Introduction

Biosimilars are biologic products that have no clinically meaningful differences in terms of quality attributes, efficacy, safety, and immunogenicity (IG) compared with an existing licensed originator biologic [1].

The number of biosimilars is growing every year [2]. This tendency is directly related to the need of improving patient access to biological treatment [3]. In patients with chronic diseases receiving lengthy maintenance treatment, it is especially important that switching from originator to biosimilar or vice versa has no impact on the efficacy and safety of treatment [4].

The regulatory agencies as EMA, FDA, and WHO have released guidelines for non-clinical and clinical development of biosimilars [5–7]. All these guidelines recommend a step-wise approach to demonstrate the high similarity of a proposed biosimilar to a reference product. Similarity must be shown for structural, functional, and physicochemical characteristics, pharmacokinetics (PK), clinical efficacy, and safety. Long-term clinical trials with switching between arms are crucial for demonstrating that biosimilars are effective and safe for patients who will receive maintenance treatment.

Adalimumab is a fully human recombinant monoclonal antibody with the amino acid sequence of constant domains similar to that of human IgG1. Adalimumab selectively binds to tumor necrosis factor alpha (TNFα) and neutralizes its biological functions by preventing it from interacting with the cell surface receptors p55 and p75.

Multiple controlled clinical trials demonstrated that adalimumab significantly improves the efficacy of standard treatment and is characterized by a good safety profile [8–10]. Adalimumab has the highest number of approved indications for medical use among TNFα inhibitors; some of these indications are unique to adalimumab (non-infectious uveitis, hidradenitis suppurativa). Innovator adalimumab was approved worldwide for the treatment of several inflammatory diseases, including plaque psoriasis, psoriatic arthritis, rheumatoid arthritis, juvenile idiopathic arthritis, ankylosing spondylitis, hidradenitis suppurativa, ulcerative colitis, Crohn’s disease, non-infectious uveitis, and Behcet’s disease (in some countries). Also, adalimumab has a wide range of pediatric indications [11].

BCD-057 is highly similar to the innovator adalimumab in terms of structural, functional, physicochemical, and pharmacokinetic characteristics. A phase 1 BCD-057-1 study (NCT02395055) demonstrated a similar PK of BCD-057 and the originator in healthy volunteers after the single dose. This phase 3 study (NCT02762955) was conducted from January 2017 through October 2018 in patients with moderate to severe psoriasis for assessment of similarity in clinical efficacy, safety, IG, and PK in steady state.

## Methods

**Fig 1:**
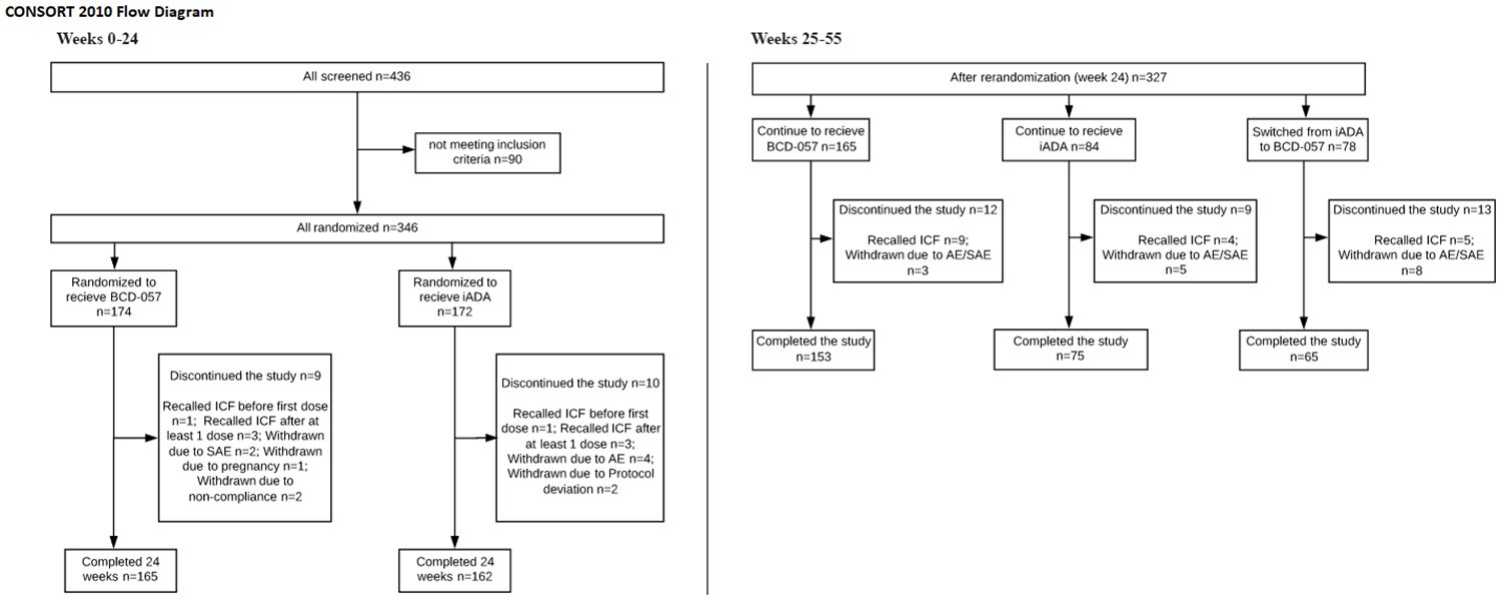
The CONSORT flow diagram.

BCD-057-2/CALYPSO is a phase 3 multicenter randomized double-blind comparative clinical study. The study protocol and supporting CONSORT checklist are available as supporting information; see S1 Checklist and S1 File. The CONSORT flow diagram is available as Fig 1.

The study has been conducted in full compliance with the Declaration of Helsinki and ICH GCP. Regulatory and ethical review board approvals from competent authorities in each country were obtained prior to study initiation. The list with full names of the ethics committees is provided in the supplementary appendix (S2 File). Patients were recruited from January 2017 through August 2017 in the Russian Federation and the Republic of Belarus at 29 sites. The participating study sites are listed in the supplementary appendix (S2 File).

All patients gave their written informed consent (ICF) before entering the study. The study enrolled patients from 18 to 75 years old with moderate to severe plaque psoriasis diagnosed for at least 6 months before ICF signing. The subjects were eligible if they had body surface area (BSA) affected by psoriasis ≥ 10%, psoriasis area and severity index (PASI) score ≥ 12, and static Physician’s Global Assessment (sPGA) score ≥ 3. Subjects previously treated with one therapeutic monoclonal antibody (or antibody fragment) against targets other than TNFα were permitted to participate after a washout period of at least 12 weeks. Main exclusion criteria were erythrodermic or pustular psoriasis or any other skin diseases (e.g. eczema) that can affect and/or complicate the assessment of psoriasis treatment with the investigational products, cancer, tuberculosis (TB) (current or in the past) and other systemic infectious diseases, previous treatment with TNFα inhibitors, use of two or more monoclonal antibodies (or their fragments) against other targets.

During the study, the use of systemic (oral or parenteral) glucocorticoids, systemic retinoids (acitretin), systemic non-biologic medications for the treatment of psoriasis and phototherapy was prohibited. A washout period of 4 weeks was required for methotrexate, sulfasalazine, cyclosporine, mycophenolate mofetil or apremilast and 6 weeks for leflunomide or cyclophosphamide. All types of phototherapy had to be discontinued for at least 4 weeks before signing the ICF. Additional details of patient eligibility criteria are provided in the supplementary appendix S1 File. Based on the subject baseline characteristics it can be stated that the selected sample is suggested as a representative of a real-world population of patients with moderate to severe psoriasis.

The following significant protocol deviations were identified: four patients underwent TB test before screening; one patient was approved for inclusion in the study 2 months and 25 days after completing another clinical study (secukinumab); two patients were approved for inclusion with the electrocardiography results obtained during their regular checkups within 1 week before signing the ICF; one patient was approved for inclusion with the fluorography obtained 1 month and 2 days before the ICF signing; for eleven patients the screening period was extended by 2–4 days and for one patient—for 9 days due to delayed laboratory results.

### Patients

**Fig 2:**
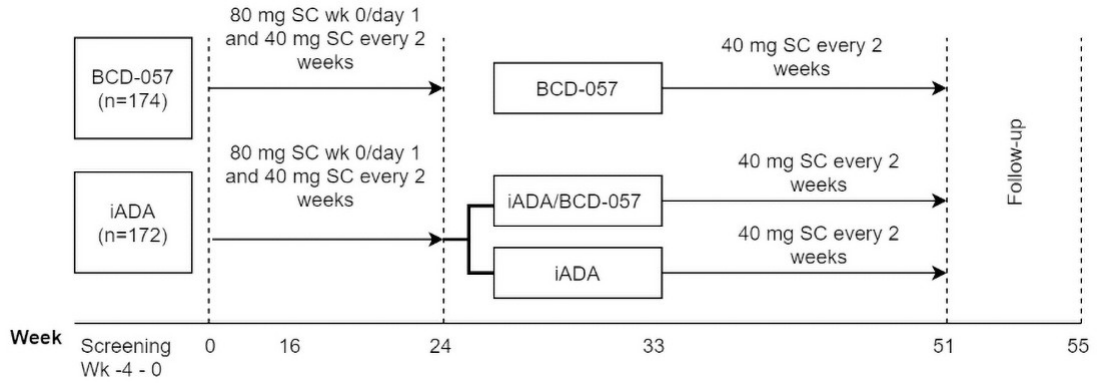
Study design.

After the screening period patients were randomized in 1:1 ratio in a double-blind manner to receive 80 mg of BCD-057 or innovator adalimumab (iADA) at week 0 and 40 mg thereafter every other week (EOW) from week 1 until week 24. At week 24 patients from iADA group were re-randomized in 1:1 ratio: subgroup 1 continued to receive iADA, subgroup 2 switched to BCD-057 at a 40 mg EOW till the week 51 (the treatment was blinded during the whole study). Observation of patients was carried out until week 55 (Fig 2).

Efficacy, IG, and safety were assessed throughout the study. Blood samples for PK were collected from baseline up to week 23.

### Efficacy evaluation

The primary endpoint was the proportion of patients who reached PASI 75 response at week 16 (75% improvement in PASI from baseline). The secondary endpoints included PASI 75 at weeks 24, 33, and 55, PASI percent improvement and relative change in affected BSA from baseline at weeks 16, 24, 33, and 55.

The efficacy endpoints were assessed in two arms (at weeks 16, 24) before switching the therapy and thereafter in three arms (at weeks 33, 55) after switching (Fig 2).

### Safety evaluation

Adverse events (AE) were reported according to Common Terminology Criteria for Adverse Events (CTCAE), v. 4.03 [12]. Safety was assessed via monitoring for treatment-related AEs (TAE’s), serious AEs (SAEs), AEs of interest (related to the use of TNFα blockers: infections, malignancies, hypersensitivity, laboratory abnormalities), laboratory data, vital signs and IG (weeks 16, 33, and 55). The monitoring for TB infection was performed at baseline and weeks 25, 55 by Interferon-γ release assay (IGRA) or Diaskintest. The safety assessment was performed in two arms for the period weeks 0–24 and in three arms for the period weeks 25–55.

### Immunogenicity evaluation

Blood samples for IG assessment were collected at the baseline and weeks 16, 33, 55. IG analysis included testing for binding (bAbs) and neutralizing (nAbs) antibodies to adalimumab. BAbs were evaluated using a validated enzyme-linked immunosorbent assay (ELISA). The assessment for nAbs was performed only for patients with detected bAbs. Neutralizing assay in WEHI-13VAR cell culture was used.

### Pharmacokinetics evaluation

Secondary PK endpoints included AUC_tau,ss_ (area under the concentration-time curve during a dosing interval at steady state), C_av,ss_ (average steady-state serum drug concentration), C_max,ss_ (maximum serum concentration at steady state), C_min,ss_ (minimum effective serum drug concentration at steady-state), C_trough_ (measured concentration at the end of a dosing interval at steady state (taken directly before next administration)) after multiple subcutaneous injections of iADA or BCD-057. Blood samples for serum adalimumab assessment were drawn pre-dose at week 15 and then after 3, 12, 24, 48, 72, 96, 120, 144, 192, 240, 288 hours after administration and pre-dose at weeks 16, 17, 19, 21, 23.

### Statistical analysis

The purpose of this study was to test the hypothesis of BCD-057 being equivalent to iADA. The hypothesis was tested with the following error values: 5% type 1 error rate; 20% type 2 error rate (power of 80%). The 95% two-tailed confidence interval (CI) for the difference between PASI 75 responses in BCD-057 group and iADA group was compared with equivalence interval [-15%; 15%] (pre-defined equivalence margin 15%). Efficacy of BCD-057 will be equivalent to iADA if the 95% CI for the difference between PASI 75 responses falls within the equivalence interval. The sample size was determined using the formula for equivalence trials [13]. PASI 75 response rate at week 16 for both arms was used as equal to the rate from the iADA study M03-656 (70.9%) [14]. With an account for 5% to 10% potential withdrawals, the number of patients was 172 subjects per arm. The statistical analysis was performed using two-tailed hypothesis tests. The efficacy evaluation was provided in two populations: the ITT (intent-to-treat) population (all randomized patients, n = 346) and the PP (per protocol) population (included subjects who had no major protocol deviations up to and including week 16, n = 342). This article includes results in the ITT population. For the efficacy analysis set the last observation carried forward method was used to handle missing data. Normally distributed data were analyzed using the two-sample Student’s *t-*test, Welch’s *t*-test, and ANOVA. Non-normally distributed data were analyzed using Mann-Whitney test, Wilcoxon test, Kruskal-Wallis test, and Friedman’s test. The Shapiro-Wilk test was used to test the data for normality. The categorical data were processed using Fisher’s exact test and Yates-corrected Pearson’s chi-square test. Correction for multiple comparisons was performed with the Benjamini-Yekutieli procedure. Generalized estimating equations (GEE) approach was used for the analysis of longitudinal data for iADA and BCD-057 arms.

The safety analysis set included all randomized patients (n = 346). The IG population included patients who received at least one injection of study drug and provided at least two blood specimens for testing, one of which was taken before the first dosing, and the second one–at one of the subsequent visits. At weeks 16 the population included 168 patients in BCD-057 and 162 patients in iADA arms, at week 33–159, 77, and 69 patients in BCD-057, iADA and iADA/BCD-057 arms, at week 55–152, 75, and 65 in the same arms respectively. The seroconversion among patients bAb-negative at week 16 was evaluated in iADA (n = 68) and iADA/BCD-057 (n = 55) arms.

The PK in steady state was analyzed in a limited number of patients (150 patients: 76 in BCD-057 arm, 74 patients in the reference arm). The PK population included patients who had PK blood sample taken before the first injection, completed all visits for PK assessment starting from Day 1 of week 15 to Day 6 of week 16 and did not miss more than two PK blood samplings during this period.

## Results

### Patients

A total of 346 patients (174 and 172 in BCD-057 and iADA arms respectively) with moderate to severe psoriasis were randomized in the study to receive subcutaneous 80 mg of BCD-057 or iADA on week 0 and 40 mg thereafter EOW. A total of 327 patients completed 24 weeks, 293 patients completed 55 weeks of the study (Fig 1). The reasons of discontinuation were ICF withdrawal, AEs, pregnancy, poor treatment compliance, and protocol deviations (Fig 1). Two patients discontinued the study before the first injection due to non-safety reasons (recalled ICF).

Demographic and baseline psoriasis characteristics did not differ significantly between arms (Table 1). The number of included males was significantly higher than females in both arms. All patients were white except for one patient in the iADA arm who was a mixed race (white/black).

**Table 1:** Main demographic and baseline psoriasis characteristics (ITT population, n = 346).

Variables	BCD-057 (N = 174)	iADA (N = 172)
Age, years	42.5 [34–50]	42.5 [32–52]
White race*	173 (99.42)	172 (100)
Mixed race (white/black)*	1 (0.58)	0
Women*	61 (35.06)	58 (33.72)
Men*	113 (64.94)	114 (66.28)
BMI, kg/m^2^	27 [24–31]	26 [24–30]
Psoriasis duration, mo.	120 [49–204]	132 [52–240]
PASI, total score	23.3 [17.4–35.1]	24.15 [19.3–35.3]
BSA, %	32.5 [18–54.1]	33 [22–51]
sPGA		
Grade 3 (moderate)*	76 (43.68)	75 (43.60)
Grade 4 (severe)*	86 (49.43)	83 (48.26)
Grade 5 (very severe)*	12 (6.89)	14 (8.14)
Previous use of methotrexate*	51 (29.65)	44 (25.29)
Previous use of systemic non-biologics (other than methotrexate)*	89 (51.74)	97 (55.75)
Previous use of phototherapy*	82 (47.67)	73 (41.95)
Previous use of biologic DMARDs*	10 (5.81)	9 (5.36)
Ustekinumab*	8 (4.88)	9 (5.36)
Secukinumab*	1 (0.61)	0
Netakimab*	1 (0.61)	0

Median and interquartile range are presented except variables marked with *, where no. (%) is presented. BMI–body mass index; PASI–Psoriasis Area and Severity Index (index ranges from 0 to 72, where 72 indicates the most severe psoriasis); BSA–body surface area affected by psoriasis; sPGA—static Physician’s Global Assessment (grades from 0 to 5, where 5 indicates the most severe grade); VAS–visual analogue scale (from 0 to 100 mm, 100 mm indicates the most pronounced manifestation); DMARD—Disease-modifying antirheumatic drugs.

### Therapeutic efficacy

#### Primary endpoint

At week 16, PASI 75 was achieved by 60.34% of 174 patients in the BCD-057 arm and 63.37% of 172 patients in the iADA arm (p = 0.5622). The bounds of the calculated 95% CI for the difference between PASI 75 responses in BCD-057 group and iADA group [-13.26%; 7.2%] fall within the equivalence margin [-15% to 15%] demonstrating equivalent efficacy of BCD-057 and iADA.

#### Secondary endpoints

**Fig 3:**
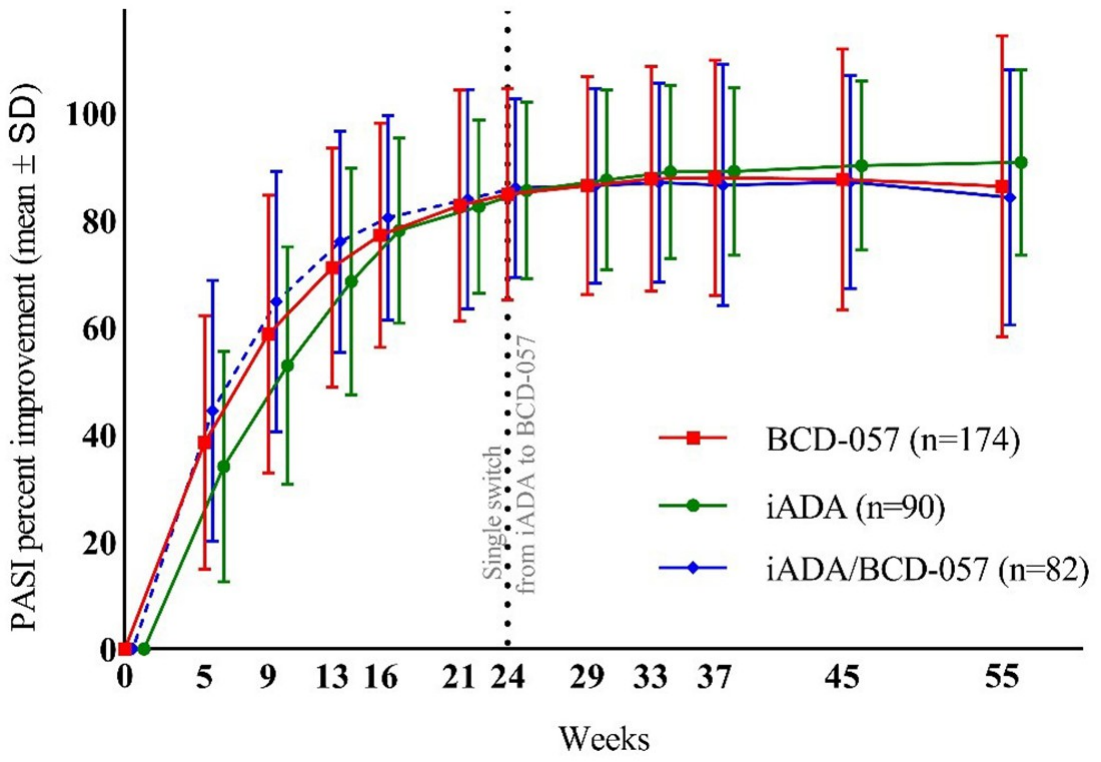
PASI percent improvement from baseline.

*Psoriasis Area and Severity Index improvement from baseline*. Mean PASI percent improvement from baseline at week 16 was 77.47% in BCD-057 arm, 80.36% in iADA arm. At week 24 improvements of 84.85% and 85.80% were observed in the same arms respectively. After re-randomization, mean PASI percent improvement from baseline was 87.74% for BCD-057, 89.01% for iADA, and 87.05% for iADA/BCD-057 at week 33. At week 55, mean PASI percent improvement was 86.32%, 90.81%, and 84.21% in BCD-057, iADA, and iADA/BCD-057 arms, respectively. No statistically significant differences in PASI percent improvement between study arms were detected at any time-point (Fig 3).

**Fig 4:**
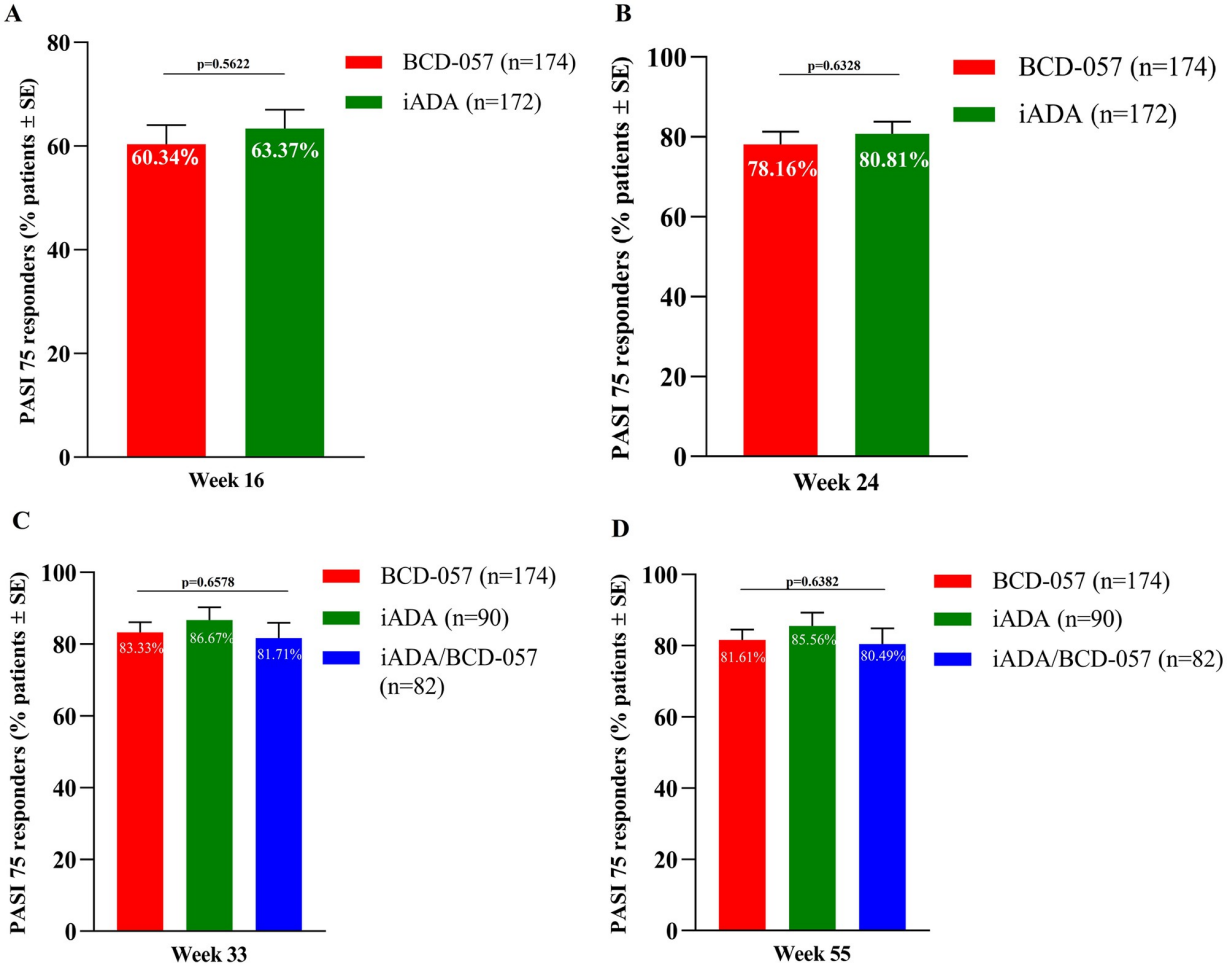
PASI 75 response rate. (A) PASI 75 at week 16. (B) PASI 75 at week 24. (C) PASI 75 at week 33. (D) PASI 75 at week 55.

*Psoriasis Area and Severity Index response*. At week 24, PASI 75 was reached by 78.16% of 174 patients in the BCD-057 and 80.81% of 172 patients in the iADA arm (Fig 4B). After re-randomization, at weeks 33 and 55 no statistically significant differences between BCD-057 or iADA/BCD-057 arms vs. the iADA arm were detected (Fig 4B and 4C).

*Changes from baseline in affected Body Surface Area*. The BSA affected by psoriasis decreased significantly by week 16 in all groups. The mean relative changes in affected BSA from baseline to week 16 and 24 were 64.70% and 76.66% in BCD-057 arm, 57.55% and 77.15% in iADA arm. At week 33 the means in re-randomized arms were 84.27%, 84.09%, and 80.80%, at week 55–83.94%, 88.11%, and 79.85% in BCD-057, iADA and iADA/BCD-057, respectively.

**Fig 5:**
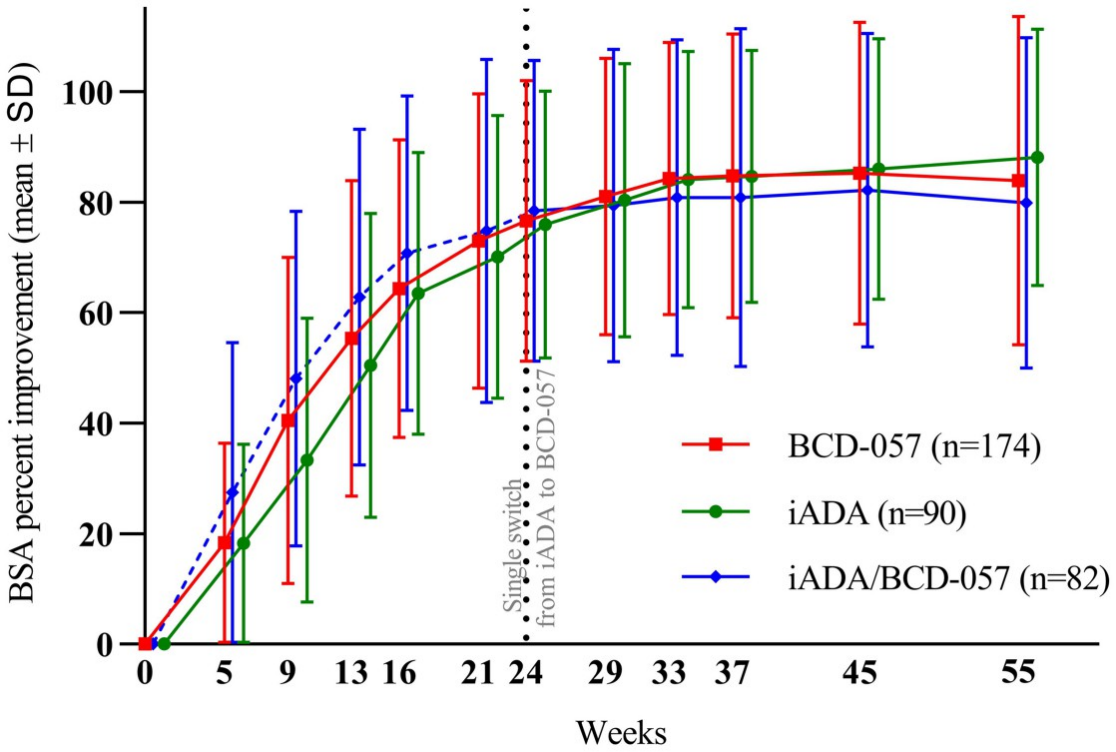
Changes from baseline in affected BSA (%).

No statistically significant differences between the study arms were revealed in relative BSA change at any time-points (Fig 5).

### Safety

Incidence rates of AEs were similar in groups during the study. There were no unexpected AEs in any arm (Tables 2 and 3).

**Table 2:** Safety profile week 0–24.

Variables	BCD-057 (n = 174)		iADA (n = 172)		*P* value
	n	%	n	%	
Therapy related AEs	54	31.03	54	31.40	0.7785*
Therapy related AEs (grade 3–4)	4	2.30	10	5.81	0.1095†
AEs of interest	53	30.46	54	31.40	0.5542†
Infections	14	8.05	14	8.14	1.0000*
Serious infections	0	0.00	3	1.74.	0.1218†
Opportunistic infections	4	2.30	10	5.81	0.1218†
Malignant neoplasms	0	0.00	0	0.00	1.0000†
Allergic reactions	0	0.00	0	0.00	1.0000†
Complete blood count abnormalities	26	14.94	21	12.21	0.5310†
Blood biochemistry abnormalities	14	8.05	19	11.05	0.3655†
**Summary of severe AEs**					
Blood pressure increased (grade 3)	2	1.16%	4	2.30%	0.6848†
Hyperglycemia (grade 3)	1	0.58%	1	0.57%	1.0000†
Toxicoderma (grade 3)	0	0.00%	1	0.57%	1.0000†
Creatinine increased (grade 3)	1	0.58%	0	0.00%	0.4971†
Proteinuria (grade 3)	1	0.58%	0	0.00%	0.4971†
Arthritis (grade 3)	0	0.00%	1	0.57%	1.0000†
Head injury (grade 5)	0	0.00%	1	0.57%	1.0000†
Prostate cancer (grade 3)	1	0.58%	0	0.00%	0.4971†
Tuberculosis (grade 4)	0	0.00%	1	0.57%	1.0000†

Note:

* Yates-corrected Pearson’s chi-square test;

^†^ Fisher’s exact test.

AE–adverse event.

**Table 3:** Safety profile weeks 25–55.

Variables	BCD-057 (n = 174)		iADA (n = 90)		iADA/BCD-057 (n = 82)		*P* value
	n	%	n	%	n	%	
Therapy related AEs	29	16.67	15	16.67	12	14.63	0.9091*
Therapy related AEs (grade 3–4)	4	2.30	2	2.22	1	1.22	1.0000†
AEs of interest	28	16.09	4	15.56	14	17.07	0.9626†
Infections	16	9.20	6	6.67	4	4.88	0.4933†
Serious infections	1	0.57	0	0.00	0	0.00	1.0000†
Opportunistic infections	4	2.30	2	2.22	1	1.22	1.0000†
Malignant neoplasms	0	0.00	0	0.00	0	0.00	1.0000†
Allergic reactions	0	0.00	0	0.00	0	0.00	1.0000†
Complete blood count abnormalities	6	3.45	5	5.56	5	6.10	0.5302†
Blood biochemistry abnormalities	6	3.45	3	3.33	5	6.10	0.5199†
**Summary of severe AEs**							
Cystitis (grade 3)	0	0.00	1	1.11	0	0.00	0.4971†
Cellulitis of anterior abdominal wall (grade 4)	1	0.57	0	0.00	0	0.00	1.0000†
Hyperglycemia (grade 3)	1	0.57	0	0.00	0	0.00	1.0000†
Ankle fracture (grade 4)	1	0.57	0	0.00	0	0.00	1.0000†
Bruise (grade 3)	0	0.00	0	0.00	1	1.22	0.2370†
Left femoral neck fracture (grade 4)	1	0.57	0	0.00	0	0.00	1.0000†
Endometrium cancer (grade 3)	0	0.00	1	1.11	0	0.00	0.4971†
Neutropenia, grade 4	0	0.00	1	1.11	0	0.00	0.4971†
Thrombocytopenia (grade 3)	1	0.57	0	0.00	0	0.00	1.0000†
ALT increased (grade 3)	2	1.15	1	1.11	1	1.22	1.0000†
GGT increased (grade 3)	0	0.00	1	1.11	0	0.00	0.4971†
AST increased (grade 3)	1	0.57	0	0.00	0	0.00	1.0000†
Hyperbilirubinemia (grade 3)	0	0.00	1	1.11	0	0.00	0.4971†
Functional CNS disorder (grade 3)	0	0.00	1	1.11	0	0.00	0.4971†

Note:

* Yates-corrected Pearson’s chi-square test;

^†^ Fisher’s exact test.

AE–adverse event; ALT—alanine aminotransferase; GGT—gamma-glutamyl transferase; AST—aspartate aminotransferase; CNS—central nervous system.

### Safety assessment (weeks 0–24)

During the first 24 weeks TAEs were found in 31.03% (54/174) and 31.40% (54/172) of patients in the BCD-057 and iADA arms, respectively, severe AEs (CTCAE v.4.03 grade 3–4 AEs)—in 2.30% (4/174) and 5.81% (10/172) of patients in the same arms.

SAEs were recorded in five patients (2.87%) in BCD-057 arm (grade 3 toxicodermia, grade 3 post-traumatic osteoarthritis of interphalangeal joint; grade 2 chicken pox; grade 5 head injury resulting in death; grade 2 acute bilateral purulent upper-jaw sinusitis). Of these, only chickenpox and purulent upper-jaw sinusitis were registered as TAE’s. One case of treatment-related SAE was registered in iADA arm (grade 4 disseminated pulmonary TB at the infiltration stage, Mtb (-), 1-A). Most common AEs were blood and lymphatic system disorders (lymphocytosis, neutropenia), hepatobiliary disorders (hyperbilirubinemia, transaminases increased, gamma glutamyl transpeptidase (GGT) increased), cardiovascular disorders (arterial hypertension), and infections (respiratory tract infections, tonsillitis, urinary tract infection). During the first 24 weeks of the study no statistically significant differences were revealed between BCD-057 and iADA in the incidence of any AEs.

### Safety assessment (weeks 25–55)

During this period new TAEs were registered in 16.67% (29/174) of patients in the BCD-057 arm, 16.67% (15/90) of patients in the iADA arm, and 14.63% (12/82) of patients in the iADA/BCD-057 arm (Table 3).

One treatment-related SAE (cellulitis of anterior abdominal wall) and two non-treatment-related SAEs (displaced bimalleolar fracture of the left ankle joint and closed displaced fracture of left femoral neck) were reported for the BCD-057 arm. One SAE unrelated to study therapy was recorded in the iADA arm (car accident injury). In the iADA/BCD-057 arm, two unrelated to treatment SAEs were recorded: functional central nervous system disorder and endometrioid carcinoma G1.

Most common AEs were blood and lymphatic system disorders (lymphocytosis, neutropenia), hepatobiliary disorders (hyperbilirubinemia, transaminases increased, GGT increased), infections (respiratory tract infection, cystitis, positive IGRA test result), metabolism disorders (hyperglycemia) and general disorders (flu-like syndrome). Other AEs were reported in single cases. The AEs were mostly mild to moderate in severity (grade 1/2, CTCAE 4.03).

### AEs of special interest

At weeks 0–24 AEs of special interest were identified in 30.46% (53/174) of patients in BCD-057, 31.40% (54/172) in iADA arms. At weeks 25–55, AEs of special interest were observed in 16.09%, 15.56%, and 17.07% of patients in the BCD-057, iADA, and iADA/BCD-057 arms, respectively. The incidence of AEs of special interest was comparable in both study periods.

The AEs at weeks 0–24 were presented with infections (8.05% vs 8.14% in BCD-057 and iADA arms), including serious infections (1.74% in iADA), opportunistic infections (2.30% vs 5.81% in BCD-057 and iADA); hematology (14.94% vs 12.21%) and biochemistry abnormalities (8.05% vs 11.05%). At weeks 25–55 infections were reported for 9.20%, 6.67%, 4.88% of patients in BCD-057, iADA, iADA/BCD-057 arms, respectively, including opportunistic infections in 2.30%, 2.22%, and 1.22% in the same arms. 0.57% in BCD-057 arm had serious infections. Hematologic abnormalities were recorded in 3.45%, 5.56%, and 6.10% of patients, biochemistry abnormalities–in 3.45%, 3.33%, and 6.10% in BCD-057, iADA, and iADA/BCD-057 arms, respectively.

There were no cases of malignancies identified as TAEs.

Prior to switching (weeks 0–24) one patient from the iADA arm was diagnosed with prostate cancer (grade 3 unrelated to treatment). After the switching (weeks 25–55) benign scrotum neoplasm (grade 2) and endometrioid carcinoma G1 (grade 3) were found in iADA arm (both unrelated to treatment). No cases of allergic reactions were recorded during the study.

During the study (weeks 0–55), positive test result for M. tuberculosis were obtained in eight patients from BCD-057 arm, nine patients from iADA arm, and one patient from iADA/BCD-057 arm. Positive tests for M. tuberculosis were registered as TAEs, patients discontinued the study. In three patients in iADA arm were diagnosed with the active TB (one SAE grade 4 and two TAEs grade 2). TB was confirmed by the chest X-ray and TB specialist consultation. There were no significant differences in the incidence of active TB or positive tests for TB between the study arms.

### Immunogenicity

Proportion of bAb and nAbs positive patients did not differ between study arms at all tested time points. To compare the impact of IG on the treatment efficacy across study arms, a stratified analysis was conducted. Stratification was performed based on PASI 50 achievement. The proportions of patients with bAbs and nAbs among patients with low treatment response (≤PASI 50) did not differ significantly at weeks 16, 33, and 55 between treatment arms (Table 4).

**Table 4:** Immunogenicity profile.

Week	BCD-057	iADA		p-value*
		iADA	iADA/BCD-057	
**Binding antibodies, all tested**				
Week 16	43 / 168 (25.60%)	39 / 162 (24.07%)†		0.7492
		16 / 84 (19.05%)	23 / 78 (29.49%)	0.2919
Week 33	70 / 159 (44.03%)	35 / 77 (45.45%)	42 / 69 (60.87%)	0.0556
Week 55	85 / 152 (55.92%)	38 / 75 (50.67%)	44 / 65 (67.69%)	0.1146
**Binding antibodies, ≤PASI 50**				
Week 16	6 / 16 (37.5%)	1 / 7 (14.29%)†		0.1044
		0 / 2 (0)	1 / 5 (20.0%)	0.1143
Week 33	5 / 5 (100%)	2 / 2 (100%)	2 / 2 (100%)	0.8736
Week 55	9 / 10 (90.0%)	2 / 3 (66.67%)	6 / 6 (100%)	0.6519
**Neutralizing antibodies, all tested**				
Week 16	4 / 168 (2.38%)	7 / 162 (4.32%)†		0.3358
	4 / 168 (2.38%)	1 / 84 (1.19%)	6 / 78 (7.69%)	0.1283
Week 33	12 / 159 (7.55%)	6 / 77 (7.79%)	8 / 69 (11.59%)	0.9633
Week 55	10 / 152 (6.58%)	6 / 75 (8.0%)	5 / 65 (7.69%)	0.8228
**Neutralizing antibodies, ≤PASI 50**				
Week 16	1 / 16 (6.25%)	0 / 7 (0)†		0.147
	1 / 16 (6.25%)	0 / 2 (0)	0 / 5 (0)	0.166
Week 33	2 / 5 (40.0%)	0 / 2 (0)	1 / 2 (50.0%)	0.863
Week 55	1 / 10 (10.0%)	1 / 3 (33.33%)	2 / 6 (33.33%)	0.4153

n / N (%) are presented.

* Yates-corrected Pearson’s chi-square test, Cochran–Mantel–Haenszel test, Fisher’s exact test.

^†^ pooled iADA data at week 16 are presented.

To evaluate the effect of switching on bAb and nAbs seroconversion rate the subanalysis in patients without bAbs at week 16 from iADA and iADA/BCD-057 arms was performed (Table 5). The rates of seroconversion in patients receiving iADA and those who were switched to BCD-057 at week 25, were comparable.

**Table 5:** Seroconversion rates among patients bAb-negative at week 16.

Variables	iADA	iADA/BCD-057	p value*
**Binding antibodies**			
Weeks 33, 55	36 / 68 (52.94%)	31 / 55 (56.36%)	0.7198
Week 33	23 / 68 (33.82%)	23 / 55 (41.42%)	0.4538
Week 55	28 / 68 (41.18%)	27 / 55 (49.09%)	0.4661
**Neutralizing antibodies**			
Weeks 33, 55	7 / 68 (10.29%)	3 / 55 (5.45%)	0.5094
Week 33	5 / 68 (7.35%)	3 / 55 (5.45%)	1.0000
Week 55	3 /68 (4.41%)	1 / 55 (1.82%)	0.6194

n / N (%) are presented.

* Fisher’s exact test.

The arms did not differ in the proportions of bAb and nAb-positive patients during 55 weeks of the study. Overall, the immunogenicity profile and impact of seroconversion on maintenance of treatment effect were similar between arms with or without switching from iADA to BCD-057.

### Pharmacokinetics

**Fig 6:**
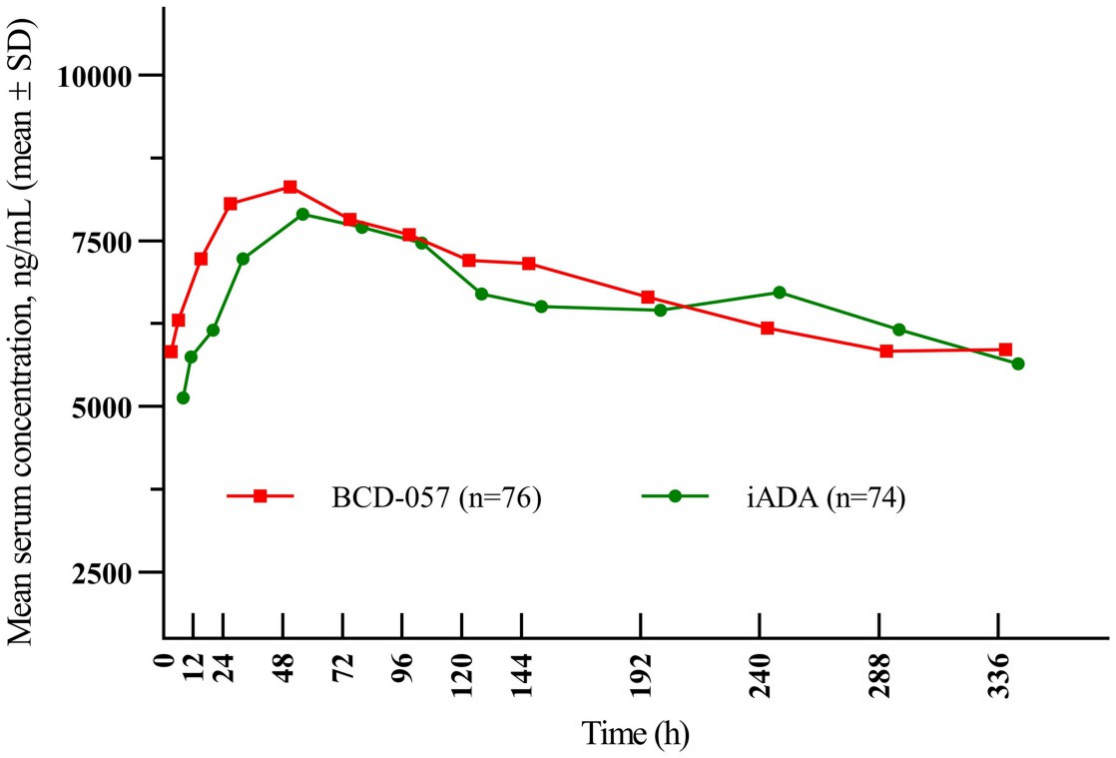
Adalimumab concentrations (ng/mL) after multiple administration of BCD-057 or iADA.

The analysis showed that steady-state serum concentrations changed in the similar way after the use of BCD-057 and iADA (Fig 6). The 90% CI for the AUC_tau,ss_ geometric mean ratio was 81.46% to 124.40% and lays within the pre-specified PK equivalence range of 80% to 125%. After multiple subcutaneous injections of adalimumab, median C_av,ss_ was 6236.9 ng/mL for BCD-057 and 6089.3 for iADA, median C_max,ss_ was 8940.2 and 8079.9 ng/mL for BCD-057 and iADA, median C_min,ss_ was 4334.2 and 4162 ng/mL, median C_trough_ was 5328.3 and 4788.5 ng/mL. There were no statistically significant differences between the arms in any steady-state PK endpoint after multiple dosing.

## Discussion

Moderate to severe plaque psoriasis was chosen as the optimal model for demonstration of biosimilarity. IADA was studied in the wide range of indications such as plaque psoriasis, rheumatoid arthritis, ankylosing spondylitis, and Crohn disease [15–21]. The highest effect size of iADA versus placebo was demonstrated in the plaque psoriasis trials. Furthermore, skin responses to treatment are relatively rapid with results that can be easily accessed and quantified [22]. Other iADA indications required the concomitant treatment, which can have impact on the efficacy and the IG assessment. Based on this, the use of adalimumab monotherapy was preferred.

The study used efficacy criteria and measures that were already applied in other large-scale clinical trials with systemic medications for the treatment of psoriasis [23, 24]. The primary endpoint, PASI 75 response at week 16, was shown to be equivalent for BCD-057 and iADA: the 95% CIs for treatment difference were within the predefined margins for equivalence of ±15%. A single switching from iADA to BCD-057 had no impact to the efficacy assessment.

The safety profile was comparable between study arms prior and after therapy switching. The most frequently observed AEs were blood and lymphatic disorders (lymphocytosis, neutropenia), hepatobiliary disorders (hyperbilirubinemia, transaminases increased, GGT increased) and infections (respiratory tract infections). BCD-057 and iADA were generally well tolerated.

TNFα inhibitors are known to have increased risks of infections (including opportunistic infections), malignant neoplasms, allergic reactions, and laboratory abnormalities, also defined as AEs of special interest. In BCD-057-2 study the incidence rate of AEs of special interest did not differ between arms for both time periods.

The positive tests for TB and the cases of active TB were observed during the study. The cases of active TB have been confirmed by the chest X-ray and TB specialist consultation. According to SMPC of iADA and literature data of other TNFα blockers, an reactivation and new onset of TB were observed in patients receiving iADA [25–27]. Thus, the risk of TB was expected. Summarizing all said above, no new safety signals were detected in this study and incidences of AEs were generally balanced between treatment arms.

The IG analysis demonstrated no statistically significant difference in the proportions of bAb/nAb-positive patients at any time point. The loss of response due to the antibodies formation over time with TNFα blockers therapy is known and has been previously reported [28, 29]. The analysis of bAbs and nAbs among patients who achieved PASI 50 and less showed the comparability of the arms. Over time the proportions of bAb/nAb-positive patients increased. After the switch from BCD-057 to iADA, there was no increase in the proportion of patients with bAbs de novo vs. patients who received only iADA. The rates of seroconversion among patients bAb-negative at week 16 were comparable between iADA and iADA/BCD-057 arms.

The PK evaluation showed that steady-state serum concentrations of adalimumab changed the similar way after the use of BCD-057 and iADA with no statistically significant differences between arms in such parameters as AUCtau,ss, Cav,ss, Cmax,ss, Cmin,ss and Ctrough.

### Strength and limitations of the study

Our study had several strengths: a double-blind approach and an equivalence design in accordance with EMA’s recommendations [30, 31]; selection of the moderate-to-severe psoriasis as the model for demonstration of biosimilarity; the standard well known endpoints with clear assessment characteristics; switching therapy from originator to biosimilar; representative study population balanced between arms.

Our study also had some limitations. It is important to note that all these limitations did not distort the BCD-057-2/CALYPSO study results. The first limitation is the small number of participants compared to some adalimumab biosimilar studies [32, 33]. However, the sample size was suitable to detect statistically significant differences or prove their absence with pre-defined equivalence margin 15% and power 80%. As the efficacy measure was the primary endpoint in this study, the number of patients required to evaluate efficacy was determinative. Approximately the same number of patients was studied in the pivotal phase 3 clinical trial of the first adalimumab biosimilar ABP-501 [34]. Another limitation is the use of last observation carried forward method to handle missing data for efficacy analysis. Regarding to insignificant number of missed visits the use of this method was appropriate. A limitation also is that there was no assessment of the efficacy after one year of the treatment. The data of iADA showed maintenance of efficacy for over 3 years in the REVEAL study: 76% of sustained responders (patients who achieved PASI 75 and more at weeks 16 and 33 during the main study) reached PASI 75 after 160 weeks of continuous therapy in the open-label extension. And some patients with less than PASI 75 responses in the main study also achieved long-term PASI 75 responses [35]. BCD-057 is expected to show similar long-term efficacy, but further observation is needed.

## Conclusions

BCD-057 and iADA were shown to be equivalent in terms of PASI 75 response at week 16 in moderate to severe plaque psoriasis patients. BCD-057 was well tolerated and comparable to iADA in terms of one-year efficacy, IG, steady-state PK, and safety with no apparent impact of therapy switching.

## Supporting information

S1 Checklist (PDF)

S1 File (PDF)

S2 File (PDF)
