# Supplementary material for: Long-term data on the proposed adalimumab biosimilar BCD-057 in patients with moderate to severe psoriasis: A randomized controlled trial
Source: PLoS One. 2022 Feb 7;17(2):e0263214. doi: 10.1371/journal.pone.0263214 (PMC8820628; doi:10.1371/journal.pone.0263214)
Supplement: S1 File — (PDF) [file pone.0263214.s002.pdf]

Protocol S2

This appendyx contains the final clinical study protocol. It has been provided by the authors to give readers additional information about their work.

Protection of Privacy and Confidentiality

This is a cover page for JSC BIOCAD document that has been redacted for either Personal Information (PI) or Company Confidential Information (CCI) or both. Within this document PI or CCI is either removed or redacted (i.e., specific content is masked irreversibly from view with a black bar) to protect personal privacy or intellectual property.

CLINICAL STUDY PROTOCOL

**Protocol Title:**	An International Multicenter, Randomized, Double-Blind Study Comparing the Efficacy and Safety of BCD-057 (INN: Adalimumab, JSC BIOCAD, Russia) and Humira® (INN: Adalimumab, Vetter Pharma-Fertigung GmbH & Co. KG, Germany) in Patients with Moderate-to-Severe Plaque Psoriasis
**Protocol ID:**	BCD-057-2
**Protocol Date:**	December 21, 2015
**Protocol Amendment:**	No. 4
**Date of Protocol Amendment:**	March 05, 2018
**Protocol version:**	v. 4.1
**Study Sponsor/Monitor:**	JSC BIOCAD, Russia [redacted]
[redacted]	[redacted]
[redacted]	[redacted]

The information presented in this document is confidential and intended to be used solely by investigators, ethics committee members, and health care authorities. The information contained in this protocol should not be transferred to any third party without the prior written permission from JSC BIOCAD, except when necessary for obtaining patient's consent to participate in the study.
These requirements come into effect upon signature of this Protocol.

Table of contents

Protection of Privacy and Confidentiality 2
Table of contents 4
List of tables 9
List of figures 11
Signature page 12
Abbreviations 13
Terms and definitions 15
Document history 17
Names/positions of investigators responsible for the study conduct. Contact information of the study sites 18
Name, job title, address and telephone number of the qualified physician responsible for taking medical decisions 21
Names and addresses of clinical or other medical and/or technical services and/or organizations involved in the study 22
1. Study rationale 38
1.1. Introduction 38
1.1.1. Overview of epidemiology, pathogenesis, and treatment options 38
1.1.2. Background information for studied therapy 39
1.2. Name and description of investigational products 40
1.3. Relevant non-clinical and clinical aspects 41
1.3.1. Non-clinical studies 41
1.3.2. Clinical trials 50
1.3.3. Conclusions and study rationale 62
1.4. Brief description of known and potential risks and benefit for study subjects (benefit/risk balance) 63
1.4.1. Benefit assessment 63
1.4.2. Risk assessment 63
1.4.3. Conclusions 65
1.5. Description and justification of route of administration, doses, dosing regimen and treatment course 66
1.5.1 Design description and justification 66

1.5.2. Description and justification of route of administration, doses, dosing regimen and treatment course 68
1.6. Clinical study compliance with regulatory requirements 69
1.7. Description of study population 69
1.8. References 70
2. Study objective and goals 73
2.1 Study objectives 73
2.2 Study goals 73
3. Study hypothesis 75
4. Study design 76
4.1. Primary and secondary outcome measures to be assessed in the study 76
4.1.1 Primary endpoint 76
4.1.2 Secondary endpoints 76
4.2. Description of the study type/design, study flow-chart, study procedures and periods 77
4.3. Measures to minimize/eliminate bias 82
4.3.1. Distribution of patients by study sites 82
4.3.2. Procedure of assigning study IDs 82
4.3.3. Stratification procedure 83
4.3.4. Randomization procedure 83
4.3.5. Blinding and subject-specific lots of investigational products 85
4.4. Study therapy, doses, and dosage regimens of investigational products Pharmaceutical form, packaging, and labeling of investigational products 86
4.4.1. Study therapy, doses, and dosage regimens of investigational products 86
4.4.2. Pharmaceutical form, packaging, and labeling of investigational products 87
4.5. Expected duration of the study and subjects' participation in the study 89
4.6. Study periods 89
4.6.1 Study visits and procedures 89
4.6.2. Procedures by visits 94
4.7. Description of individual study procedures 104
4.7.1. History taking, complaints, demographics 109
4.7.2. Physical examination 110
4.7.3. Vital signs 111
4.7.4. Laboratory tests 111
4.7.5. ECG 113

4.7.6. Tests for tuberculosis 114
4.7.7. Chest x-ray and fluorography 116
4.7.8. PASI and BSA assessment 116
4.7.9. Static Physician Global Assessment (sPGA) 118
4.7.10. Nail psoriasis severity index (NAPSI) 118
4.7.11. Patient assessment of itching 119
4.7.12. Quality of life assessment 120
4.7.13. Collecting blood samples for PK assessment 120
4.7.14. Blood sampling for immunogenicity assessment 124
4.7.15. Storage and shipment of PK and immunogenicity specimens 125
4.7.16. Adalimumab assay in the serum 125
4.7.17. Unscheduled visits 125
4.7.18. Data entering to eCRF 126
4.8. Stop rules and criteria for premature withdrawal for study subjects, study periods, and study as a whole 126
4.8.1. Stop rules for study as a whole 126
4.8.2. Criteria for premature withdrawal of study subjects 126
4.9. Drug accountability 127
4.9.1. Handling of investigational products 128
4.10. Procedure for keeping and unblinding randomization codes 129
4.11. Data entered directly into CRF (i.e. no prior written or electronic record of data) and considered as source data 129
5. Eligibility and exclusion of study subjects 130
5.1. Inclusion criteria 130
5.2. Exclusion criteria 131
5.3. Withdrawal criteria 134
5.4. Follow-up of subjects withdrawn from the study or subjects who discontinued the study treatment but remain in the study for follow-up 135
5.4.1. Follow-up of patients who received at least one dose of BCD-057/Humira® 135
5.4.2. Follow-up of patients who did not receive a single dose of BCD-057/Humira® 136
6. Treatment of study subjects 137
6.1. Study therapy 137
6.1.1. Treatment regimen and duration 137

6.1.2. Preparation of the investigational products for administration, and administration procedure 138
6.1.3. Adjustment and discontinuation of study therapy 139
6.1.4. Overdose 140
6.2. Concomitant therapy, medications allowed and prohibited by the Protocol 140
6.2.1. Allowed concomitant therapy 140
6.2.2. Prohibited concomitant therapy 141
6.3. Compliance 141
7. Efficacy evaluation 142
7.1. List of efficacy variables 142
7.1.1. Definition of variables 142
7.1.2. Efficacy endpoints 142
7.1.3. Justification of the efficacy endpoints 143
7.2. Methods and timeframes for assessment, documenting, and analysis of efficacy variables 144
7.2.1 Timeframes for analysis of efficacy variables 144
7.2.2 Methods and timeframes for assessment and documenting of efficacy variables 144
8. Safety evaluation 145
8.1. List of safety variables 145
8.1.1. Terms and Definitions 145
8.1.2. Safety endpoints 146
8.2. Methods and timeframes for assessment, documenting and analysis of safety variables 147
8.2.1. Timeframes for analysis of safety variables 147
8.2.2. Methods and timeframes for assessment and documenting of safety variables 147
8.3. Requirements for reports, procedures for registration and reporting AEs, and filling out AE Report Forms 148
8.3.1. Documenting and Reporting AEs/SAEs 148
8.3.2. AE/SAE reporting 148
8.4. Methods and duration of follow-up for study subjects after the onset of AE/SAE 151
8.5. Immunogenicity study 152
9. Statistics 153
9.1. Description of statistical methods 153
9.2. Statistical analysis steps and timelines for reports 155
9.3. Planned number of subjects. Justification of sample size, including reasoning or calculations to justify statistical power, and clinical justification of the study 155

9.4. Suitable significance level 159
9.5. Statistical criteria for stopping and/or terminating the study 159
9.6. Handling of missing, unevaluable or uncertain data 159
9.7. Reporting any deviations from the initial statistical plan 159
9.8. Selection of subjects for analysis 160
10. Direct access to source data/documents 161
11. Quality control and quality assurance 162
11.1. Data quality assurance 162
11.2. Investigator's adherence to the Protocol 162
11.3. Investigator's responsibility to comply with the Protocol 162
11.4. Study monitoring 163
11.5. Data management and quality control 163
11.6. Study termination 164
12. Ethics 165
12.1. Ethical aspects of the study 165
12.2. Confidentiality of study subjects 165
13. Data handling and record keeping 166
13.1. Record keeping at the study site 166
13.2. Confidentiality of data 166
13.3. Collection of data 167
14. Finance and insurance 168
15. Publications 170
16. Appendices 171
Appendix 1. Dermatology Life Quality Index, DLQI 171
Appendix 2. SF-36 questionnaire 172
Appendix 3. Static Physicians Global Assessment (sPGA) 174
Appendix 4. Injection Site Reaction Form 175

List of tables

Table 1. Document history 17
Table 2. Names/positions of investigators responsible for the study conduct. Contact information of the study sites 18
Table 3. Names and addresses of clinical or other medical and/or technical services and/or organizations involved in the study 22
Table 4. [redacted] 41
Table 5. [redacted] 45
Table 6. [redacted] 47
Table 7. [redacted] 48
Table 8. [redacted] 52
Table 9. [redacted] 53
Table 10. [redacted] 55
Table 11. [redacted] 56
Table 12. [redacted] 57
Table 13. [redacted] 57
Table 14. Efficacy results from adalimumab studies in patients with moderate-to-severe plaque psoriasis (n – number of patients who responded to treatment; N – total number of patients in the treatment arm) 61
Table 15. [redacted] 83
Table 16. [redacted] 84
Table 17. [redacted] 84
Table 18. Study visits and procedures 91
Table 19. Clinical and laboratory examinations in the study 104
Table 20. Volume of blood to be taken from each subject in the study 108

Table 21. Psoriasis area and severity index (PASI). Lesion score: 0 - no symptoms; 1 – mild; 2 – moderate; 3 – severe; 4 – very severe 116
Table 22. Percentage area affected, % 117
Table 23. Nail psoriasis severity index (NAPSI). 118

List of figures

Figure 1. [redacted] 46
Figure 2. [redacted] 52
Figure 3. M03-656: study flow chart 60
Figure 4. Study flow-chart 80
Figure 5. An example of the visual analog scale for itch assessment 120

Signature page

To Protocol version 4.1 of March 05, 2018: An International Multicenter, Randomized, Double-Blind Study Comparing the Efficacy and Safety of BCD-057 (INN: Adalimumab, JSC BIOCAD, Russia) and Humira® (INN: Adalimumab, Vetter Pharma-Fertigung GmbH & Co. KG, Germany) in Patients with Moderate-to-Severe Plaque Psoriasis.

I, the undersigned, agree with the following:

1. I have read the Protocol, I agree with all the provisions of the Protocol, and I will conduct the study as outlined in this Protocol, the ICH GCP, and applicable regulations of the participating countries.
2. I will ensure no deviations from the Protocol to take place without prior written agreement from the Sponsor and documented approval from local ethics committees of participating countries, except where necessary to eliminate an immediate hazard to the study participants.
3. I confirm that all staff members are appropriately qualified to conduct the study, the study site has all necessary equipment, and I, the study investigator, have sufficient time to conduct this study in accordance with the Protocol
4. I will take all due measures to ensure that all staff members involved in the study are informed about their obligations in accordance with this Protocol.
5. I agree to fully cooperate with audits and inspections conducted in accordance with the rules established by the Sponsor and the state regulatory authorities.
6. I understand that the text of this Protocol and all other materials and results of this study are confidential and proprietary to the Sponsor. I agree not to disclose any of this information to a third party unless required to do so by the law of the participating countries.

Principal Investigator:

Signature

Full Name

Date

[redacted]

Signature

Date

Abbreviations

AE	Adverse event
AlkPh	Alkaline phosphatase
ALT	Alanine aminotransferase
APTT	Activated partial thromboplastin time
AST	Aspartate aminotransferase
AUC	Area under the concentration vs. time curve
$AUC_{tau,ss}$	Area under the concentration-time curve during a dosing interval at steady state
BAbs	Binding antibodies
BB	Blood biochemistry
BCG	Bacillus Calmette-Guérin
BMI	Body mass index
BP	Blood pressure
BSA	Body surface area affected by psoriasis
Cl	Total clearance
$C_{max,ss}$	Maximum concentration at steady state
C_{min}	Minimum concentration at steady state in any tau interval
CNS	Central nervous system
CRF	Case Report Form
CTCAE	Common Toxicity Criteria for Adverse Events
C_{trough}	Measured concentration at the end of a dosing interval at steady state (taken directly before next administration)
DLQI	Dermatology Life Quality Index
ECG	Electrocardiography
ELISA	Enzyme-Linked Immunosorbent Assay
EMA	European Medicines Agency
ESR	Erythrocyte sedimentation rate
FDA	Food and Drug Administration
GCP	Good Clinical Practice
GGT	Gamma-glutamyl transferase
GI	Gastrointestinal tract
HBcor	Hepatitis B core antigen
HbsAg	Hepatitis B surface antigen
HBV	Hepatitis B virus
HCG	Human chorionic gonadotropin
HCV	Hepatitis C virus
HIV	Human immunodeficiency virus
HIV	Human Immunodeficiency Virus
IC	Informed consent
ICH	International Conference on Harmonization
Ig	Immunoglobulin
IG	Immunogenicity
IMU	Summary of Product Characteristics
INN	International non-proprietary name
ITT	Intention-to-treat population (all patients who were randomized in the study, whether they completed the study per protocol or discontinued it due to any reason)
IWRS	Interactive Web Response System
JSC	Joined Stock Company
K_{el}	Elimination rate constant
MAb	Monoclonal antibody
mITT	modified intention-to-treat population (all of the randomized patients who meet a certain modification criterion)
NAb	Neutralizing antibodies
NAPSI	Nail Psoriasis Severity Index
NYHA	New-York Heart Association
PASI	Psoriasis Area and Severity Index
PD	Pharmacodynamics

PGA	Physician's global assessment
PK	Pharmacokinetics
PP	Per protocol population – patients who have no major protocol deviations up to and including Week 16
PUVA	Psoralen + ultraviolet A
PVC	Polyvinyl chloride
RPR-test	Baseline non-treponemal test to antibodies (reagins) of IgG and IgM classes to lipoid and lipoprotein-like material released from damaged cells of a patient with syphilis
SAE	Serious adverse event
SD	Standard deviation
SF-36	36-Item Short Form Health Survey
sPGA	Static physician's global assessment
T½	Half-life period
TNF	Tumor necrosis factor
UADR	Unexpected adverse drug reaction
UV	Ultraviolet light
Vd	Volume of distribution
$C_{av\ ss}$	Average steady-state plasma drug concentration
T_{max}	Time to maximum concentration

Terms and definitions

Term	Definition
Investigational product	This term includes the test drug and the reference drug. It is a pharmaceutical form of an active ingredient being tested or used as a reference in a clinical trial, including a product with a marketing authorization when used or assembled (formulated or packaged) in a way different from the approved form, or when used for an unapproved indication, or when used to gain further information about an approved use
Study/test product/drug	A pharmaceutical forms the properties of which are investigated in this clinical trial.
Comparator/reference product/drug	An active control being tested as a control in the clinical study to reduce the bias of assessments, keep the study therapy blind, and assess the internal validity of the study and/or comparative effects of the study product.
Case Report Form (CRF)	A printed or electronic document designed to record all the protocol-required information to be reported to the Sponsor on each study subject.
Investigator's Brochure	A compilation of the clinical and non-clinical information on the investigational product(s) that is relevant to the study of the investigational product(s) in human subjects.
Subject identification code / subject ID |__|__|-|__|__|__|	A unique identifier assigned by the investigator to each trial subject to protect the subject's identity and used in lieu of the subject's name when the investigator reports adverse events and/or other trial-related data.<br>Usually, the subject ID is a five-digit code where the first two digits are the site number and the last three digits are assigned sequentially to each participant as he/she enters the study.
Screening number |__|__|-|__|__|__|	A unique code assigned to each study subject who have signed the informed consent. The first two digits are the site number, and the last three digits are assigned sequentially to each patient as he/she gets enrolled at this particular study site.
Randomization number	A unique number assigned to each patient included to the study (randomized) and coding a specified therapy. After randomization, this number is not used anywhere.
Assessment	Procedure of obtaining the data that have to be gathered in this trial.
Inclusion in the study	The time point corresponding to randomization and assigning to therapy arms.
Any other therapy during the study	Any medications that study subjects receive as part of study procedures but that are not included in the study therapy. This includes, for example, the drugs used as part of the combination therapy.
Early discontinuation / withdrawal	The time point when the patient continues participation in the study before the planned investigational treatment is completed and/or assessments are performed. No further assessments are performed

Term	Definition
	beyond this point except for monitoring of the survival and/or disease progression in certain trials. If the patient discontinues the study due to an event planned by the Protocol (for example, a complete response), he/she is considered a dropout anyway.
Completion of the study	The time point when the patient attends his/her last study visit.
Study therapy	Any medication or a combination of medications used in any therapy group as part of study procedures, including concomitant medications and introductory therapy before the active medication.
Concomitant therapy	Therapy with any drugs included in the study therapy, except for the test drug and reference drug. For example, drugs used for combination therapy, pre-medication etc.
Discontinuation of study therapy	The time point corresponding to the permanent discontinuation of the study therapy regardless of the reason. It can correspond (or not) to the time point of the premature patient’s withdrawal
Variable	An identifier used for the data analysis and derived directly or indirectly from the protocol-specified assessments at pre-determined time points.

Document history

Table 1. Document history

Document	Version date	Narrative of changes
Version 1.0	December 21, 2015	N/A
Version 2.0 (Amendment 1)	November 29, 2016	Clarifications and additions to the text. Editorial corrections. Supersedes Protocol Version 1.0 of December 21, 2015.
Version 3.0	February 08, 2017	Adds a treatment crossover at Week 25 for patients in the reference arm. Editorial corrections. Supersedes Protocol Version 2.0 of November 29, 2016.
Version 4.0	September 18, 2017	Adds the T-SPOT-TB test to the list of procedures for tuberculosis diagnostics. Editorial corrections. Supersedes Protocol Version 3.0 of February 08, 2017.
Version 4.1	March 05, 2018	An additional analysis of the data obtained after 33 treatment weeks was introduced; a section related to clinical trials of BCD-057 was updated. Supersedes Protocol Version 4.0 of September 18, 2017.

Names/positions of investigators responsible for the study conduct. Contact information of the study sites

Table 2. Names/positions of investigators responsible for the study conduct. Contact information of the study sites

[redacted]	[redacted]	[redacted]	[redacted]
[redacted]			
[redacted]	[redacted]	[redacted]	[redacted]
[redacted]	[redacted]	[redacted]	[redacted]
[redacted]	[redacted]	[redacted]	[redacted]
[redacted]	[redacted]	[redacted]	[redacted]
[redacted]	[redacted]	[redacted]	[redacted]
[redacted]	[redacted]	[redacted]	[redacted]
[redacted]	[redacted]	[redacted]	[redacted]
[redacted]	[redacted]	[redacted]	[redacted]
[redacted]	[redacted]	[redacted]	[redacted]
[redacted]	[redacted]	[redacted]	[redacted]
[redacted]	[redacted]	[redacted]	[redacted]

[redacted]	[redacted]	[redacted]	[redacted]
[redacted]	[redacted]	[redacted]	[redacted]
[redacted]	[redacted]	[redacted]	[redacted]
[redacted]	[redacted]	[redacted]	[redacted]
[redacted]	[redacted]	[redacted]	[redacted]
[redacted]	[redacted]	[redacted]	[redacted]
[redacted]	[redacted]	[redacted]	[redacted]
[redacted]	[redacted]	[redacted]	[redacted]
[redacted]	[redacted]	[redacted]	[redacted]
[redacted]	[redacted]	[redacted]	[redacted]
[redacted]	[redacted]	[redacted]	[redacted]
[redacted]	[redacted]	[redacted]	[redacted]
[redacted]	[redacted]	[redacted]	[redacted]
[redacted]	[redacted]	[redacted]	[redacted]
[redacted]	[redacted]	[redacted]	[redacted]
[redacted]	[redacted]	[redacted]	[redacted]
[redacted]	[redacted]	[redacted]	[redacted]
[redacted]	[redacted]	[redacted]	[redacted]
[redacted]	[redacted]	[redacted]	[redacted]

[redacted]	[redacted]	[redacted]	[redacted]
[redacted]	[redacted]	[redacted]	[redacted]
[redacted]	[redacted]	[redacted]	[redacted]
[redacted]	[redacted]	[redacted]	[redacted]
[redacted]	[redacted]	[redacted]	[redacted]
[redacted]	[redacted]	[redacted]	[redacted]
[redacted]	[redacted]	[redacted]	[redacted]
[redacted]	[redacted]	[redacted]	[redacted]
[redacted]	[redacted]	[redacted]	[redacted]
[redacted]	[redacted]	[redacted]	[redacted]
[redacted]	[redacted]	[redacted]	[redacted]
[redacted]	[redacted]	[redacted]	[redacted]
[redacted]	[redacted]	[redacted]	[redacted]
[redacted]			
[redacted]	[redacted]	[redacted]	[redacted]
[redacted]	[redacted]	[redacted]	[redacted]
[redacted]	[redacted]	[redacted]	[redacted]

Name, job title, address and telephone number of the qualified physician responsible for taking medical decisions

Not applicable.

Names and addresses of clinical or other medical and/or technical services and/or organizations involved in the study

Table 3. Names and addresses of clinical or other medical and/or technical services and/or organizations involved in the study

[redacted]	[redacted]	[redacted]	[redacted]	[redacted]	[redacted]
[redacted]	[redacted]	[redacted]	[redacted]	[redacted]	[redacted]
[redacted]	[redacted]	[redacted]	[redacted]	[redacted]	[redacted]
[redacted]	[redacted]	[redacted]	[redacted]	[redacted]	[redacted]

SYNOPSIS	
**Protocol ID:**	BCD-057-2
**Study Title**	An International Multicenter, Randomized, Double-Blind Study Comparing the Efficacy and Safety of BCD-057 (INN: Adalimumab, JSC BIOCAD, Russia) and Humira® (INN: Adalimumab, Vetter Pharma-Fertigung GmbH & Co. KG, Germany) in Patients with Moderate-to-Severe Plaque Psoriasis
**Study Phase:**	III
**Study Sponsor:**	JSC BIOCAD<br>Mailing address: Petrovo-Dalneye, Krasnogorskiy District, Moscow Region, Russian Federation, 143422<br>Legal address: 34A, Svyazi Ul., Strelna, Petrodvortsoviy District, St. Petersburg, Russian Federation, 198515
**Test Drug**	BCD-057 (INN: adalimumab, JSC BIOCAD, Russia), solution for subcutaneous injection, 40 mg.
**Reference Drug**	Humira® (INN: adalimumab), Vetter Pharma-Fertigung Gmbh & Co. KG, Germany, solution for subcutaneous injection, 40 mg.
**Study Objectives and Goals:**	**Primary study objective:**<br>• To investigate the efficacy and safety of BCD-057 versus Humira® in patients with moderate-to-severe plaque psoriasis diagnosed at least 6 months before signing the informed consent form.<br>**Secondary objectives:**<br>1. To compare the efficacy of BCD-057 and Humira® by evaluating the proportion of patients who achieve a PASI 75 response, the changes in the affected body surface area (BSA).<br>2. To study the steady-state pharmacokinetics of BCD-057 and Humira® in a limited population.<br>3. To assess the safety and immunogenicity of BCD-057 versus Humira®.<br>Study goals:<br>1. To determine the proportion of patients achieving PASI 75 response at Week 16, by study arms.<br>2. To determine the proportion of patients achieving PASI 75 response at weeks 24, 33, and 55, by study arms.<br>3. To determine the proportion of patients achieving PASI 50/90 response at weeks 16, 24, 33, and 55, by study arms.<br>4. To assess changes from baseline in PASI score (%) at weeks 16, 24, 33, and 55, by study arms.<br>5. To determine the proportion of patients achieving a reduction in the sPGA score to “complete clearance” (score 0) or “minimal disease” (score 1) at weeks 16, 24, 33, and 55, by study arms.

SYNOPSIS	
	6. To assess changes from baseline in the affected body surface area (BSA) at weeks 16, 24, 33, and 55, by study arms.<br>7. To evaluate the improvement from baseline in the nail psoriasis severity index (NAPSI) at weeks 16, 24, 33, and 55, by study arms.<br>8. To assess change from baseline in the itch severity (VAS score from 0 to 100 mm) at weeks 16, 24, 33, and 55, by study arms.<br>9. To determine the PK parameters ($AUC_{tau,ss}$, $C_{av,ss}$, $C_{max,ss}$, $C_{min,ss}$, and C_{trough}) of adalimumab at steady state. Limited population (180 patients total, which includes 90 patients from the BCD-057 arm and 90 patients from the Humira® arm).<br>10. To assess changes from baseline in the quality of life (DLQI and SF-36) at weeks 16, 24, 33, and 55, by study arms.<br>11. To determine the proportion of patients developing binding/neutralizing anti-adalimumab antibodies, by study arms. To determine whether there is a correlation between the presence of neutralizing antibodies and the therapeutic efficacy of BCD-057/Humira®.<br>12. To determine the proportion of patients with treatment-emerging AEs/SAEs, by study arms.
**Study Design**	This clinical study of the efficacy and safety of BCD-057 and Humira® in patients with moderate-to-severe plaque psoriasis is an international, multicenter, double-blind, randomized parallel-group study with an active comparator (Phase III).<br>The study will include 344 patients with definite moderate-to-severe plaque psoriasis diagnosed at least 6 months before signing the informed consent (the diagnosis should be confirmed by source documents). The patients should have the BSA of at least 10%, the PASI score of at least 12, and the sPGA score of at least 3.<br>**Randomization**<br>Before enrollment, all patients will be given the full information about this clinical study, its purpose and the risks associated with study participation. After signing the informed consent form, the patient will undergo a screening examination (for not more than 4 weeks), which aims at confirming that the patient meets the study eligibility criteria. The Protocol does not provide any additional requirements regarding the diet or physical activity during the screening or study period.<br>According to results of the screening exam, the investigator will make a decision whether to include the patient in the study. After the investigator decides that the patient can take part in the study, the patient will be stratified according to his/her body weight (<80 kg / ≥81 kg), prior use of monoclonal antibodies for the treatment of

SYNOPSIS	
	psoriasis[1] (experienced / naive), PASI score (< 20 / ≥ 20), and symptoms of psoriatic arthritis (yes / no) and will be randomly assigned at a 1:1 frequency to one of the two study arms:<br>• Patients in Arm 1 (n = 172) will receive BCD-057 as SC injections given according to the following regimen: 80 mg on Day 1 of Week 0 followed by 40 mg on Day 1 of weeks 1, 3, 5, 7, 9, 11, 13, 15, 17, 19, 21, and 23.<br>• Patients in Arm 2 (n = 172) will receive Humira® as SC injections given according to the following regimen: 80 mg on Day 1 of Week 0 followed by 40 mg on Day 1 of weeks 1, 3, 5, 7, 9, 11, 13, 15, 17, 19, 21, and 23.<br>To evaluate the effects of switching from the originator to the biosimilar, patients will be re-randomized at Week 24. As a result of this second randomization, patients from the Humira® arm will be assigned at a 1:1 frequency to one of the two sub-arms.<br>• Patients in sub-Arm 1 will stay on Humira® 40 mg and receive injections on Day 1 of weeks 25, 27, 29, 31, 33, 35, 37, 39, 41, 43, 45, 47, 49, and 51.<br>• Patients in sub-Arm 2 will receive injections of BCD-057 40 mg on Day 1 of weeks 25, 27, 29, 31, 33, 35, 37, 39, 41, 43, 45, 47, 49, and 51.<br>To maintain the double-blind study design, patients from the BCD-057 arm will also be invited for re-randomization. However, in this case, this will be just a nominal procedure where patients will be assigned new randomization IDs and lot numbers. They will continue BCD-057 according to the following regimen: 40 mg on Day 1 of weeks 25, 27, 29, 31, 33, 35, 37, 39, 41, 43, 45, 47, 49, and 51.<br>Regardless of the treatment arm to which they are assigned, patients will be followed up until Week 55 (if the patient is not earlier removed from the study).<br>Patients withdrawn from the study due to safety reasons (AE/SAE) will not be replaced. Patients who discontinued the study due to other reasons can be replaced until Week 16 only by the decision of the Sponsor.<br>The pharmacokinetics of adalimumab will be investigated in a limited patient population, which includes 90 patients from each study arm (180 subjects total).<br>This study comprises the screening period and 30 visits (for all patients) or 39 visits (for patients involved in the PK study).<br><br>Study periods

[1] Except for therapeutic monoclonal antibodies or their fragments that are specific for tumor necrosis factor alpha.

SYNOPSIS	
	The study will include the following periods:<br>**Screening:**<br>Up to 4 weeks (before randomization and inclusion in the study).<br>**Main Treatment Period:**<br>24 weeks (weeks 0 to 24).<br>**Crossover Period:**<br>Weeks 25 to 51.<br>**Follow-Up Period:**<br>Until Week 55 after the first injection of BCD-057/Humira® (i.e. 28 days after the last injection of BCD-057/Humira®).
**Study Population**	Male and female patients from 18 to 75 years old with definite moderate-to-severe plaque psoriasis diagnosed at least 6 months before signing the informed consent. This study does not involve patients who have already received adalimumab or any other TNF alpha blockers and patients who have already received two or more biologics for the treatment of psoriasis.
**Planned Sample Size**	344 patients
**Inclusion Criteria**	1. The patient signed the informed consent form.<br>2. The patient is 18 to 75 years old at screening.<br>3. The patient has moderate-to-severe plaque psoriasis and was diagnosed at least 6 months before signing the informed consent form.<br>4. The patient received at least one course of phototherapy or systemic therapy for psoriasis and, in the opinion of the investigator, responded inadequately, or the patient is a candidate for any of these therapies.<br>5. The BSA ≥ 10%, PASI ≥ 12, and sPGA ≥ 3 at screening.<br>6. Laboratory values at screening:<br>• Hb ≥ 10 g/dL (100 g/L)<br>• WBC count ≥ 3000/µL (3.0 × 10^9/L)<br>• Platelet count ≥ 100 000/µL (100×10^9/L)<br>• Neutrophil count ≥ 2000/µL (2 × 10^9/L)<br>• AST, ALT, and alkaline phosphatase ≤ 2.5 × ULN<br>• Serum creatinine < 176.8 µmol/L (2.0 mg/dL)

SYNOPSIS	
	7. The patient tested negative for serum and virologic markers of active and latent hepatitis B (HBV)[2] and hepatitis C (HCV)[3].<br>8. The female patient has a negative urine pregnancy test at screening (the test is not performed for women who are post-menopausal for at least 2 years or those who underwent surgical sterilization).<br>9. The patient tested negative for tuberculosis:<br>• The negative result of the QuantiFERON or<br>• T-Spot.TB.<br>OR<br>• The negative result of the Diaskintest®.<br>10. Patients with indeterminant and/or positive results of the QuantiFERON /T-Spot.TB / Diaskintest® are allowed in the study only if all of the following three criteria are met:<br>• The patient tested negative in one additional QuantiFERON or T-Spot.TB assay.<br>• No signs of active tuberculosis are seen on the chest X-ray (or fluorography or CT or MRI) done any time within 1 month before signing the informed consent form.<br>• The TB specialist reported that the patient has no tuberculosis. The report has to be obtained during the screening.<br>11. The investigator considers the patient able to carry out all the procedures per protocol.<br>12. The patient and his/her sexual partner with retained reproductive potential are ready to implement reliable contraception throughout the screening period (starting from signing the informed consent) and during four weeks after the last dose of BCD-057/Humira®. This requirement does not apply to the patients who had undergone surgical sterilization. Reliable contraception means one barrier method in combination with one of the following: spermicides, intrauterine device and/or oral contraceptives used by the patient or patient's partner.

[2] The screening examination includes tests for HBsAg and total antibodies to HBcor antigen (IgG+IgM). If the patient tested negative for these markers (HBsAg and anti-HBcor total), he/she is considered eligible for the study with respect to this criterion. If the patient tested positive for HBsAg, the patient cannot be included in the study regardless of the results for anti-HBcor. If the patient tested negative for HBsAg but positive for anti-HBcore-total, the patient has to undergo additional examinations. Additional examinations include but were not limited to the following: qualitative PCR for HBV DNA, anti-HBcor-IgG, anti-HBcor-IgM, blood biochemistry, and a consultation with an infectious disease specialist. Having considered the examinations and test results, the Sponsor decides whether to approve such a patient for the study.

[3] The screening examination includes a test for anti-HCV antibodies in the blood. If the patient tested negative for anti-HCV, the he/she is considered eligible for the study with respect to this criterion. If the patient tested positive for anti-HCV, the patient has to undergo a further examination. Additional examinations include but were not limited to the following: qualitative PCR for HCV RNA, blood biochemistry, and consultation with an infectious disease specialist. Having considered the examinations and test results, the Sponsor decides whether to approve such a patient for the study.

SYNOPSIS	
**Exclusion Criteria**	1. Baseline erythrodermic or pustular psoriasis or any other skin diseases (e.g. eczema) that can affect and/or complicate the assessment of psoriasis treatment with the study drugs.<br>2. The patient had been exposed to any monoclonal antibodies inhibiting TNFα.<br>3. The patient had used two or more monoclonal antibodies (or their fragments) against other targets.<br>4. Less than 12 weeks have passed between the patient stopped using the monoclonal antibody therapy and signing the informed consent form for this study.<br>5. The patient had used or now uses any of the following concomitant therapies:<br>• Systemic (oral or parenteral) glucocorticoids or systemic retinoids (acitretin) within 4 weeks before the date of signing the informed consent form or during the screening period.<br>• Systemic non-biologic medications including (but not limited to) methotrexate, sulphasalazine, cyclosporine, mycophenolate mofetil, apremilast, etc. within 4 weeks before signing the informed consent form; OR leflunomide or cyclosporine within 6 months before signing the informed consent form.<br>• Phototherapy [including selective phototherapy (UVB) and photochemotherapy (PUVA)] within 4 weeks before signing the informed consent form.<br>6. Vaccination with live or attenuated vaccines any time within 8 weeks before signing the informed consent form.<br>7. The patient had a major surgery[4] within 30 days before signing the informed consent form or a major surgery is planned during the study.<br>8. The patient has an active infection at screening or has a history of any infections meeting the criteria below:<br>• Any active infection that required systemic anti-infectious agents within 28 days before signing the informed consent form.<br>• Any infection that required hospitalization or intravenous and/or intramuscular administration of anti-infectious agents within 8 weeks before signing the informed consent form.<br>• Recurring, chromic or any other active infection if the investigator believes that the study drug can harm the patient.

[4] A surgery associated with a high risk of severe blood loss (> 10% of the circulating blood volume) or any other life-threatening event.

SYNOPSIS	
	9. Documented HIV infection or a history of a severe immunodeficiency of any origin.<br>10. HBs antigen, antibodies to HBcor[5] antigen and/or antibodies to hepatitis C virus[6] revealed at screening.<br>11. Ongoing tuberculosis or a history of tuberculosis.<br>12. A positive microprecipitation reaction (MPR) together with a positive *T. pallidum* hemagglutination assay (TPHA)[7]) at screening. If results of any of the two assays are positive, the patient has to undergo a further examination. In the case of a positive *T. pallidum* hemagglutination test and negative microprecipitation test, further examinations include as follows:<br>• If the patient has a history of syphilis, appropriate medical records should be provided confirming the adequate therapy for syphilis.<br>• ELISA [$ELISA_{total}$ or ELISA(IgG)+ELISA(IgM)], or immunofluorescence reaction with absorption, or *T. pallidum* immobilization test.<br>• The dermatology/venereology specialist confirms that the patient has or has no syphilis.<br>• If the *T. pallidum* hemagglutination test is negative and the microprecipitation test is positive, further examinations include as follows:<br>• Repeated microprecipitation (or VDRL) and a specific assay should be performed: immunofluorescence assay or ELISA ($ELISA_{total}$ or ELISA(IgG)+ELISA (IgM)).<br>• The dermatology/venereology specialist confirms that the patient has or has no syphilis.<br>• Having considered the report from the dermatology/venereology specialist and the results of additional examinations, the Sponsor decides whether to include this patient in the study.<br>13. Baseline concomitant medical conditions that can increase the risk of adverse events during the study therapy, affect the

[5] The screening examination includes tests for HBsAg and total antibodies to HBcor antigen (IgG+IgM). If the patient tested negative for these markers (HBsAg and anti-HBcor total), he/she is considered eligible for the study with respect to this criterion. If the patient tested positive for HBsAg, the patient cannot be included in the study regardless of the results for anti-HBcor. If the patient tested negative for HBsAg but positive for anti-HBcore-total, the patient has to undergo additional examinations. Additional examinations include but are not limited to the following: qualitative PCR for HBV DNA, anti-HBcor-IgG, anti-HBcor-IgM, blood biochemistry, and a consultation with an infectious disease specialist. Having considered the examinations and test results, the Sponsor decides whether to approve such a patient for the study.

[6] The screening examination includes a test for anti-HCV antibodies in the blood. If the patient tested negative for anti-HCV, the he/she is considered eligible for the study with respect to this criterion. If the patient tested positive for anti-HCV, the patient has to undergo a further examination. Additional examinations include but were not limited to the following: qualitative PCR for HCV RNA, blood biochemistry, and consultation with an infectious disease specialist. Having considered the examinations and test results, the Sponsor decides whether to approve such a patient for the study.

[7] If hemagglutination test cannot be performed, ELISA total or ELISA(IgG)+ELISA(IgM) should be done. In the case of positive results, the algorithm is the same as after a positive hemagglutination assay.

SYNOPSIS	
	assessment of the severity of psoriasis, mask, aggravate, or alter the symptoms of psoriasis, or result in the same clinical and/or laboratory and/or instrumental symptoms as those of psoriasis:<br>• Acute inflammatory diseases or relapses of chronic inflammatory diseases other than psoriasis.<br>• Stable angina class III-IV, unstable angina or a history of myocardial infarction within 1 year before signing the informed consent.<br>• Moderate to severe cardiac failure (NYHA classes III and IV).<br>• Severe treatment-resistant hypertension[8];<br>• Atopic asthma and/or a history of angioedema.<br>• Moderate to severe respiratory failure and/or COPD grade 3/4.<br>• Decompensated diabetes mellitus.<br>• Systemic autoimmune diseases (including systemic lupus erythematosus, Crohn's disease, ulcerative colitis, systemic scleroderma, inflammatory myopathy, mixed forms of inflammatory diseases of the connective tissue, overlap syndrome, etc.).<br>• Active neurological diseases, such as multiple sclerosis, Guillain-Barre syndrome, optic nerve neuritis, transverse myelitis, or a history of neurological symptoms suggesting demyelinating diseases of the central nervous system.<br>• Any other concurrent diseases (including but not limited to metabolism, blood, kidney, liver, lung, neurological, endocrine, heart, and gastrointestinal tract disorders and/or infections) that, in the investigator's opinion, may affect the course of psoriasis, confound the assessment of its symptoms, or put the study subject at unacceptable risk during the treatment with BCD-057/Humira®.<br>14. Malignancies in remission for less than 5 years, except for adequately treated (cured) squamous and basal cell carcinoma, cervical cancer *in situ* or ductal breast carcinoma *in situ*.<br>15. A history of hypersensitivity to adalimumab or any excipients of Humira® and/or BCD-057 (mannitol, citric acid monohydrate, sodium citrate, sodium hydrogen phosphate dihydrate, sodium dihydrogen phosphate dihydrate, sodium chloride, polysorbate 80, sodium hydroxide).

[8] Resistant hypertension includes all cases of hypertension that are not controlled by the concurrent use of three anti-hypertensive drugs of different classes, including a diuretic, and non-medication methods (salt-free diet, controlled physical exercise).

SYNOPSIS	
	16. Known allergy to monoclonal antibodies (murine, chimeric, humanized or fully human).<br>17. Pregnancy, breastfeeding or planning for pregnancy while participating in the study.<br>18. Any psychiatric disorders, including a history of major depression and/or suicidal thoughts that can, in the investigator's opinion, put the patient at risk or affect patient's ability to follow the study protocol.<br>19. Use of recreational drugs, alcohol and/or medications and/or psychoactive substance abuse problems.<br>20. Participation in any other clinical study within 3 months before screening or simultaneous participation in other clinical studies[9].<br>21. Patients who were randomized to this study and then discontinued the study due to any reasons (i.e. patients who met all other inclusion/exclusion criteria), cannot be re-enrolled in this study.
**Study Therapy**	Adalimumab will be injected to patients of both arms: 80 mg SC at Week 0 followed by 40 mg SC once every two weeks, starting from Week 1. The efficacy of this dosing regimen was established in large pivotal studies, the results of which supported the approval of adalimumab for the treatment of plaque psoriasis. This regimen is presented in the approved Russian Instruction for Medical Use (IMU) for Humira® (ЛС-002422).<br>Injections of BCD-057/Humira® will be given at the study sites by a responsible qualified member of the study team (nurse, doctor, etc.).<br>Throughout the entire study, the patients are not allowed to use phototherapy [including selective phototherapy (UVB) and photochemotherapy (PUVA)], systemic non-biological anti-inflammatory agents (methotrexate, sulfasalazine, chlorambucil, leflunomide, cyclosporine A, azathioprine, cyclophosphamide, cyclosporine, apremilast, mycophenolate mofetil, etc.), aromatic retinoids (acitretin), systemic glucocorticoids for the treatment of psoriasis (oral or parenteral)[10], genetically engineered biological medicinal products, and live or attenuated vaccines.<br>Patients can use topical moisturizers, emollients, oils, salicylic acid products, topical antibacterial and antimycotic agents, and topical glucocorticoids as needed. All patients must discontinue all topical

[9] Patients whose screening was interrupted due to force majeure circumstances (external events beyond the patient's and investigator's control) could be re-screened in this study.
[10] The Protocol allows single doses of systemic glucocorticoids to manage acute anaphylactic reactions, shock, or other life-threatening events that developed as adverse events in patients during the study.

SYNOPSIS	
	skin products (medications or cosmetics) 24 hours before the planned PASI assessment
**Study Procedures**	To check whether patients meet the study eligibility criteria (for screened patients) and to assess the treatment efficacy (for enrolled patients), the study team will perform a comprehensive medical examination within the timelines specified by the Protocol. The examination includes:<br>• Baseline clinical characteristics and medical history<br>• Physical examination, body weight at screening<br>• BP and wrist pulse<br>• ECG<br>• Chest X-ray in posterior-anterior projection and/or fluorography<br>• CBC<br>• Blood biochemistry<br>• Urinalysis<br>• Pregnancy test, for women only (the test is carried out using the urine HCG test strips) Pregnancy test is not required in female patients who were at least 2 years post-menopausal or had a uterus or ovary surgery that makes pregnancy impossible.<br>• Infection status [Diaskintest® / QuantiFERON / T-Spot.TB test, HIV Ag/Ab Combo, p24 antigen assay, HBs-antigen, antibodies to HBcor (IgG + IgM/ IgG/ IgM) and antibodies to HCV, qualitative PCR for HCV RNA and/or HBV DNA (only in the case of positive results of the antibody assay), report provided by an infectious diseases specialist (only in the case of detected antibodies to HBcor or HCV), report from a TB specialist (in the case of indeterminant or positive TB test results), microprecipitation and TPHA (if results are positive, proceed to TPI test, ELISA, VDRL, or FTA-ABS at the discretion of the investigator, and obtain a consultation with a venereologist).<br>• BSA calculation<br>• PASI assessment<br>• NAPSI assessment<br>• Itch intensity (VAS, 0 to 100 mm)<br>• Static Physicians Global Assessment (sPGA)<br>• DLQI and SF-36 scores<br>• For safety evaluation, the following will be closely monitored: all general disorders (including fever and flu-like symptoms), abnormal vital signs (BP and wrist pulse), infectious complications, abnormal laboratory values (CBC results: hemoglobin, RBC, platelets, WBC including changes in the

SYNOPSIS	
	WBC differential, and ESR; blood biochemistry: glucose, bilirubin total, ALT, AST, GGT, alkaline phosphatase, and creatinine; urinalysis), cardiac disorders (ECG), and pulmonary conditions (chest X-ray and/or fluorography)).<br>• Immunogenicity of adalimumab (occurrence of binding/neutralizing anti-adalimumab antibodies) will be assessed in both treatment arms regularly throughout the study (before dosing and at weeks 16, 33, and 55). The assay will be performed at the BIOCAD Central Laboratory.<br>Additional blood specimens for PK assessment will be collected from a limited population (not more than 90 patients in each arm; not more than 180 patients total).
**Total Study Duration**	The expected duration of the study is 30 months. This includes patient recruitment (up to 12 months), treatment period, follow-up period, and collection and statistical processing of results.<br>Each subject will participate in the study for up to 59 weeks (about 14 months), including 4 weeks of screening, 51 weeks of treatment, and 4 weeks of follow-up.
**Efficacy Assessment**	**Primary endpoint:**<br>• The proportion of patients with a 75% reduction from baseline in psoriasis area and severity (PASI 75 response) at Week 16.<br>**Secondary endpoints:**<br>• Changes from baseline in PASI (%) at weeks 16, 24, 33, and 55, by study arms.<br>• The proportion of patients with a 75% reduction from baseline in psoriasis area and severity (PASI 75 response) at weeks 24, 33, and 55.<br>• The proportion of patients with a 50% and 90% PASI reduction from baseline at weeks 16. 24, 33, and 55.<br>• The proportion of patients with sPGA score reduced to 0 (complete clearance) or 1 (minimal disease) at weeks 16, 24, 33, and 55.<br>• Changes from baseline in affected BSA (%) at weeks 16, 24, 33, and 55.<br>• Changes from baseline in NAPSI (%) at weeks 16, 24, 33, and 55.<br>• Change from baseline in the itch severity (VAS score from 0 to 100) at weeks 16, 24, 33, and 55.<br>• Changes from baseline in health-related quality of life (DLQI and SF-36 scores) at weeks 16, 24, 33, and 55.<br>• Methods used for efficacy assessment

SYNOPSIS	
	• The efficacy assessment will be based on psoriasis activity indicators and the patient's general health state:<br>– PASI score,<br>– sPGA score,<br>– BSA,<br>– NAPSI,<br>– Itch intensity VAS score (0-100 mm),<br>– Health-related quality of life (DLQI and SF-36).
**Safety Assessment**	Safety endpoints:<br>• The proportion of patients who developed AEs/SAEs that, in the investigator's opinion, are related to Humira® or BCD-057, by study arms.<br>• The proportion of patients who experienced grade 3/4 AEs (CTCAE v. 4.03) that, in the investigator's opinion, are related to Humira® or BCD-057.<br>• The proportion of patients who developed AEs potentially due to TNFα inhibitors, by study arms. Such AEs include infections, serious infections, opportunistic infections (infections caused by opportunistic pathogenic viruses or cellular organisms), tuberculosis, malignancies, demyelinating diseases, lupus-like syndrome, congestive heart failure, allergic reactions, injection site reactions, and CBC or blood biochemistry abnormalities.<br>• The proportion of patients who discontinued the study due to AEs/SAEs
**Immunogenicity Assessment**	Immunogenicity endpoints:<br>• The proportion of patients with binding or neutralizing anti-adalimumab antibodies.<br>• The proportion of patients with binding or neutralizing anti-adalimumab antibodies and inadequate therapeutic response (achieved PASI 50 or less).
**PK Assessment (includes only 90 subjects from each arm, 180 subjects total)**	**PK endpoints**<br>• $AUC_{tau,ss}$ after multiple SC injections of Humira® or BCD-057.<br>• $C_{av,ss}$, $C_{max,ss}$, $C_{min,ss}$, C_{trough} after multiple SC injections of Humira® or BCD-057.<br>**Methods of PK assessment:**<br>The PK is based on adalimumab concentrations in the serum. Blood specimens will be taken at the following time points:<br>1. PK0: 15 min ± 5 min before the 1st injection of adalimumab (Day 1 of Week 0).<br>2. PK1: 15 min ± 5 min before the 2nd injection of adalimumab (Day 1 of Week 1).

SYNOPSIS	
	3. PK2: 15 min ± 5 min before the 3rd injection of adalimumab (Day 1 of Week 3).<br>4. PK3: 15 min ± 5 min before the 4th injection of adalimumab (Day 1 of Week 5).<br>5. PK4: 15 min ± 5 min before the 5th injection of adalimumab (Day 1 of Week 7).<br>6. PK5: 15 min ± 5 min before the 6th injection of adalimumab (Day 1 of Week 9).<br>7. PK6: 15 min ± 5 min before the 7th injection of adalimumab (Day 1 of Week 11).<br>8. PK7: 15 min ± 5 min before the 8th injection of adalimumab (Day 1 of Week 13).<br>9. PK8: 15 min ± 5 min before the 9th injection of adalimumab (Day 1 of Week 15).<br>10. PK9: 3 h ± 15 min after the 9th injection of adalimumab (Day 1 of Week 15).<br>11. PK10: 12 h ± 30 min after the 9th injection of adalimumab (Day 1 of Week 15).<br>12. PK11: 24 h ± 30 min after the 9th injection of adalimumab (Day 2 of Week 15).<br>13. PK12: 48 h ± 90 min after the 9th injection of adalimumab (Day 3 of Week 15).<br>14. PK13: 72 h ± 120 min after the 9th injection of adalimumab (Day 4 of Week 15).<br>15. PK14: 96 h ± 120 min after the 9th injection of adalimumab (Day 5 of Week 15).<br>16. PK15: 120 h ± 120 min after the 9th injection of adalimumab (Day 6 of Week 15).<br>17. PK16: 144 h ± 120 min after the 9th injection of adalimumab (Day 7 of Week 15).<br>18. PK17: 192 h ± 120 min after the 9th injection of adalimumab (Day 2 of Week 16).<br>19. PK18: 240 h ± 120 min after the 9th injection of adalimumab (Day 4 of Week 16).<br>20. PK19: 288 h ± 120 min after the 9th injection of adalimumab (Day 6 of Week 16).<br>21. PK20: 15 min ± 5 min before the 10th injection of adalimumab (Day 1 of Week 17).<br>22. PK21: 15 min ± 5 min before the 11th injection of adalimumab (Day 1 of Week 19).<br>23. PK22: 15 min ± 5 min before the 12th injection of adalimumab (Day 1 of Week 21).<br>24. 24. PK23: 15 min ± 5 min before the 13th injection of adalimumab (Day 1 of Week 23).

SYNOPSIS	
**Statistical Analysis**	**Methods of analysis**<br>Statistical analysis will be performed with the Statistica 10.0 software. The normally distributed data will be analyzed using the dependent-sample *t*-test and ANOVA. The non-normally distributed data will be analyzed using the Mann-Whitney test and ANOVA. The regression analysis will be applied for quantitative efficacy data. Non-categorical data will be processed using the Fisher's exact test, Yates-corrected χ^2 test and Cochran-Mantel-Haenszel test.<br>**Determination of sample size**<br>The purpose of the study is to demonstrate that the efficacy of BIOCAD's adalimumab biosimilar (BCD-057) is equivalent to the efficacy of Humira® in patients with psoriasis. The number of subjects was determined with the formula for calculating the sample size in two parallel-group equivalence studies as described in the [Chow Shein-Chung, Shao Jun, Wang Hansheng "Sample Size Calculations in Clinical Research"].<br>Selection of populations for analysis<br>**Efficacy analysis**<br>To assess whether the study has achieved its objective and to evaluate the treatment efficacy, the analysis will be performed in two populations: the ITT (intent-to-treat) population (all randomized patients) and the PP (per protocol) population (includes subjects who have no major protocol deviations up to and including Week 16).<br>If the patient is withdrawn from the study due to safety-related reasons before the primary endpoint is assessed, he/she will be included in the efficacy analysis and considered a non-responder.<br>**Safety analysis**<br>The safety population will include all randomized patients (ITT population). In addition, the study report will contain data on serious adverse events reported during screening (if any).<br>**Immunogenicity analysis**<br>The immunogenicity analysis will include all patients who received at least one dose of BCD-057/Humira®. Patients whose serum samples taken on Day 1 of Week 0 and at least one serum sample taken on any subsequent visit (weeks 16, 33, and 55) are missing/lost/spoiled, will be removed from immunogenicity analysis.<br>**PK analysis**<br>The PK study will involve a limited population (90 patients from each study arm, 180 subjects total). The PK analysis will include all of these 180 patients who:<br>• Completed all visits for PK assessment starting from Day 1 of Week 15 to Day 6 of Week 16, except for those who missed more than two PK blood samplings during this period.

SYNOPSIS	
	• Provided a blood specimen for adalimumab assay on Day 1 of Week 0 (before the first injection of BCD-057/Humira®).<br>• Missed not more than one dose of adalimumab within the period from the study start to Week 15 inclusive (injections 1 to 9).<br>**Report preparation**<br>**Efficacy analysis using the primary endpoint**<br>The final report will contain results of data analysis for the primary efficacy and safety endpoints after 16 weeks of the study.<br>**Supplementary report No. 1**<br>First supplementary report will present analytical results for PK, efficacy, safety, and immunogenicity using data obtained after 33 weeks.<br>**Supplementary report No. 2**<br>Second supplementary report will present analytical results for efficacy, safety, and immunogenicity using data obtained from the entire study (55 weeks).

1. Study rationale

1.1. Introduction

1.1.1. Overview of epidemiology, pathogenesis, and treatment options

1.1.1.1. Epidemiology and significance of the disease

Psoriasis is a common skin disorder. In developed countries, an estimate prevalence of psoriasis is up to 3%. In Russia, about 100 000 new cases are registered per year, and the prevalence of psoriasis is about 2% to 3%.

Clinical signs of psoriasis may vary from mild forms manifesting as a few local rashes on the skin to severe forms (about 30% of cases) affecting a significant percentage of the body surface.

In the last decades, severe therapy-resistant forms of psoriasis have become more common. They significantly deteriorate the patients' quality of life and lead, in some cases, to disability. This determines the social significance of psoriasis. According to Krueger et al., psoriasis shares the first place with cardiovascular disorders and diabetes as a medical and a social problem and a as factor decreasing the quality of life.

Today, psoriasis is considered incurable. However, advanced treatment options are able to significantly improve the clinical course of the disease and make remissions longer.

1.1.1.2. Current treatment options

Psoriasis is commonly treated with topical agents such as glucocorticoids, calcipotriol, anthralin, coal tar extracts, etc. These treatment options are indicated to patients with mild psoriasis. Phototherapy is an important option in the treatment of psoriasis. There are three main types of phototherapy: photochemotherapy (PUVA), which is a combination of the UVA light and a photosensitizing agent taken orally; selective phototherapy, which is a combination of middle- and long-wave UV light; and the narrow-band UVB therapy. Therapeutic effect of the UV-light is likely due to stimulation of the production of cytokines with immunosuppressive effects, enhancement of the expression of several molecules on the cell surface, and induction of apoptosis.

Retinoids (synthetic vitamin A derivatives) have been used in psoriasis for more than 25 years. Retinoids inhibit the proliferation of epidermis, normalize keratinization, exert immunomodulating effects on dermal cells, and stabilize membrane structures of cells.

In the recent decades, opinions about mechanisms of psoriasis have changed, and now psoriasis is understood to be a systemic autoimmune disease. Thus, approaches to its therapy have been revised. Cytostatic agents (methotrexate) and systemic glucocorticoids are taking a backseat to agents that are more selective. Genetically engineered biologics became a major achievement in the

treatment of psoriasis patients. In particular, introducing tumor necrosis factor alpha (TNFα) blockers into clinical practice was a breakthrough in the therapy of psoriasis and changed the world opinion about its potential results.

One of the key priorities in this field is the development of monoclonal antibodies (MAbs) and their derivatives targeting specific molecules of immune cells and/or pro-inflammatory cytokines, recombinant anti-inflammatory cytokines, and natural cytokine inhibitors (soluble receptors or their antagonists). Etanercept was the first product of this class to be approved for the treatment of psoriasis. This is a fully human recombinant fusion protein consisting of two extra-cellular domains of the p75 TNFα receptor. The domains are linked together with the Fc of the IgG1. The next product of this class introduced in clinical practice was infliximab (Remicade, Schering-Plough), a chimeric anti-TNFα MAb. This drug binds TNFα with high affinity and specificity to give stable complexes and inhibits the biological effects of TNFα, which results in lysis (or apoptosis) of the TNF-expressing cells.

Adalimumab is a recombinant monoclonal antibody with the same peptide sequence as that of the human IgG1. Adalimumab selectively binds to TNFα and neutralizes its biological functions by blocking its interaction with the surface cellular p55- and p75 receptors.

Another MAb commonly used in psoriasis is ustekinumab, which has a different mechanism of action. Ustekinumab contains fully human MAbs against the p40 subunit of both IL-12 and IL-23. These cytokines play a key role in the pathogenesis of inflammation mediated by T helper cells 1 and 17.

Cyclosporine is another systemic agent used in clinical practice for the treatment of psoriasis. It is a cyclic polypeptide inhibiting the activity of T cells and reducing their antigen sensitivity.

Thus, psoriasis is a common and socially significant disease. Biological medicinal products that affect various aspects of the psoriasis pathogenesis are now the most promising therapeutic agents. These drugs show high efficacy and favorable safety profiles. Adalimumab is one of these drugs and it has shown good results in clinical practice.

1.1.2. Background information for studied therapy

Adalimumab (the original drug Humira®) has proved itself as a powerful treatment for moderate-to-severe plaque psoriasis. Adalimumab is a fully human recombinant monoclonal antibody with the amino acid sequence of its constant domains being similar to that of the human IgG1. Adalimumab selectively binds to tumor necrosis factor alpha (TNFα) and neutralizes its biological effects by preventing it from interacting with the cell surface receptors p55 and p75. TNFα is a multifunctional pro-inflammatory cytokine, a key element in the mechanisms underlying several

autoimmune diseases. TNFα was shown to be involved in connective tissue and musculoskeletal system disorders associated with autoimmune damage in the stroma of parenchymatous organs, in joints and surrounding tissues, and in perivascular areas of the blood vessels.

Multiple controlled clinical studies showed that adalimumab increases the efficacy of conventional therapy and has a favorable safety profile in rheumatoid arthritis, psoriatic arthritis, Crohn’s disease, ulcerative colitis, and psoriasis. Today, Humira® is marketed in the Russian Federation. However, it is very expensive, and not all patients can afford this medication. The Russian company BIOCAD has developed its own medication of adalimumab – BCD-057, a biosimilar of Humira®. Multiple physicochemical tests, *in vitro* and *in vivo* biological activity assays, PK and toxicity studies in laboratory animals, and a comparative Phase I study in healthy volunteers showed that BCD-057 and Humira® do not differ in their main characteristics or effects. Although, a large Phase III clinical study in the target population has to be conducted to demonstrate the equivalence of the biosimilar and the originator.

1.2. Name and description of investigational products

The investigational products in this study were BCD-057 (JSC BIOCAD, Russia) and Humira® (Vetter Pharma-Fertigung Gmbh & Co. KG, Germany).

Test drug:

Internal code: BCD-057

INN: adalimumab

Comparator

Invented name: Humira®

INN: adalimumab

The Russian biotechnology company BIOCAD has developed a biosimilar to Humira®. BIOCAD’s adalimumab (BCD-057) was tested in comprehensive physicochemical studies, *in vitro* and *in vivo biological activity assays*, and PK, PD, and toxicity studies in animals. All these studies demonstrated that BCD-057 is equivalent to Humira®.

To date, results of the Phase I study have been obtained. The single-dose PK of SC BCD-057 was shown to be equivalent to the single-dose PK of SC Humira®, and the safety profile of BCD-057 did not differ significantly from that of Humira®.

Results of this clinical study provided the grounds for a Phase III clinical study. This study aims at evaluating the efficacy, safety, immunogenicity, and PK of BCD-057 (JSC BIOCAD) in patients with moderate-to-severe plaque psoriasis who have already received phototherapy or systemic therapy or those being candidates for such therapies. Adalimumab may decrease the activity of

psoriasis, improve its clinical course, reduce body surface area affected by psoriasis, and improve patients' quality of life.

Introducing the Russian adalimumab biosimilar into clinical practice could make modern therapy with TNFα blockers much more available for patients including those with moderate-to-severe psoriasis.

1.3. Relevant non-clinical and clinical aspects

1.3.1. Non-clinical studies

[redacted]

[redacted]

[redacted]

Table 4. [redacted]

[redacted]	[redacted]	[redacted]	[redacted]	[redacted]
[redacted]	[redacted]	[redacted]	[redacted]	[redacted]
[redacted]	[redacted]			
[redacted]	[redacted]	[redacted]	[redacted]	[redacted]
		[redacted]	[redacted]	[redacted]
		[redacted]	[redacted]	[redacted]
		[redacted]	[redacted]	[redacted]
		[redacted]	[redacted]	[redacted]
		[redacted]	[redacted]	[redacted]
[redacted]	[redacted]	[redacted]	[redacted]	[redacted]
		[redacted]	[redacted]	[redacted]
		[redacted]	[redacted]	[redacted]

[redacted]	[redacted]	[redacted]	[redacted]	[redacted]
		[redacted]	[redacted]	[redacted]
		[redacted]	[redacted]	[redacted]
		[redacted]	[redacted]	[redacted]
		[redacted]	[redacted]	[redacted]
		[redacted]	[redacted]	[redacted]
[redacted]	[redacted]	[redacted]	[redacted]	[redacted]
		[redacted]	[redacted]	
[redacted]	[redacted]	[redacted]	[redacted]	[redacted]
[redacted]	[redacted]	[redacted]	[redacted]	[redacted]
[redacted]	[redacted]	[redacted]	[redacted]	[redacted]
		[redacted]	[redacted]	[redacted]
[redacted]	[redacted]	[redacted]	[redacted]	[redacted]
[redacted]	[redacted]	[redacted]	[redacted]	[redacted]
		[redacted]	[redacted]	[redacted]
		[redacted]	[redacted]	[redacted]
		[redacted]	[redacted]	[redacted]
[redacted]	[redacted]	[redacted]	[redacted]	[redacted]
[redacted]	[redacted]	[redacted]	[redacted]	[redacted]
		[redacted]	[redacted]	[redacted]
		[redacted]	[redacted]	[redacted]
		[redacted]	[redacted]	[redacted]
[redacted]	[redacted]	[redacted]	[redacted]	[redacted]
[redacted]	[redacted]	[redacted]	[redacted]	[redacted]
[redacted]	[redacted]	[redacted]	[redacted]	[redacted]
[redacted]	[redacted]	[redacted]	[redacted]	[redacted]
[redacted]	[redacted]	[redacted]	[redacted]	[redacted]
		[redacted]	[redacted]	[redacted]
[redacted]	[redacted]	[redacted]	[redacted]	[redacted]
[redacted]	[redacted]	[redacted]	[redacted]	[redacted]
[redacted]	[redacted]	[redacted]	[redacted]	[redacted]

[redacted]	[redacted]	[redacted]	[redacted]	[redacted]
		[redacted]	[redacted]	[redacted]
		[redacted]	[redacted]	[redacted]
[redacted]	[redacted]	[redacted]	[redacted]	[redacted]
[redacted]	[redacted]	[redacted]	[redacted]	[redacted]
[redacted]	[redacted]	[redacted]	[redacted]	[redacted]
[redacted]	[redacted]	[redacted]	[redacted]	[redacted]
[redacted]	[redacted]	[redacted]		[redacted]
[redacted]	[redacted]	[redacted]		[redacted]
[redacted]	[redacted]	[redacted]	[redacted]	[redacted]
[redacted]	[redacted]	[redacted]	[redacted]	[redacted]
[redacted]	[redacted]	[redacted]	[redacted]	[redacted]
[redacted]	[redacted]	[redacted]	[redacted]	[redacted]
[redacted]	[redacted]	[redacted]	[redacted]	[redacted]
		[redacted]	[redacted]	[redacted]
[redacted]	[redacted]	[redacted]	[redacted]	[redacted]
[redacted]	[redacted]	[redacted]	[redacted]	[redacted]
[redacted]	[redacted]	[redacted]	[redacted]	[redacted]
[redacted]	[redacted]	[redacted]	[redacted]	[redacted]
[redacted]	[redacted]	[redacted]	[redacted]	[redacted]
[redacted]	[redacted]	[redacted]	[redacted]	[redacted]

[redacted]

[redacted]

[redacted]

[redacted]

[redacted]

[redacted]

Table 5. [redacted]

[redacted]	[redacted]		[redacted]		[redacted]		[redacted]		[redacted]		[redacted]	
	[redacted]	[redacted]	[redacted]	[redacted]	[redacted]	[redacted]	[redacted]	[redacted]	[redacted]	[redacted]	[redacted]	[redacted]
[redacted]	[redacted]	[redacted]	[redacted]	[redacted]	[redacted]	[redacted]	[redacted]	[redacted]	[redacted]	[redacted]	[redacted]	[redacted]
[redacted]	[redacted]	[redacted]	[redacted]	[redacted]	[redacted]	[redacted]	[redacted]	[redacted]	[redacted]	[redacted]	[redacted]	[redacted]
[redacted]	[redacted]	[redacted]	[redacted]	[redacted]	[redacted]	[redacted]	[redacted]	[redacted]	[redacted]	[redacted]	[redacted]	[redacted]
[redacted]	[redacted]	[redacted]	[redacted]	[redacted]	[redacted]	[redacted]	[redacted]	[redacted]	[redacted]	[redacted]	[redacted]	[redacted]
[redacted]	[redacted]	[redacted]	[redacted]	[redacted]	[redacted]	[redacted]	[redacted]	[redacted]	[redacted]	[redacted]	[redacted]	[redacted]
[redacted]	[redacted]		[redacted]		[redacted]		[redacted]		[redacted]		[redacted]	
[redacted]												

[redacted]

[redacted]

[redacted]

Figure 1. [redacted]

[redacted]

Table 6. [redacted]

	[redacted]		[redacted]		[redacted]		[redacted]		[redacted]		[redacted]	
[redacted]	[redacted]	[redacted]	[redacted]	[redacted]	[redacted]	[redacted]	[redacted]	[redacted]	[redacted]	[redacted]	[redacted]	[redacted]
[redacted]	[redacted]	[redacted]	[redacted]	[redacted]	[redacted]	[redacted]	[redacted]	[redacted]	[redacted]	[redacted]	[redacted]	[redacted]
[redacted]	[redacted]	[redacted]	[redacted]	[redacted]	[redacted]	[redacted]	[redacted]	[redacted]	[redacted]	[redacted]	[redacted]	[redacted]
[redacted]	[redacted]	[redacted]	[redacted]	[redacted]	[redacted]	[redacted]	[redacted]	[redacted]	[redacted]	[redacted]	[redacted]	[redacted]
[redacted]	[redacted]	[redacted]	[redacted]	[redacted]	[redacted]	[redacted]	[redacted]	[redacted]	[redacted]	[redacted]	[redacted]	[redacted]
[redacted]	[redacted]	[redacted]	[redacted]	[redacted]	[redacted]	[redacted]	[redacted]	[redacted]	[redacted]	[redacted]	[redacted]	[redacted]
[redacted]	[redacted]		[redacted]		[redacted]		[redacted]		[redacted]		[redacted]	
[redacted]												

[redacted]

[redacted]

Table 7. [redacted]

[redacted]	[redacted]	
	[redacted]	[redacted]
[redacted]	[redacted]	[redacted]
[redacted]	[redacted]	[redacted]
[redacted]	[redacted]	[redacted]
[redacted]	[redacted]	[redacted]
[redacted]	[redacted]	[redacted]

[redacted]	[redacted]	
	[redacted]	[redacted]
[redacted]	[redacted]	[redacted]
[redacted]	[redacted]	[redacted]
[redacted]	[redacted]	

[redacted]

1.3.2. Clinical trials

BCD-057 is an adalimumab biosimilar, which has the same active ingredient and dosage as the original drug Humira®. To date, BIOCAD has completed a single-center, double-blind, comparative, randomized clinical study of the PK, tolerability, and safety of BCD-057 (JSC BIOCAD, Russia) versus Humira® after a single SC administration in healthy volunteers. The study involved 94 healthy volunteers and has demonstrated the equivalent characteristics of the drugs. Results of this study are presented below. In addition, this section presents results from key Humira® clinical studies, which showed the high efficacy and safety of adalimumab in patients with moderate-to-severe plaque psoriasis.

1.3.2.1. Comparative single-dose study of pharmacokinetics, safety, and tolerability

The safety, PK, and immunogenicity of BCD-057 after a single SC administration were studied in a single-center, double-blind, comparative, randomized clinical study of BCD-057 (JSC BIOCAD) versus Humira® in healthy volunteers.

[redacted]

[redacted]

[redacted]

[redacted]

[redacted]

Figure 2. [redacted]

[redacted]

Table 8. [redacted]

[redacted]	[redacted]	[redacted]
[redacted]	[redacted]	[redacted]
[redacted]	[redacted]	[redacted]
[redacted]	[redacted]	[redacted]

[redacted]

[redacted]

[redacted]

[redacted]

[redacted]

[redacted]

[redacted]

Table 9. [redacted]

[redacted]	[redacted]		[redacted]		[redacted] [redacted]
	[redacted] [redacted]	[redacted] [redacted]	[redacted] [redacted]	[redacted] [redacted]	
[redacted]					
[redacted]	[redacted] [redacted]	[redacted] [redacted]	[redacted] [redacted]	[redacted] [redacted]	[redacted] [redacted]
[redacted]	[redacted] [redacted]	[redacted] [redacted]	[redacted] [redacted]	[redacted] [redacted]	[redacted] [redacted]
[redacted]	[redacted] [redacted]	[redacted] [redacted]	[redacted] [redacted]	[redacted] [redacted]	[redacted] [redacted]
[redacted]	[redacted] [redacted]	[redacted] [redacted]	[redacted] [redacted]	[redacted] [redacted]	[redacted] [redacted]
[redacted]					
[redacted]	[redacted] [redacted]	[redacted] [redacted]	[redacted] [redacted]	[redacted] [redacted]	[redacted] [redacted]
[redacted]	[redacted] [redacted]	[redacted] [redacted]	[redacted] [redacted]	[redacted] [redacted]	[redacted] [redacted]

[redacted]	[redacted]		[redacted]		[redacted]
	[redacted]	[redacted]	[redacted]	[redacted]	
[redacted]	[redacted]	[redacted]	[redacted]	[redacted]	[redacted]
[redacted]					

[redacted]

[redacted]

[redacted]

[redacted]

[redacted]

[redacted]

[redacted]

[redacted]

Table 10. [redacted]

[redacted]	[redacted]				[redacted]
	[redacted]		[redacted]		
	[redacted]	[redacted]	[redacted]	[redacted]	
[redacted]	[redacted]	[redacted]	[redacted]	[redacted]	[redacted]
[redacted]	[redacted]	[redacted]	[redacted]	[redacted]	[redacted]
[redacted]					

[redacted]

[redacted] BCD-057 and Humira® showed equivalent PK, safety, tolerability and immunogenicity profiles after a single injection in healthy volunteers. Based on these findings, BCD-057 was recommended for further clinical studies.

[redacted]

[redacted]

[redacted]

[redacted]

Table 11. [redacted]

[redacted]	[redacted]				[redacted]
	[redacted]		[redacted]		
	[redacted]	[redacted]	[redacted]	[redacted]	
[redacted]					
[redacted]	[redacted]	[redacted]	[redacted]	[redacted]	[redacted]
[redacted]					
[redacted]	[redacted]	[redacted]	[redacted]	[redacted]	[redacted]
[redacted]	[redacted]	[redacted]	[redacted]	[redacted]	[redacted]
[redacted]	[redacted]	[redacted]	[redacted]	[redacted]	[redacted]

[redacted]

[redacted]

[redacted]

[redacted]

Table 12. [redacted]

[redacted]	[redacted]		
	[redacted]	[redacted]	[redacted]
[redacted]	[redacted]	[redacted]	[redacted]
[redacted]	[redacted]	[redacted]	[redacted]
[redacted]	[redacted]	[redacted]	[redacted]
[redacted]	[redacted]	[redacted]	[redacted]
[redacted]	[redacted]	[redacted]	[redacted]
[redacted]	[redacted]	[redacted]	[redacted]
[redacted]	[redacted]	[redacted]	[redacted]
[redacted]	[redacted]	[redacted]	[redacted]
[redacted]			

Table 13. [redacted]

[redacted]	[redacted]				[redacted]
	[redacted]		[redacted]		
	[redacted]	[redacted]	[redacted]	[redacted]	
[redacted]	[redacted]	[redacted]	[redacted]	[redacted]	[redacted]
[redacted]	[redacted]	[redacted]	[redacted]	[redacted]	[redacted]
[redacted]	[redacted]	[redacted]	[redacted]	[redacted]	[redacted]
[redacted]	[redacted]	[redacted]	[redacted]	[redacted]	[redacted]
[redacted]	[redacted]	[redacted]	[redacted]	[redacted]	[redacted]
[redacted]	[redacted]	[redacted]	[redacted]	[redacted]	[redacted]
[redacted]					

[redacted]

1.3.2.3. Efficacy and safety of the original adalimumab

Efficacy data for adalimumab in patients with psoriasis were obtained in three pivotal studies (M02-528 + M02-529, M03-656, M04-716), all of which had the similar design.

All patients included in these studies had moderate-to-severe plaque psoriasis. Most patients had PASI > 12, PGA > 3 (moderate-to-severe psoriasis), and BSA ≥ 20%. Most patients had previously used topical agents, phototherapy or photochemotherapy, or systemic non-biologic therapies.

The primary efficacy endpoint in all three pivotal studies was the proportion of patients who achieved PASI 75 (at least a 75% reduction in PASI from baseline). The secondary efficacy endpoints were as follows: the proportion of patients achieving a reduction in the sPGA score to “complete clearance” (score 0) or “minimal disease” (score 1); the proportion of PASI 50/90/100 responders; the time to response; relative PASI improvement (%); and relative sPGA improvement.

M02-528 study

This randomized, double-blind, multicenter study was conducted over 12 weeks. All patients who completed the study (148 subjects) were included in the extension study (M02-529). Patients in Arm 1 received adalimumab 80 mg at Week 0 as a starting dose followed by 40 mg every other week (starting from Week 1). Patients in Arm 2 received adalimumab 80 mg at Week 0 and adalimumab 80 mg at Week 1 followed by adalimumab 40 mg weekly. Patients in Arm 3 received placebo.

At Week 12, the proportion of patients with PASI 75 response was 53% in Arm 1 (40 mg maintenance dose given once every two weeks), 80% in Arm 2 (40 mg maintenance dose given weekly), and 4% in the placebo arm (P < 0.001) [1]. Although adalimumab 40 mg once a week showed higher efficacy, the proportion of PASI 75 responders at Week 24 (i.e. in the M02-529 extension) was 67% in Arm 1 and 77% in Arm 2. The differences were not statistically significant, and the dosing regimen used in Arm 1 (80 mg at Week 0 as a starting dose followed by 40 mg every other week starting from Week 1) was chosen for further research .

Most patients developed some adverse events (62.2% in Arm 1, 78.0% in Arm 2, and 67.3% in the placebo arm). The arms did not significantly differ in the frequencies of any adverse events. Serious adverse events were rare (1 episode in Arm 1 and 4 episodes in Arm 2). Cases of therapy discontinuation due to adverse events were also rare (2 subjects in Arm 1, 3 subjects in Arm 2, and 1 subject in the placebo arm).

M03-656 study

The study included three periods:

Period A (1200 patients included)

This period was double-blind, randomized, and placebo-controlled. Patients were randomly assigned at a 2:1 ratio to receive adalimumab (a starting dose of 80 mg at Week 0 followed by 40 mg once every two weeks from Week 1 to Week 16) or placebo. The PASI 75 response at Week 16 was set as the primary endpoint.

Period B (606 patients included)

Period B involved patients who achieved a PASI 75 response at Week 16 (during Period A). These patients received open-label adalimumab for 17 weeks (40 mg once every two week until Week 33).

Period C (490 patients included)

This period was double-blind, randomized, and placebo-controlled. The period involved patients in whom PASI 75 response sustained at Week 33 (at the end of Period B).

Patients who were randomized to receive adalimumab in Period A were re-randomized at a 1:1 frequency in Period C to one of two arms to receive either adalimumab 40 mg every other week or placebo. The subjects received adalimumab or placebo until Week 52 or until premature withdrawal, or until they have lost adequate response to therapy (whichever occurred first). Loss of response was defined as less than PASI 50 response after Week 33 (relative to baseline score) and/or at least a 6-point increase in PASI score from Week 33.

The primary endpoint in Period C was the proportion of patients losing an adequate response to therapy.

Figure 3 shows the flow chart of the M03-656 study.

Figure 3. M03-656: study flow chart

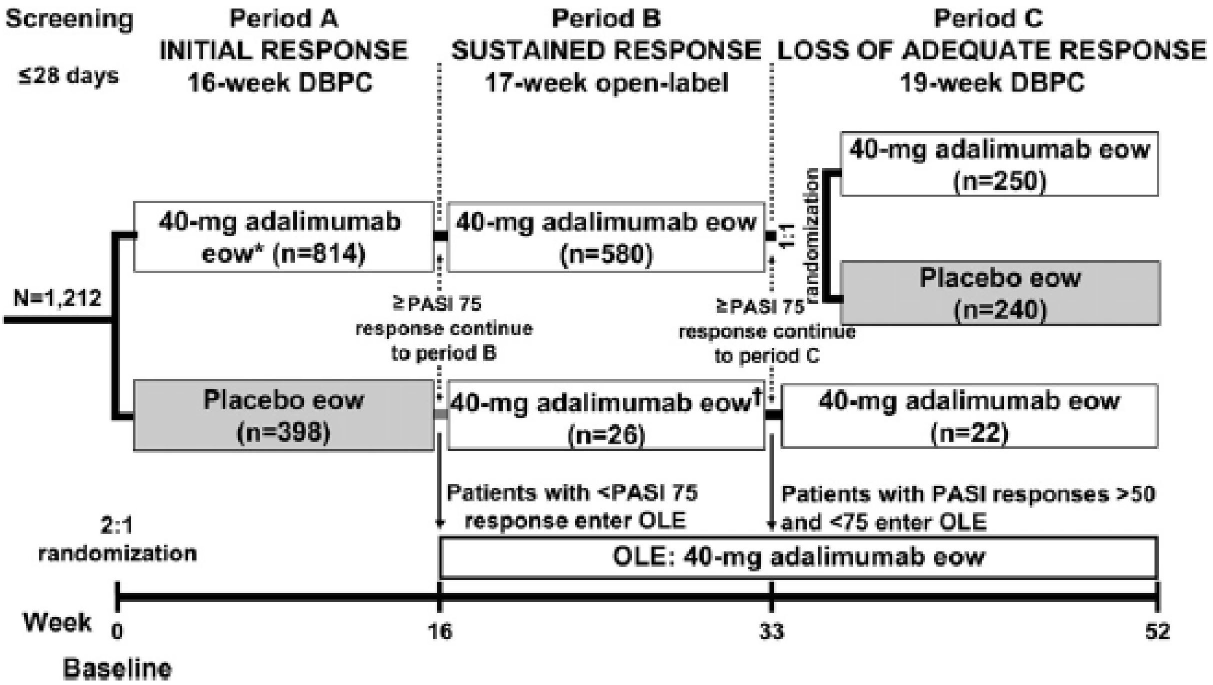

At Week 16 (at the end of Period A), 71.0% patients receiving adalimumab achieved a PASI 75 response versus 6.5% patients in the placebo arm ($P < 0.001$) [2].

At Week 31 (at the end of Period B), the majority of patients who were originally randomized to adalimumab in Period A maintained their response to treatment, while subjects who were originally randomized to placebo showed an improvement in their responses with adalimumab treatment.

At Week 52 (at the end of Period C), the frequency of losing adequate response to treatment was significantly lower in patients who were re-randomized to receive adalimumab compared to patients who were re-randomized to placebo (4.9% versus 28.4%, respectively).

During Period A, the majority of patients developed some adverse events (62.2% in the adalimumab arm and 55.5% in the placebo arm). Statistically significant differences between the arms were revealed in the incidence of all infections (28.9% with adalimumab and 22.4% with placebo), including upper respiratory tract infections (7.2% with adalimumab and 3.5% with placebo), headache, injection site reactions, joint pain, sinusitis, weakness, and itching. Serious adverse events were rare (1.8% patients in each arm). SAEs and AEs leading to discontinuation of study drug were also rare (1.7% in the adalimumab arm and 2.0% in the placebo arm).

During Period B, the incidence of adverse events was similar to that in period A. One patient who was initially randomized to receive adalimumab in period A got tuberculosis and received appropriate treatment. This patient had a positive tuberculin test at screening.

Safety data obtained by Week 52 (during Period C) have not been compared with those for placebo because these arms were not equivalent (the adalimumab arm involved only patients who responded to treatment). Injection site reactions were the most common type of AEs (1.7 patient-years). Infections were commonly reported (1.2 patient-years, including serious infections recorded in 0.02 patient-years). There were 1 case of tuberculosis (0.002 patient-years), 1 case of other opportunistic infections, 1 case of congestive cardiac failure, 1 case of an allergic reaction, 7 cases of non-melanoma skin cancer, and 2 cases of other malignancies. No cases of lymphoma were reported in adalimumab-treated patients.

M04-716 study

A 16-week randomized, double-blind, multicenter study evaluated the efficacy of adalimumab (starting dose of 80 mg at Week 0 followed by 40 mg every other week starting from Week 1) versus methotrexate capsules (7.5 mg to 25 mg every week) and versus placebo.

The study primary endpoint was significantly higher in the adalimumab treatment arm (79.6% of PASI 75 responders versus 35.5% with methotrexate and 18.9% with placebo) ($P < 0.001$) [3]. Adalimumab was shown to significantly improve the dermatology life quality index [4].

In this study, the majority of patients developed some adverse events (73.8% in the adalimumab arm, 80.9% in the methotrexate arm, and 79.2% in the placebo arm). The overall incidence of infections and the incidence of serious infections did not statistically significantly differ between the arms. Serious adverse events were rarely reported (2 cases [1.9%] in the adalimumab arm, 1 case [1.0%] in the methotrexate arm, and 1 case [1.9%] in the placebo arm). The incidence of adverse events resulting in treatment discontinuation was low (1 subject [1.0%] in the adalimumab arm, 6 subjects [5.5%] in the methotrexate arm, and 1 subject [1.9%] in the placebo arm).

Efficacy results from pivotal studies with adalimumab are summarized in Table 8.

Table 14. Efficacy results from adalimumab studies in patients with moderate-to-severe plaque psoriasis (n – number of patients who responded to treatment; N – total number of patients in the treatment arm)

Study, time of efficacy assessment	Placebo n/N (%)	Methotrexate n/N (%)	Adalimumab n/N (%)	p-value
		PASI 75		
M02−528, 12 weeks	2/52 (3.8)	—	24/45 (53.3)	< 0.001
M03-656, 16 weeks	26/398 (6.5)	—	578/814 (71.0)	< 0.001
M04-716, 16 weeks	10/53 (18.9)	38/110 (35.5)	86/108 (79.6)	< 0.001
		PGA score 0–1		
M03-656, 16 weeks	17/398 (4.3)	—	506/814 (62.2)	< 0.001

Study, time of efficacy assessment	Placebo n/N (%)	Methotrexate n/N (%)	Adalimumab n/N (%)	p-value
M04-716, 16 weeks	6/53 (11.3)	33/110 (30.0)	79/108 (73.1)	< 0.001

Results from this study demonstrated that the efficacy of adalimumab in moderate-to-severe plaque psoriasis is higher than the efficacy of methotrexate and placebo.

1.3.3. Conclusions and study rationale

Psoriasis is an multi-factor autoimmune disease significantly deteriorating the quality of patients' life, often resulting in social stigmatization and, in some cases, disability. It is estimated to affect 4% to 7% of the population in developed countries. Despite a significant progress that has been achieved with new treatment options, severe atypical treatment-resistant forms have become more common in the last decade. This determines the high social significance of psoriasis.

In the 1990s, pharmaceutical companies created several medicinal products that can significantly improve the clinical course of moderate-to-severe psoriasis. Adalimumab is one of the most commonly used medicines of this category. In clinical studies, adalimumab reduced PASI score by 75% or greater at Week 10 in 70.9% patients, while placebo showed only a 6.5% reduction [2]. Adalimumab was demonstrated to be superior to methotrexate: PASI 75 response at Week 16 was achieved in 79.6% patients treated with adalimumab versus 35.5% patients treated with methotrexate and 18.9% patients who received placebo [3].

Today, TNFα blockers are included in the international standards of care as a first-line biological therapy for moderate-to-severe plaque psoriasis. Some sources suggest that adalimumab, among all TNFα blockers, is the most efficient one in terms of the cost effectiveness [5]. However, adalimumab remains very expensive, and patients often cannot afford.

To make the therapy more available, BIOCAD has developed a biosimilar of adalimumab – BCD-057, which has the same pharmaceutical form and dosage as the originator. BCD-057 was investigated in a comprehensive non-clinical program, including animal studies, conducted in compliance with applicable international requirements. Non-clinical studies demonstrated that BCD-057 and Humira® have equivalent physicochemical, biological, PK, and toxicological properties. A Phase I study that involved 84 healthy volunteers showed that BCD-057 and Humira® are equivalent in their PK parameters and safety profiles.

Thus, BCD-057 can be recommended for further clinical investigation in the target patient population.

1.4. Brief description of known and potential risks and benefit for study subjects (benefit/risk balance)

BCD-057 is a biosimilar of the original Humira®.

The originator was studied in multiple clinical studies, which demonstrated its efficacy in patients with rheumatoid arthritis, ankylosing spondylitis, juvenile arthritis, ulcerative colitis, Crohn's disease, psoriatic arthritis, and plaque psoriasis. Today, adalimumab as the first-line biological therapy is a standard of care for certain autoimmune diseases. It significantly improves the patients' general health and quality of life, reduces the disease activity, and prevents early disability. BCD-057, the BIOCAD's adalimumab biosimilar, has the same active ingredient, dosage, and excipients as the marketed originator. The physicochemical and non-clinical studies *in vivo* and *in vitro* and the Phase I single-dose study of PK, tolerability and safety in healthy volunteers did not reveal any significant differences between BCD-057 and Humira®. Thus, it is expected that the efficacy and safety of BCD-057 in the planned clinical study will not significantly differ from those of Humira®.

1.4.1. Benefit assessment

The patients participating in the study will benefit from a detailed medical examination and biological therapy with adalimumab, which may improve the clinical course of the disease. Clinical improvement includes a PASI reduction (PASI 75 response may be achieved by up to 80% of patients, according to results from clinical studies of the originator), an sPGA reduction to score 0 (complete clearance) or score 1 (minimal disease) (may be achieved by 60% to 70% patients), and a reduction in the BSA. If the clinical course of psoriasis improves, patients can expect such beneficial effects as improved quality of life, decreased social stigmatization, and reduced risks of psoriasis-associated conditions (for example, psoriatic arthritis). Literature data suggest a potential for a long-term remission in patients with plaque psoriasis. Therefore, study subjects may achieve a steady clinical improvement with a decreased need for other treatments.

1.4.2. Risk assessment

Considering the wide clinical experience gained with the originator Humira® and results from previous clinical studies, it can be concluded that the safety of adalimumab has been well investigated.

The most dangerous adverse effects of adalimumab include serious infections and malignancies [6].

Serious infections that have been reported with adalimumab include tuberculosis, bacterial sepsis, invasive fungal diseases (such as histoplasmosis), and opportunistic infections caused by opportunistic bacteria, viruses, mycobacterium, fungi, parasites, or other microorganisms. In 34 controlled clinical studies, the risk of serious infections in patients receiving adalimumab was 4.6

cases per 100 patient-years, while that for placebo was 3.1 cases per 100 patient-years. In 47 controlled clinical studies, the incidence of tuberculosis was 0.22 cases per 100 patient-years and the risk of tuberculin test conversion was 0.08 per 100 patient-years. Most cases of tuberculosis were recorded during the first 8 months of therapy with adalimumab. This may suggest reactivation of the latent infection. Some cases of tuberculosis resulted in death. In three pivotal studies where patients received adalimumab for psoriasis, no cases of tuberculosis were reported. The M04-716 study involved 89 patients with psoriasis and latent tuberculosis. All patients received preventive treatment for 9 months, and only 2 patients (2.2%) developed the active disease during the treatment with adalimumab.

Malignancies have been reported in patients receiving adalimumab. In controlled clinical trials, the incidence of malignancies in psoriasis patients receiving adalimumab was 2.4 cases per 100 patient-years *vs*. 1.4 cases per 100 patient-years with placebo [7].

In controlled clinical studies, psoriasis patients receiving Humira® developed the following adverse events.

Infections (nasopharyngitis, upper respiratory tract infections, and sinusitis) were the most common AEs. In clinical studies in psoriasis, subjects receiving adalimumab developed infections more frequently than subjects receiving placebo (30.3% *vs*. 23.9%) [7].

Injection site reactions (erythema, itch, pain, and swelling in the injection site) were also more frequent in patients receiving adalimumab than in patients receiving placebo (7.0% *vs*. 5.0%) [7].

The incidence of other adverse events in patients receiving adalimumab was not higher than in subjects receiving placebo. In all the studies, AEs of special interest were demyelinating diseases of the CNS, lupus-like syndrome, congestive heart failure, and allergic reactions. Of these adverse events, allergic reactions were reported in the three controlled pivotal clinical studies of adalimumab in psoriasis. In all patients treated with adalimumab in clinical studies, there were 3 cases of allergic reactions (0.2/100 patient-years), 1 case (0.1/100 patient-years) of congestive cardiac failure, and 1 case (0.1/100 patient-years) of lupus-like syndrome. No cases of demyelinating diseases were reported. Three patients (0.2/100 patient-years) had CBC abnormalities (thrombocytopenia and leukopenia). Fifty-eight patients had hepatic reactions (most of them were elevations of AST or ALT). However, the controlled studies showed that the risk of such events in the adalimumab arm was lower than in the placebo arm (1.8% and 2.6%, respectively).

In clinical studies of adalimumab in psoriasis patients, 77 subjects (8.4%) developed antibodies to adalimumab. These cases were not associated with any symptoms or laboratory abnormalities. However, they decreased the efficacy of adalimumab, and patients with anti-adalimumab antibodies had significantly lower therapy results.

To ensure the safety of the study subjects, the Protocol specifies the inclusion/exclusion criteria, which are set to minimize risks of the adverse events described above. Due to known a known increase in the incidence of infections during the treatment with adalimumab, the study will not include patients with tuberculosis, any other active infections, or patients who have a high risk of getting an infection. The study subjects will undergo all necessary tests for tuberculosis, syphilis, hepatitis B and C, and HIV.

To reduce the frequency of allergic reactions to adalimumab, the study will not include patients with atopic bronchial asthma, hypersensitivity to any ingredients of BCD-057/Humira®, or a history of angioedema.

To reduce the risk of malignancies, the study will not include patients with malignancies in remission of less than 5 years.

To reduce the risk of congestive cardiac failure, the study will not include patients with congestive cardiac failure of NYHA classes III or IV, unstable angina or stable effort angina of functional classes III or IV, patients who had a myocardial infarction within 1 year before inclusion in the study, and patients with therapy-resistant hypertension.

To reduce the risk of demyelinating diseases of the CNS, the study will not include patients who already have any of these diseases at screening or ever had any individual symptoms suggestive of such a disease.

To prevent CBC or biochemistry abnormalities, the study will not include patients with those revealed at screening. Hematology and biochemistry parameters will be monitored during the study to detect these abnormalities if they appear.

Patients with moderate-to-severe plaque psoriasis may experience exacerbations of the disease. To prevent these situations and adequately assess the effects of adalimumab, the study will include patients with plaque psoriasis diagnosed at least 6 months before signing the informed consent.

1.4.3. Conclusions

Comparative physicochemical and non-clinical studies as well as the Phase I study of the PK, tolerability, and safety in healthy volunteers did not show any statistically significant differences between BCD-057 and Humira®. Thus, it is expected that, in this study in patients with moderate-to-severe plaque psoriasis, BCD-057 will demonstrate the efficacy, safety, immunogenicity, and PK similar to those of the originator.

The risks of adverse events have been thoroughly investigated. The treatment with adalimumab very rarely results in severe or life-threatening reactions and is expected to have low immunogenicity. At the same time, multiple international clinical studies of TNFα blockers and the wide clinical experience gained with Humira® suggest the benefit for the patients participating in the study. In

adalimumab clinical studies, a PASI 75 response was achieved by 80% patients, a reduction in sPGA to score 0 or 1 was achieved by up to 70%, the BSA decreased, and the patients' quality of life improved. Thus, the total risk/benefit ratio for subjects participating in this clinical study is considered favorable.

1.5. Description and justification of route of administration, doses, dosing regimen and treatment course

1.5.1 Design description and justification

This is a double-blind, randomized clinical study of the efficacy and safety conducted in two parallel arms.

The double-blind design is used to minimize the bias during the physician's and patient's assessment of treatment results, minimize systemic errors, and, therefore, to improve the validity of study results.

Stratification procedure ensures that any factors that can affect treatment results are uniformly distributed between the study arms, thus allowing investigators to attribute differences in responses directly to the investigational product. Some authors suggested a relationship between the patient's body weight (or BMI) and response to adalimumab [2, 10]. Similar trends were reported for other biologics used for the treatment of psoriasis [9, 10]. With the current data and the fact that the adalimumab dose is not titrated depending on the patient's body weight, all included patients will be stratified by body weight (< 80 kg / ≥ 81 kg). The M02-528 study also used stratification by body weight [1].

In addition, the patients will be stratified according to the prior exposure to antibodies for the treatment of psoriasis (experienced / naive), PASI score (< 20 / ≥ 20), and the presence of psoriatic arthritis (no / yes).

The active comparator allows making an objective comparison between the investigational products because treatment conditions were absolutely similar in both arms.

BCD-057 is a biosimilar of Humira®, which demonstrated its clinical efficacy and safety in large clinical trials and in a post-marketing setting. Humira® is used as a reference drug in this study. The choice of the reference drug completely meets the EMA's guideline on similar biological medicinal products containing monoclonal antibodies (EMA/CHMP/BMWP/403543/2010).

The main study hypothesis is that BCD-057 is equivalent to the original Humira®. According to the EMA's guidelines (EMA/CHMP/BMWP/403543/2010), this hypothesis is the most appropriate one to confirm the similarity between a biosimilar and the originator in a clinical study.

Each patient will be observed for 55 weeks (about one calendar year). This time will allow assessing not only the primary endpoint (the proportion of patients achieved PASI 75 at Week 16), but also analyzing long-term efficacy indicators and thoroughly investigating the immunogenicity and safety of BCD-057 *vs*. the originator in patients with moderate-to-severe plaque psoriasis. This study duration is recommended by the EMA's guideline on clinical investigation of medicinal products indicated for the treatment of psoriasis (EMEA/CHMP/EWP/2454/02).

The primary endpoint in this study is the proportion of patients with a PASI 75 response at Week 16. This is a sensitive indicator for patients with moderate-to-severe plaque psoriasis. This measure is widely used in clinical studies to assess the efficacy of medicinal products for the treatment of psoriasis and is recommended by the EMA's guideline as the primary endpoint to assess the efficacy of medicinal products indicated for the treatment of psoriasis (EMEA/CHMP/EWP/2454/02). The time of the primary endpoint evaluation was chosen based on the PK of adalimumab (time to steady state) and is appropriate to evaluate the drug effects according to the EMA's guideline. The use of PASI alone may be not enough for the comprehensive efficacy assessment, so the clinical course of psoriasis in the study will be assessed in detail by analyzing the changes in the BSA, changes in the sPGA score, changes in the absolute PASI scores, and other indicators. This agrees with the EMA's Guideline on *Clinical Investigation of Medicinal Products Indicated for the Treatment of Psoriasis* (EMEA/CHMP/EWP/2454/02).

This study is to compare the pharmacokinetics of BCD-057 and Humira® in a limited patient population (90 subjects from each study arm, 180 subjects total). This investigation is necessary to demonstrate that pharmacokinetics of these drugs is similar not only in healthy volunteers but in psoriasis patients as well (who may differ from healthy subjects in terms of adalimumab PK). In patients with plaque psoriasis, adalimumab is used for a long time, so it is important to study its PK parameters at steady state – when an equilibrium between the intake of the drug and its elimination has been achieved. Adalimumab fully exerts its clinical effects when reaches the steady state concentration. In addition, steady-state PK allows predicting how rapidly the serum levels of the active ingredient decrease is the patient misses a dose or discontinues the drug.

It is known that the time to steady state equals 3 to 5 half-life periods of adalimumab. For adalimumab, the average half-life is 2 weeks, varying from 10 to 20 days [8]. Thus, the maximum time to steady state will be 100 days or 14.3 weeks (20 × 5 = 100). According to literature data, adalimumab concentrations reach the steady state in 12 to 16 weeks.[11,12] Blood specimens will be

[11] Adalimumab M04-688 Pharmacokinetic Report
http://www.abbvie.com/assets/research/clinical-trials-data-and-information-sharing/synopses/adalimumab_M04-688.pdf
[12] Kobayashi S, Harigai M, Mozaffarian N, Pangan AL, Sharma S, Brown LS, Miyasaka N. A multicenter, open-label, efficacy, pharmacokinetic, and safety study of adalimumab in Japanese patients with ankylosing spondylitis. Mod Rheumatol. 2012 Aug;22(4):589-97.

taken at frequent intervals after the 9th injection of adalimumab (at weeks 15 and 16), and thus the PK of BCD-057/Humira® will be studied at steady state.

In the second period of the study, some patients will be crossed over from one drug product to another. After completing Week 24 and all appropriate assessments, one half of patients from the Humira® arm will be switched to BCD-057. The rest of patients who were originally randomized to receive Humira® will continue the treatment with Humira®.

The same study design was used in the study of the efficacy, safety, and immunogenicity of ABP501 adalimumab biosimilar in patients with psoriasis[13]. Originally, the patients were randomized at a 1:1 frequency to receive ABP501 or Humira®. After the first 16 weeks, patients from the Humira® arm were re-randomized 1:1 to two equal arms, one of which continued the treatment with Humira® and another one was switched to ABP501. Results from this study showed that switching from Humira® to the biosimilar did not have any negative effects on either efficacy or safety of the treatment.

Switching some patients to adalimumab biosimilar will not affect the validity of adalimumab equivalence assessment as the primary endpoint to evaluate the equivalence of BCD-057 and Humira® will be assessed at Week 16, which is before switching. Assessment of the secondary endpoints in the second study period will include not only the direct comparison between the study arms that have been receiving the same drug throughout the entire study but also the comparison of these study arms with the crossover arm. Thus, switching some patients from the original adalimumab to the biosimilar will provide additional information about the study drug, particularly will demonstrate the effect of drug switching on the efficacy and safety of adalimumab therapy.

Therefore, the proposed study design is justified from both scientific and practical points of view. The design meets the EMA's *Guideline on Similar Biological Medicinal Products Containing Monoclonal Antibodies* [EMA/CHMP/BMWP/403543/2010, 2012] and *Guideline on Clinical Investigation of Medicinal Products Indicated for the Treatment of Psoriasis* [EMEA/CHMP/EWP/2454/02 corr, 2004]. It is also consistent with available data on the mechanism of action, contraindications, and adverse effects of Humira®.

1.5.2. Description and justification of route of administration, doses, dosing regimen and treatment course

The patients will receive adalimumab (BCD-057 or Humira®) subcutaneously according to the following regimen: 80 mg on Day 1 of Week 0 (starting dose) and then 40 mg on Day 1 of Week 1,

[13] BLA 761024 ABP 501, a proposed biosimilar to Humira (adalimumab) FDA Briefing Document Arthritis Advisory Committee Meeting July 12, 2016

Week 3, Week 5, Week 7, Week 9, Week 11, Week 13, Week 15, Week 17, Week 19, Week 21, Week 23, Week 25, Week 27, Week 29, Week 31, Week 33, Week 35, Week 37, Week 39, Week 41, Week 43, Week 45, Week 47, Week 49, and Week 51. The patients will be followed up until Week 55.

The dose and method of administration (SC injection) of BCD-057 agrees with the recommendations presented in the Russian IMU for the marketed originator Humira® (MA ЛС-002422 of February 28, 2011) for the treatment of moderate-to-severe plaque psoriasis. These recommendations are based on the efficacy and safety data from the largest controlled clinical studies of various doses of Humira® in patients with moderate-to-severe plaque psoriasis. Two of three pivotal studies used the same dose and dosing regimen as those used in this study. Another Humira® clinical study in psoriasis used 2 regimens of adalimumab administration: Patients of Arm 1 received adalimumab 80 mg at Week 0 as a starting dose followed by 40 mg every other week (starting from Week 1). Patients in Arm 2 received adalimumab 80 mg at Week 0 and adalimumab 80 mg at Week 1 followed by adalimumab 40 mg weekly. The proportion of patients with PASI 75 response at Week 12 was 53% in Arm 1 and 80% in Arm 2. Thus, the treatment efficacy was higher in patients who received adalimumab according to the intensified regimen [1]. However, Arm 2 demonstrated the higher frequency of adverse events than Arm 1 (any AEs were reported in 78.0% *vs*. 62.2%, respectively). Thus, for better safety of adalimumab, it was decided to use the following regimen: 80 mg at Week 0 as a starting dose followed by 40 mg every other week (starting from Week 1).

Thus, the method of administration, dose, and dosing regimen are justified and agree with the study objectives. This dosing regimen for BCD-057 also meets the recommendations for the use of the original in patients with moderate-to-severe plaque psoriasis as described in the Russian Humira® Instruction for Medical Use (ЛС-002422 of February 28, 2011) and the official data presented on the FDA’s website [6].

1.6. Clinical study compliance with regulatory requirements

This clinical study will be conducted in accordance with the Protocol. The clinical study was developed in compliance with the GCP principles, current law and regulatory requirements of the participating countries.

1.7. Description of study population

Men and women of 18 to 75 years old with definite moderate-to-severe plaque psoriasis that was diagnosed at least 6 months before signing the informed consent. This study does not involve patients who have already received adalimumab or any other TNF alpha blockers and patients who have already received two or more biologics for the treatment of psoriasis.

1.8. References

1. Gordon KB, Langley RG, Leonardi C, Toth D, Menter MA, Kang S, Heffernan M, Miller B, Hamlin R, Lim L, Zhong J, Hoffman R, Okun MM. Clinical response to adalimumab treatment in patients with moderate to severe psoriasis: double-blind, randomized controlled trial and open-label extension study. J Am Acad Dermatol. 2006 Oct;55(4):598-606.
2. Menter A, Tyring SK, Gordon K, Kimball AB, Leonardi CL, Langley RG, Strober BE, Kaul M, Gu Y, Okun M, Papp K. Adalimumab therapy for moderate to severe psoriasis: A randomized, controlled phase III trial. J Am Acad Dermatol. 2008 Jan;58(1):106-15.
3. Saurat JH, Stingl G, Dubertret L, Papp K, Langley RG, Ortonne JP, Unnebrink K, Kaul M, Camez A; CHAMPION Study Investigators. Efficacy and safety results from the randomized controlled comparative study of adalimumab vs. methotrexate vs. placebo in patients with psoriasis (CHAMPION). Br J Dermatol. 2008 Mar;158(3):558-66.
4. Revicki D, Willian MK, Saurat JH, Papp KA, Ortonne JP, Sexton C, Camez A. Impact of adalimumab treatment on health-related quality of life and other patient-reported outcomes: results from a 16-week randomized controlled trial in patients with moderate to severe plaque psoriasis. Br J Dermatol. 2008 Mar;158(3):549-57.
5. Rudakova A.V., Kubanov A.A. Pharmacoeconomics aspects of treating psoriasis with biological medicinal products // Dermatology and Venerology Bulletin. - No 2. - P. 26-31.
6. Humira. Full prescribing information. Revised: 12/2014.
7. EMA. Scientific discussion. Humira (EMEA/H/C/481/II/38). London, 15 November 2007.
8. Humira, Summary of Product Characteristics for the Medicinal Product for Human Use, Russian Version (LS-002422).
9. Naldi L, Addis A, Chimenti S, Giannetti A, Picardo M, Tomino C, Maccarone M, Chatenoud L, Bertuccio P, Caggese E, Cuscito R. Impact of body mass index and obesity on clinical response to systemic treatment for psoriasis. Evidence from the Psocare project. Dermatology. 2008;217(4):365-73.
10. Edson-Heredia E, Sterling KL, Alatorre CI, Cuyun Carter G, Paczkowski R, Zarotsky V, Maeda-Chubachi T. Heterogeneity of response to biologic treatment: perspective for psoriasis. J Invest Dermatol. 2014 Jan;134(1):18-23.
11. Federal law 61-FZ dated 12.04.2010 “Concerning the circulation of medicinal products”.
12. National standard of the Russian Federation GOST R 52379-2005: Good Clinical Practice (approved by Decree No 232-st of September 27, 2005 of the Federal Agency for Technical Regulation and Metrology).

13. Decree No 266 "Concerning the approval of Good Clinical Practice in the Russian Federation", 19.06.2003.
14. World Medical Association Declaration of Helsinki (amended in 2013, 64th WMA General Assembly, Fortaleza, Brazil, October 2013).
15. Abbott Laboratories. Adalimumab M04-716 Clinical Study Report. Synopsis. R&D/05/057. http://www.abbvie.com/assets/research/clinical-trials-data-and-information-sharing/synopses/M04-716.pdf
16. Abbott Laboratories. Adalimumab M03-656 Clinical Study Report. R&D/05/712. Synopsis. http://www.abbvie.com/assets/research/clinical-trials-data-and-information-sharing/synopses/adalimumab_M03-656.pdf
17. Center for drug evaluation and research. Application Number sBLA 125057/110. Clinical pharmacology and biopharmaceutics review(s) http://www.accessdata.fda.gov/drugsatfda_docs/nda/2008/125057s110_ClinPharmR.pdf
18. Kubanova A.A., Kubanov A.A., Nikolas G.F., Puig L., Prince J., Katunina O.R., Znamenskaya L.F. Immune mechanisms of psoriasis. New strategies of biological therapy.// Vestn Dermatol Venerol 2010; 1: 35–47.
19. Lu Y., Chen H., Nikamo P., Low H.Q., Helms C., Seielstad M., Liu J., Bowcock A.M., Stahle M., Liao W. Association of Cardiovascular and Metabolic Disease Genes with Psoriasis // Journal of Investigative Dermatology (2013) 133,836–83.
20. Kubanova A.A. Dermatology in Russia. Reality and prospects / A.A. Kubanova, L. I. Tikhonova // Dermatology and Venerology Bulletin. 2004. - No.2. - P. 4-11.
21. Bhosle M.J., Kulkarni A., Feldman S.R., Balkrishnan R. Quality of life in patients with psoriasis. // Health and Quality of Life Outcomes 2006, 4:35.
22. In Touch with Psoriasis: Topical Treatments and Current Guidelines / G. Murphy, K. Reich. Attending Physician. 2013. No 5. P. 32
23. Olisova O.Yu. Current Approaches to Management of Psoriasis. Rus. Med. Journ. 2004;12(4): 182-5.
24. Vladimirov V. V., Menshikova L.V. Current Approaches to the Treatment of Psoriasis// Russian Medical Journal, 2001, V.6, No 20, p.1318–1323.
25. Kofoed K, Skov L, Zachari C New Drugs and Treatment Targets in Psoriasis Acta Derm Venereol. 2014 Aug 11.
26. Kurdina M.I. Anticytokine therapy: a new approach in treatment of psoriasis // Dermatology and Venerology Bulletin.

27. Rau R. Adalimumab (a fully human anti-tumour necrosis factor α monoclonal antibody) in the treatment of active rheumatoid arthritis: the initial results of five trials. Ann Rheum Dis 2002;61(Suppl II):ii70–ii73.
28. Goffe B. Etanercept (Enbrel) -- an update. Skin Therapy Lett. 2004 Dec-2005 Jan;9(10):1-4, 9.
29. Gottlieb A, Narang K. Ustekinumab in the treatment of psoriatic arthritis: latest findings and clinical potential. Therapeutic Advances in Musculoskeletal Disease. 2013;5(5):277-285.

2. Study objective and goals

2.1 Study objectives

Primary study objective:

- To investigate the efficacy and safety of BCD-057 versus Humira® in patients with moderate-to-severe plaque psoriasis diagnosed at least 6 months before signing the informed consent form.

Secondary objectives:

- To compare the efficacy of BCD-057 and Humira® by evaluating the proportion of patients who achieve a PASI 75 response, the changes in the BSA.
- To study the steady-state pharmacokinetics parameters BCD-057 and Humira® in a limited population.
- To assess the safety and immunogenicity of BCD-057 versus Humira®.

2.2 Study goals

1. To determine the proportion of patients achieving PASI 75 response at Week 16, by study arms.
2. To determine the proportion of patients achieving PASI 75 response at weeks 24, 33, and 55, by study arms.
3. To determine the proportion of patients achieving PASI 50/90 response at weeks 16, 24, 33, and 55, by study arms.
4. To assess changes from baseline in PASI score (%) at weeks 16, 24, 33, and 55, by study arms.
5. To determine the proportion of patients achieving a reduction in the sPGA score to "complete clearance" (score 0) or "minimal disease" (score 1) at weeks 16, 24, 33, and 55, by study arms.
6. To assess changes from baseline in the affected body surface area (BSA) at weeks 16, 24, 33, and 55, by study arms.
7. To evaluate the improvement from baseline in the nail psoriasis severity index (NAPSI) at weeks 16, 24, 33, and 55, by study arms.
8. To assess change from baseline in the itch severity (VAS score from 0 to 100 mm) at weeks 16, 24, 33, and 55, by study arms.
9. To determine the key PK parameters ($AUC_{tau,ss}$, $C_{av,ss}$, $C_{max,ss}$, $C_{min,ss}$, C_{trough}) of adalimumab at steady state. Limited population (180 patients total, which includes 90 patients from the BCD-057 arm and 90 patients from the Humira® arm).
10. To assess changes from baseline in the quality of life (DLQI and SF-36) at weeks 16, 24, 33, and 55, by study arms.

11. To determine the proportion of patients developing binding/neutralizing anti-adalimumab antibodies, by study arms. To determine whether there is a correlation between the presence of neutralizing antibodies and the therapeutic efficacy of BCD-057/Humira®.
12. To determine the proportion of patients with treatment-emerging AEs/SAEs, by study arms.

3. Study hypothesis

This clinical study is based on the hypothesis that

The clinical efficacy of BCD-057 defined as the proportion of patients with PASI 75 response at Week 16 is equivalent to the clinical efficacy of the original Humira®.

4. Study design

4.1. Primary and secondary outcome measures to be assessed in the study

4.1.1 Primary endpoint

- The proportion of patients with a 75% or greater PASI improvement (reduction) from baseline (PASI 75) at Week 16.

4.1.2 Secondary endpoints

Efficacy endpoints:

- Changes from baseline in PASI (%) at weeks 16, 24, 33, and 55, by study arms.
- The proportion of patients with a 75% reduction from baseline in psoriasis area and severity (PASI 75 response) at weeks 24, 33, and 55.
- The proportion of patients with a 50% and 90% PASI reduction from baseline at weeks 16. 24, 33, and 55.
- The proportion of patients with sPGA score reduced to 0 (complete clearance) or 1 (minimal disease) at weeks 16, 24, 33, and 55.
- Changes from baseline in affected BSA (%) at weeks 16, 24, 33, and 55.
- Changes from baseline in NAPSI (%) at weeks 16, 24, 33, and 55.
- Change from baseline in the itch severity (VAS score from 0 to 100) at weeks 16, 24, 33, and 55.
- Changes from baseline in health-related quality of life (DLQI and SF-36 scores) at weeks 16, 24, 33, and 55.

Safety endpoints:

- The proportion of patients who developed AEs/SAEs that, in the investigator's opinion, are related to Humira® or BCD-057, by study arms.
- The proportion of patients who experienced grade 3/4 AEs (CTCAE v. 4.03) that, in the investigator's opinion, are related to Humira® or BCD-057.
- The proportion of patients who developed AEs potentially due to TNFα inhibitors, by study arms. Such AEs include infections, serious infections, opportunistic infections (infections caused by opportunistic pathogenic viruses or cellular organisms), tuberculosis, malignancies, demyelinating diseases, lupus-like syndrome, congestive heart failure, allergic reactions, injection site reactions, and CBC or blood biochemistry abnormalities.

- The proportion of patients who discontinued the study due to AEs/SAEs, by study arms.

Immunogenicity endpoints

- The proportion of patients with binding or neutralizing anti-adalimumab antibodies.
- The proportion of patients with binding or neutralizing anti-adalimumab antibodies and inadequate therapeutic response (achieved PASI 50 or less).

PK endpoints

(PK population includes only 90 subjects from each arm, 180 subjects total)

- $AUC_{tau,ss}$ after multiple SC injections of Humira® / BCD-057.
- $C_{av,ss}$, $C_{max,ss}$, $C_{min,ss}$, C_{trough} after multiple SC injections of Humira® / BCD-057.

4.2. Description of the study type/design, study flow-chart, study procedures and periods

Study design

This clinical study of the efficacy and safety of BCD-057 and Humira® in patients with moderate-to-severe plaque psoriasis is a multicenter, double-blind, randomized, parallel-group study with an active comparator (Phase III).

The study will include 344 patients with definite moderate-to-severe plaque psoriasis diagnosed at least 6 months before signing the informed consent (the diagnosis should be confirmed by source documents). These patients should have the BSA of at least 10%, the PASI score of at least 12, and the sPGA score of at least 3.

Before enrollment, all patients will be given the full information about this clinical study, its purpose and the risks associated with study participation. After signing the informed consent form, the patient will undergo a screening examination (for not more than 4 weeks), which aims at confirming that the patient meets the study eligibility criteria. The Protocol does not provide any additional requirements regarding the diet or physical activity during the screening or study period.

According to the results of the screening exam, the investigator will make a decision whether to include the patient in the study or not. The patients who met the eligibility criteria were centrally randomized (with double blinding) into one of the study arms at a 1:1 ratio. At randomization, all enrolled patients were first stratified by body weight (< 80 kg / ≥ 81 kg), prior use of antibodies for the treatment of psoriasis[14] (experienced / naive), PASI score (< 20 / ≥ 20), and presence of psoriatic arthritis (no / yes). Thus, after stratification, the arms will be balanced by all specified characteristics.

[14] Except for therapeutic monoclonal antibodies or their fragments that are specific for tumor necrosis factor alpha.

- Patients in Arm 1 (n = 172) will receive BCD-057 as SC injections given according to the following regimen: 80 mg on Day 1 of Week 0 followed by 40 mg on Day 1 of weeks 1, 3, 5, 7, 9, 11, 13, 15, 17, 19, 21, and 23.
- Patients in Arm 2 (n = 172) will receive Humira® as SC injections given according to the following regimen: 80 mg on Day 1 of Week 0 followed by 40 mg on Day 1 of weeks 1, 3, 5, 7, 9, 11, 13, 15, 17, 19, 21, and 23.

To evaluate the effects of switching from the originator to the biosimilar, patients will be re-randomized at Week 24. As a result of this second randomization, patients from the Humira® arm will be assigned at a 1:1 frequency to one of the two sub-arms.

- Patients in sub-Arm 1 will stay on Humira® 40 mg and receive injections on Day 1 of weeks 25, 27, 29, 31, 33, 35, 37, 39, 41, 43, 45, 47, 49, and 51.
- Patients in sub-Arm 2 will receive injections of BCD-057 40 mg on Day 1 of weeks 25, 27, 29, 31, 33, 35, 37, 39, 41, 43, 45, 47, 49, and 51.

To maintain the double-blind study design, patients from the BCD-057 arm will also be invited for re-randomization. However, in this case, this will be just a nominal procedure where patients will be assigned new randomization IDs and lot numbers. They will continue BCD-057 according to the following regimen: 40 mg on Day 1 of weeks 25, 27, 29, 31, 33, 35, 37, 39, 41, 43, 45, 47, 49, and 51.

Regardless of the treatment arm to which they are assigned, patients will be followed up until Week 55 (if the patient has not been earlier removed from the study).

Injections of BCD-057/Humira® will be given at the study sites by a responsible qualified member of the study team (nurse, doctor, etc.).

Patients who dropped out due to reasons other than AEs/SAEs (not safety-related) can be replaced before Week 16 at the discretion of the Sponsor. The pharmacokinetics of adalimumab will be investigated in a limited patient population, which includes 90 patients from each study arm (180 subjects total). This study comprises the screening period and 30 visits (for all patients) or 39 visits (for patients involved in the PK study).

Study periods

The study will include the following periods:

1. ***Screening:***

   Up to 4 weeks (before randomization and inclusion in the study).

2. ***Main Treatment Period:***

24 weeks (weeks 0 to 24).

3. ***Switching Treatments (Crossover):***

Weeks 25 to 51.

4. ***Follow-Up Period:***

Until Week 55 after the first injection of BCD-057/Humira® (i.e. 28 days after the last injection of BCD-057/Humira®).

The study flow-chart is presented in Figure 4.

Figure 4. Study flow-chart

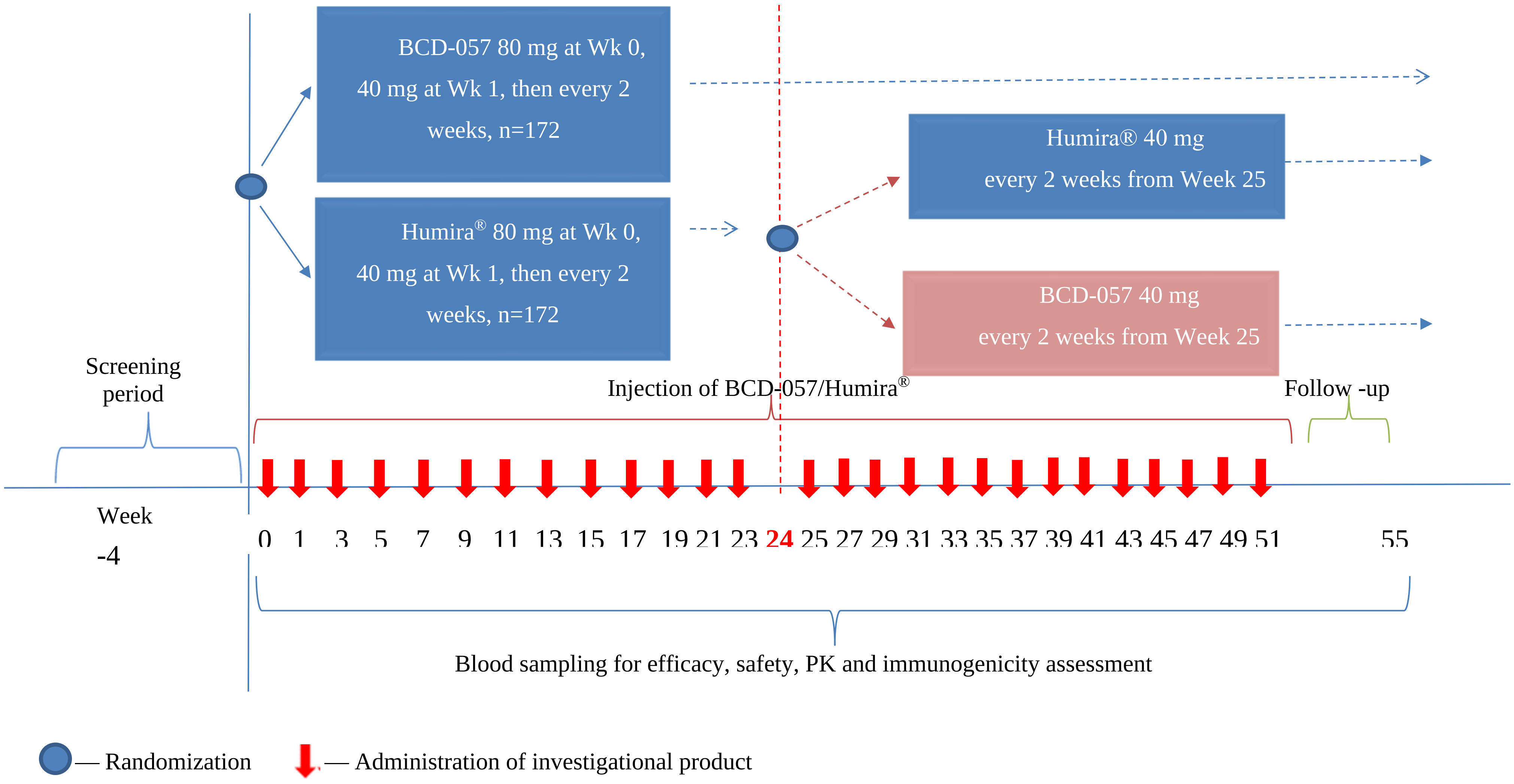

Study procedures

To establish whether patients meet the inclusion/exclusion criteria and to assess the treatment efficacy (for enrolled subjects), patients will undergo, within the timeframes specified by the Protocol, a comprehensive medical exam including:

- Baseline clinical characteristics and medical history
- Physical exam and measuring the body weight at screening
- BP and wrist pulse
- ECG
- Chest X-ray in posterior-anterior projection and/or fluorography
- CBC
- Blood biochemistry
- Urinalysis
- Infection status [Diaskintest® / QuantiFERON / T-Spot.TB test, HIV Ag/Ab Combo, p24 antigen assay, HBs-antigen, antibodies to HBcor (IgG + IgM/ IgG/ IgM) and antibodies to HCV, qualitative PCR for HCV RNA and/or HBV DNA (only in the case of positive results of the antibody assay), report provided by an infectious diseases specialist (only in the case of detected antibodies to HBcor or HCV), report from a TB specialist (in the case of indeterminant or positive TB test results), microprecipitation and TPHA (if results are positive, proceed to TPI test, ELISA, VDRL, or FTA-ABS at the discretion of the investigator, and obtain a consultation with a venereologist).
- Calculation of the BSA
- PASI assessment
- Static Physicians Global Assessment (sPGA)
- NAPSI assessment
- Itch intensity (VAS, 0 to 100 mm)
- Pregnancy test, for women only (the test is carried out using the urine HCG test strips) The pregnancy test is not required if the patient is at least 2 years post-menopausal or had a uterus or ovary surgery that makes pregnancy impossible.
- DLQI and SF-36 questionnaires

For safety evaluation, the following will be closely monitored: all general disorders (including fever and flu-like symptoms), abnormal vital signs (BP and wrist pulse), infectious complications, abnormal laboratory values (CBC results (hemoglobin, RBC, platelets, WBC including changes in

the WBC differential, and ESR); blood biochemistry (glucose, bilirubin total, ALT, AST, GGT, alkaline phosphatase, and creatinine), urinalysis, cardiac disorders (ECG), pulmonary conditions (chest X-ray and/or fluorography)).

Blood samples for immunogenicity study will be taken in patients of both arms. The specimens will be collected before the first dose and at weeks 16, 33, and 55. The specimens will be sent to the Central Laboratory for the assay of anti-adalimumab antibodies.

Additional blood samples for the PK assessment will be collected from a limited population (90 patients in each arm, 180 subjects total).

4.3. Measures to minimize/eliminate bias

4.3.1. Distribution of patients by study sites

Patients' recruitment at the study sites will be performed on a competitive basis.

4.3.2. Procedure of assigning study IDs

Patients will be randomized, stratified, and assigned study numbers according to the internal guidelines of JSC BIOCAD.

[redacted]

[redacted]

[redacted]

[redacted]

4.3.3. Stratification procedure

[redacted]

[redacted]

Table 15. [redacted]

[redacted]	[redacted]
[redacted]	[redacted]
[redacted]	[redacted]
[redacted]	[redacted]
[redacted]	[redacted]
[redacted]	[redacted]
[redacted]	[redacted]
[redacted]	[redacted]
[redacted]	[redacted]
[redacted]	[redacted]
[redacted]	[redacted]
[redacted]	[redacted]
[redacted]	[redacted]
[redacted]	[redacted]
[redacted]	[redacted]
[redacted]	[redacted]
[redacted]	[redacted]

[redacted]

4.3.4. Randomization procedure

Patients will be randomized centrally in both study periods. In the first study period, patients will be randomly distributed at a 1:1 ratio between two treatment arms.

[redacted]

[redacted]

Table 16. [redacted]

[redacted]	[redacted]	[redacted]	[redacted]	[redacted]	[redacted]
[redacted]	[redacted]	[redacted]	[redacted]	[redacted]	[redacted]
[redacted]	[redacted]	[redacted]	[redacted]	[redacted]	[redacted]
[redacted]	[redacted]	[redacted]	[redacted]	[redacted]	[redacted]
[redacted]	[redacted]	[redacted]	[redacted]	[redacted]	[redacted]

[redacted]

[redacted]

Table 17. [redacted]

[redacted]	[redacted]
[redacted]	[redacted]
	[redacted]
	[redacted]
	[redacted]
	[redacted]
[redacted]	[redacted]
	[redacted]
	[redacted]
	[redacted]
	[redacted]
[redacted]	[redacted]
	[redacted]
	[redacted]
	[redacted]
	[redacted]

[redacted]

[redacted]

A representative of JSC BIOCAD will monitor the total number of enrolled patients.

4.3.5. Blinding and subject-specific lots of investigational products

Neither investigators nor patients should know which investigational product is given to what patient. The investigator (the principal investigator, a co-investigator responsible for patient therapy or an authorized nurse) will receive BCD-057/Humira® supplied in identical packaging. The drugs will only differ by lot numbers and expiration dates. The lot number is subject-specific, and one drug lot covers the entire study period.

Each of the investigational products on its packaging will have a label with a 3-digit subject-specific lot number. Each syringe will have a label with the information about the lot of the investigational product:

Lot “001”,

Lot “002”,

Lot “003”,

…

Throughout the first study period, each patient will receive the investigational product from just one lot that is assigned to him/her at initial randomization. At re-randomization at Week 24, each patient will be assigned a new product lot that will be dispensed to him/her until the end of the study.

The lot number is recorded in the source documents, the CRF, and the study subject’s ID card.

The package of BCD-057/Humira® has a label containing a three-digit lot number (for the first period, before crossover) and a four-digit lot number (for the second period, post crossover). Each of these lot numbers are patient-specific.

During the study, CRAs from the BIOCAD’s Clinical Studies Division will perform audits of dispensed BCD-057 and Humira® accounting the number of syringes used.

Once the patient is included in the study (initial randomized), all necessary numbers are generated automatically after the investigator enters the data in the electronic IWRS system. The second randomization (re-randomization) will be a paper-based centralized randomization. In this case, the investigator will receive from the Sponsor the subject’s randomization number and investigational product lot number. The investigator will record the numbers in the source documents and the CRF.

4.4. Study therapy, doses, and dosage regimens of investigational products Pharmaceutical form, packaging, and labeling of investigational products

4.4.1. Study therapy, doses, and dosage regimens of investigational products

Injections of BCD-057/Humira® will be given at the study sites by a responsible qualified member of the study team (nurse, doctor, etc.).

1. Patients allocated to Arm 1 (n = 172) will receive BCD-057 (JSC BIOCAD) at a dose of 80 mg on Day 1 of Week 0 followed by a dose of 40 mg on Day 1 of weeks 1, 3, 5, 7, 9, 11, 13, 15, 17, 19, 21, and 23.
2. Patients allocated to Arm 2 (n = 172) will receive Humira® at a dose of 80 mg on Day 1 of Week 0 followed by a dose of 40 mg on Day 1 of weeks 1, 3, 5, 7, 9, 11, 13, 15, 17, 19, 21, and 23.

To evaluate the effects of switching from the originator to the biosimilar, patients will be re-randomized at Week 24. As a result of this second randomization, patients from the Humira® arm will be assigned at a 1:1 frequency to one of the two sub-arms.

- Patients in sub-Arm 1 will stay on Humira® 40 mg and receive injections on Day 1 of weeks 25, 27, 29, 31, 33, 35, 37, 39, 41, 43, 45, 47, 49, and 51.
- Patients in sub-Arm 2 will receive injections of BCD-057 40 mg on Day 1 of weeks 25, 27, 29, 31, 33, 35, 37, 39, 41, 43, 45, 47, 49, and 51.

To maintain the double-blind study design, patients from the BCD-057 arm will also be invited for re-randomization. However, in this case, this will be just a nominal procedure where patients will be assigned new randomization IDs and lot numbers. They will continue BCD-057 according to the following regimen: 40 mg on Day 1 of weeks 25, 27, 29, 31, 33, 35, 37, 39, 41, 43, 45, 47, 49, and 51.

Regardless of the treatment arm to which they are assigned, patients will be followed up until Week 55 (if the patient has not been earlier removed from the study).

4.4.2. Pharmaceutical form, packaging, and labeling of investigational products

The test drug and the comparator will have the similar packaging differing only by the lot number and expiration dates.

4.4.2.1. Test drug

[redacted]

[redacted]

4.4.2.2. Reference drug

[redacted]

4.4.2.3. Labeling of investigational products

This study is blind, so both investigational products will be labeled identically.

According to Article 46 of Federal Law 61-FZ “On the Circulation of Medicines” of April 12, 2010 and regulatory requirements of the participating countries, the primary packaging of the investigational product must contain the following information (clearly printed in the national language):

- Drug name and dosage: BCD-057 40 mg / Humira® 40 mg,
- Date of manufacture
- Shelf life
- Investigational product lot number.

The secondary packaging must contain the following information (clearly printed in the national language):

- Drug name and dosage: BCD-057 40 mg / Humira® 40 mg,
- Manufacturer: JSC BIOCAD, Russia / Vetter Pharma-Fertigung Gmbh & Co. KG, Germany
- Date of manufacture
- Shelf life
- Route of administration
- Dosage form
- Prescription status
- Storage conditions
- Warning phrases
- Investigational product lot number.

Both primary and secondary packages will also have the following labeling: “For Clinical Trials Only”.

The secondary package will also contain the following information: Protocol ID, study site number, and Subject ID code (entered by the investigator).

4.5. Expected duration of the study and subjects’ participation in the study

The expected duration of the study is 30 months. This includes patient recruitment (up to 12 months), treatment period, follow-up period, and collection and statistical processing of results.

Each subject will participate in the study for up to 59 weeks (about 14 months), including 4 weeks of screening, 51 weeks of treatment, and 4 weeks of follow-up.

4.6. Study periods

4.6.1 Study visits and procedures

The study includes the following periods:

1) **Screening:**

Up to 4 weeks (before randomization and inclusion in the study).

2) Main Treatment Period:

24 weeks (weeks 0 to 24).

3) **Switching Treatments (Crossover):**

Weeks 25 to 51.

4) Follow-Up Period:

Until Week 55 after the first injection of BCD-057/Humira® (i.e. 28 days after the last injection of BCD-057/Humira®).

Table 12 presents all assessments performed in the study. All data obtained during these assessments should be supported by appropriate records in the source documents.

The screening examination/baseline assessment should be performed within 28 days before the expected randomization date. Investigators are allowed to use previously obtained chest X-ray / fluorography results at screening (refer to section 4.7 for details). At the end of the screening period, the patient should be assessed for eligibility to see whether he/she meets the inclusion/exclusion criteria and then be randomized to one of the treatment arms.

Table 18. Study visits and procedures

Study period	Screening		Treatment period																																			Final visit
**Visit**	**Screening**	**1**	**2**	**3**	**4**	**5**	**6**	**7**	**8**	**9**	**9-1[1]**	**9-2[1]**	**9-3[1]**	**9-4[1]**	**9-5[1]**	**9-6[1]**	**10**	**10-1[1]**	**10-2[1]**	**10-3[1]**	**11**	**12**	**13**	**14**	**15**	**16**	**17**	**18**	**19**	**20**	**21**	**22**	**23**	**24**	**25**	**26**	**27**	**28**	**29**	**30**
**Study Week**	**-4-0**	**0**	**1**	**3**	**5**	**7**	**9**	**11**	**13**	**15**	**15**	**15**	**15**	**15**	**15**	**15**	**16**	**16**	**16**	**16**	**17**	**19**	**21**	**23**	**24**	**25**	**27**	**29**	**31**	**33**	**35**	**37**	**39**	**41**	**43**	**45**	**47**	**49**	**51**	**55**
**Day**		**1**	**1**	**1**	**1**	**1**	**1**	**1**	**1**	**1**	**2**	**3**	**4**	**5**	**6**	**7**	**1**	**2**	**4**	**6**	**1**	**1**	**1**	**1**	**1**	**1**	**1**	**1**	**1**	**1**	**1**	**1**	**1**	**1**	**1**	**1**	**1**	**1**	**1**	**1**
**Patient signs the Informed Consent Form**	X																																							
**Demographic data, life and disease history**	X																																							
**Eligibility (inclusion/exclusion criteria)**	X																																							
**Randomization**	X																								X															
**Chest x-ray/fluorography**	X[2]																									X														X
**Diaskintest®/ QuantiFERON test[3]**	X																									X														X
**Body weight**	X																																							
**Physical examination**	X	X	X	X	X		X		X								X						X		X			X		X		X		X		X		X		X
**BP, pulse, and body temperature**	X	X	X	X	X		X		X								X						X		X			X		X		X		X		X		X		X
**ECG**	X																								X															X
**CBC**	X				X		X		X								X						X		X							X						X		X
**Blood biochemistry**	X				X		X		X								X						X		X							X						X		X
**Take blood for PK assessment[4, 5]**		X	X	X	X	X	X	X	X	X[6]	X	X	X	X	X	X		X	X	X	X	X	X	X																
**Blood sampling for immunogenicity**		X															X													X										X
**Urinalysis**	X																								X															X
**Tests for HIV, HCV, HBV, and syphilis**	X																																							
**Pregnancy test[7]**	X																																							

Study period	Screening		Treatment period																																			Final visit
Visit	Screening	1	2	3	4	5	6	7	8	9	9-1[1]	9-2[1]	9-3[1]	9-4[1]	9-5[1]	9-6[1]	10	10-1[1]	10-2[1]	10-3[1]	11	12	13	14	15	16	17	18	19	20	21	22	23	24	25	26	27	28	29	30
Study Week	-4-0	0	1	3	5	7	9	11	13	15	15	15	15	15	15	15	16	16	16	16	17	19	21	23	24	25	27	29	31	33	35	37	39	41	43	45	47	49	51	55
Day		1	1	1	1	1	1	1	1	1	2	3	4	5	6	7	1	2	4	6	1	1	1	1	1	1	1	1	1	1	1	1	1	1	1	1	1	1	1	1
Psoriasis Area and Severity Index (PASI)	X	X			X		X		X								X						X		X			X		X		X				X				X
Static Physician's Global Assessment (sPGA)	X	X			X		X		X								X						X		X			X		X		X				X				X
BSA	X	X			X		X		X								X						X		X			X		X		X				X				X
Nail Psoriasis Severity Index (NAPSI)	X																X								X					X										X
Itch intensity assessment (VAS, 0 to 100 mm)	X																X								X					X										X
DLQI questionnaire[8]		X															X								X					X										X
SF-36 questionnaire8		X															X								X					X										X
Information about concomitant therapies	X	X	X	X	X	X	X	X	X	X	X	X	X	X	X	X	X	X	X	X	X	X	X	X	X	X	X	X	X	X	X	X	X	X	X	X	X	X	X	X
Injection of BCD-057/Humira®		X	X	X	X	X	X	X	X	X											X	X	X	X		X	X	X	X	X	X	X	X	X	X	X	X	X	X	
Documenting, recording, and reporting AE/SAE, including injection site reactions	X[9]	X	X	X	X	X	X	X	X	X	X	X	X	X	X	X	X	X	X	X	X	X	X	X	X	X	X	X	X	X	X	X	X	X	X	X	X	X	X	X

[1]This visit is performed only for the patients involved in the study of adalimumab pharmacokinetics.

[2] At screening, fluorography may be done instead of the chest X-ray. Screening chest X-ray is not required if the patient has results of the chest X-ray/fluorography/CT/MRI performed within 1 month before signing the informed consent form.

Patients with indeterminant and/or positive results of the QuantiFERON/T-Spot.TB test/Diaskintest® are allowed in the study only if all of the following three criteria are met: an additional QuantiFERON or T-Spot.TB test gives a negative result; no signs of active tuberculosis are seen on the chest X-ray/fluorography/CT/MRI) performed within 1 month before signing the informed consent form; and the TB specialist provides a written report during screening, confirming that the patient has no tuberculosis.

[4] The PK study will involve a limited population (90 patients from each study arm, 180 subjects total).

[5] Blood samples are taken in 15 min ± 5 min before the injection of adalimumab.

[6] PK on Day 1 of Week 15 is assessed 3 h, 12 h, 24 h, 48 h, 72 h, 96 h, 120 h, 144 h, 192 h, 240 h, and 288 h after the injection of adalimumab. These time points fall on days 1, 2, 3, 4, 5, 6, and 7 of Week 15 and Days 2, 4, and 6 of Week 16 (see Table 16).

Study period	Screening		Treatment period																																				Final visit
**Visit**	**Screening**	**1**	**2**	**3**	**4**	**5**	**6**	**7**	**8**	**9**	**9-1[1]**	**9-2[1]**	**9-3[1]**	**9-4[1]**	**9-5[1]**	**9-6[1]**	**10**	**10-1[1]**	**10-2[1]**	**10-3[1]**	**11**	**12**	**13**	**14**	**15**	**16**	**17**	**18**	**19**	**20**	**21**	**22**	**23**	**24**	**25**	**26**	**27**	**28**	**29**	**30**
**Study Week**	**-4-0**	**0**	**1**	**3**	**5**	**7**	**9**	**11**	**13**	**15**	**15**	**15**	**15**	**15**	**15**	**15**	**16**	**16**	**16**	**16**	**17**	**19**	**21**	**23**	**24**	**25**	**27**	**29**	**31**	**33**	**35**	**37**	**39**	**41**	**43**	**45**	**47**	**49**	**51**	**55**
**Day**		**1**	**1**	**1**	**1**	**1**	**1**	**1**	**1**	**1**	**2**	**3**	**4**	**5**	**6**	**7**	**1**	**2**	**4**	**6**	**1**	**1**	**1**	**1**	**1**	**1**	**1**	**1**	**1**	**1**	**1**	**1**	**1**	**1**	**1**	**1**	**1**	**1**	**1**	**1**

[7] Pregnancy test is carried out with the urine HCG test strips. The pregnancy test is not required if the patient is at least 2 years post-menopausal or had a uterus or ovary surgery that makes pregnancy impossible.
[8] The patient fills out the questionnaires on the visit day before receiving an injection of BCD-057/Humira® (if applicable).
[9] Only SAEs are documented.

4.6.2. Procedures by visits

Procedures of the screening period

The Screening Period starts from the moment the patient signs the informed consent form and continues for not more than 4 weeks (Days -28 to 0) until the day when the patient is included in the study. The Screening Period includes the following procedures:

- Signing the informed consent form.
- Demographic data, life and disease history.
- Data about concomitant therapy.
- Physical examination.
- Body weight.
- BP, wrist pulse, and body temperature.
- ECG.
- Chest x-ray / fluorography.
- Diaskintest® / QuantiFERON test[15] / T-Spot.TB[16].
- CBC[17] (at least 2 mL of blood).
- Blood biochemistry[20] (at least 4 mL of blood).
- Urinalysis.
- Tests for HIV, HCV, HBV, syphilis (at least 12 mL).
- BSA.
- PASI score.
- Static Physicians Global Assessment (sPGA).
- NAPSI.
- Itch intensity VAS score (0 to100 mm).
- Pregnancy test[18] (HCG in urine, test strip).
- Checking for eligibility (inclusion/exclusion criteria).
- Inclusion in the study (randomization for Period I).
- Documenting, recording, and reporting SAEs.

Total blood volume taken at the visit: at least 18 mL.

---

[15] If the QuantiFERON assay is done during this visit, the volume of blood taken will be at least 4 mL.
[16] If the T-Spot.TB assay is done during this visit, the volume of blood taken will be at least 6 mL.
[17]At screening, the test can be repeated once if the first result did not meet the eligibility criteria.
[18] Pregnancy test is carried out only in women. The urine HCG is measured with a test strip. The pregnancy test is not required if the patient is at least 2 years post-menopausal or had a uterus or ovary surgery that makes pregnancy impossible.

Procedures of Visit 1 (Week 0)

- Concomitant therapy.
- Physical examination.
- BP, wrist pulse, and body temperature.
- Blood sampling for PK study (before the injection of BCD-057/Humira®) (6 mL).
- Blood sampling for immunogenicity study (before the injection of BCD-057/Humira®) (8 mL).
- BSA.
- PASI score
- sPGA, DLQI and SF-36 (questionnaires filled in by the patient before the injection of BCD-057/Humira®).
- Injection of BCD-057/Humira®.
- Documenting, recording, and reporting AE/SAE, including injection site reactions.

Total blood volume taken at the visit: 8 mL for all patients; 14 mL for patients involved in the PK study.

Procedures of Visit 2 (Week 1)

- Concomitant therapy.
- Physical examination.
- Blood sampling for PK study (before the injection of BCD-057/Humira®) (6 mL).
- BP, wrist pulse, and body temperature.
- Injection of BCD-057/Humira®.
- Documenting, recording, and reporting AE/SAE, including injection site reactions.

Total blood volume taken at the visit: 6 mL for patients involved in the PK study.

Procedures of Visit 3 (Week 3)

- Concomitant therapy.
- Physical examination.
- BP, wrist pulse, and body temperature.
- Blood sampling for PK study (before the injection of BCD-057/Humira®) (6 mL).
- Injection of BCD-057/Humira®.
- Documenting, recording, and reporting AE/SAE, including injection site reactions.

Total blood volume taken at the visit: 6 mL for patients involved in the PK study.

Procedures of Visit 4 (Week 5)

- Concomitant therapy.

- Physical examination.
- BP, wrist pulse, and body temperature.
- CBC (2 mL of blood).
- Blood biochemistry (4 mL of blood).
- Blood sampling for PK study (before the injection of BCD-057/Humira®) (6 mL).
- BSA.
- PASI score.
- Static Physicians Global Assessment (sPGA).
- Injection of BCD-057/Humira®.
- Documenting, recording, and reporting AE/SAE, including injection site reactions.

Total blood volume taken at the visit: 6 mL for all patients; 12 mL for patients involved in the PK study.

Procedures of Visit 5 (Week 7)

- Concomitant therapy.
- Blood sampling for PK study (before the injection of BCD-057/Humira®) (6 mL).
- Injection of BCD-057/Humira®.
- Documenting, recording, and reporting AE/SAE, including injection site reactions.

Total blood volume taken at the visit: 6 mL for patients involved in the PK study.

Procedures of Visit 6 (Week 9)

- Concomitant therapy.
- Physical examination.
- BP, wrist pulse, and body temperature.
- CBC (2 mL of blood).
- Blood biochemistry (4 mL of blood).
- Blood sampling for PK study (before the injection of BCD-057/Humira®) (6 mL).
- BSA.
- PASI score.
- Static Physicians Global Assessment (sPGA).
- Injection of BCD-057/Humira®.
- Documenting, recording, and reporting AE/SAE, including injection site reactions.

Total blood volume taken at the visit: 6 mL for all patients; 12 mL for patients involved in the PK study.

Procedures of Visit 7 (Week 11)

- Concomitant therapy.
- Blood sampling for PK study (before the injection of BCD-057/Humira®) (6 mL).
- Injection of BCD-057/Humira®.
- Documenting, recording, and reporting AE/SAE, including injection site reactions.

Total blood volume taken at the visit: 6 mL for patients involved in the PK study.

Procedures of Visit 8 (Week 13)

- Concomitant therapy.
- Physical examination.
- BP, wrist pulse, and body temperature.
- CBC (2 mL of blood).
- Blood biochemistry (4 mL of blood).
- Blood sampling for PK study (before the injection of BCD-057/Humira®) (6 mL).
- BSA.
- PASI score.
- Static Physicians Global Assessment (sPGA).
- Injection of BCD-057/Humira®.
- Documenting, recording, and reporting AE/SAE, including injection site reactions.

Total blood volume taken at the visit: 6 mL for all patients; 12 mL for patients involved in the PK study.

Procedures of Visit 9 (Week 15)

- Concomitant therapy.
- Take blood for PK study (before the injection and 3 h and 12 h after the injection of BCD-057/Humira®) (3 blood samples of 6 mL each).
- Injection of BCD-057/Humira®.
- Documenting, recording, and reporting AE/SAE, including injection site reactions.
- Total blood volume taken at the visit: 18 mL for patients involved in the PK study.

Procedures of Visits 9-1, 9-2, 9-3, 9-4, 9-5, and 9-6 (Week 15). Visits performed only in patients involved in the PK study

- Concomitant therapy.
- Blood sampling for PK study (6 mL).
- Documenting, recording, and reporting AE/SAE, including injection site reactions.

The total volume of blood taken during each of the visits: 6 mL (limited population of patients involved in the PK study.

Procedures of Visit 10 (Week 16)

- Concomitant therapy.
- Physical examination.
- BP, wrist pulse, and body temperature.
- CBC (2 mL of blood).
- Blood biochemistry (4 mL of blood).
- Blood sampling for immunogenicity study (8 mL).
- BSA.
- PASI score.
- Static Physicians Global Assessment (sPGA).
- NAPSI.
- Itch intensity VAS score (0 to100 mm).
- Quality of life (DLQI and SF-36).
- Documenting, recording, and reporting AE/SAE, including injection site reactions.

Total blood volume taken at the visit: 14 mL (each patient).

Procedures of Visits 10-1, 10-2, and 10-3 (Week 16). Visits performed only in patients involved in the PK study

- Concomitant therapy.
- Blood sampling for PK study (6 mL).
- Documenting, recording, and reporting AE/SAE, including injection site reactions.

The total volume of blood taken during each of the visits: 6 mL (limited population of patients involved in the PK study.

Procedures of Visit 11 (Week 17)

- Concomitant therapy.
- Blood sampling for PK study (before the injection of BCD-057/Humira®) (6 mL).
- Injection of BCD-057/Humira®.
- Documenting, recording, and reporting AE/SAE, including injection site reactions.

Total blood volume taken at the visit: 6 mL (limited population of patients involved in the PK study.

Procedures of Visit 12 (Week 19)

- Concomitant therapy.
- Blood sampling for PK study (before the injection of BCD-057/Humira®) (6 mL).
- Injection of BCD-057/Humira®.
- Documenting, recording, and reporting AE/SAE, including injection site reactions.

Total blood volume taken at the visit: 6 mL (limited population of patients involved in the PK study.

Procedures of Visit 13 (Week 21)

- Concomitant therapy.
- Physical examination.
- BP, wrist pulse, and body temperature.
- CBC (2 mL of blood).
- Blood biochemistry (4 mL of blood).
- Blood sampling for PK study (before the injection of BCD-057/Humira®) (6 mL).
- BSA.
- PASI score
- Static Physicians Global Assessment (sPGA).
- Injection of BCD-057/Humira®.
- Documenting, recording, and reporting AE/SAE, including injection site reactions.

Total blood volume taken at the visit: 6 mL for all patients; 12 mL for patients involved in the PK study.

Procedures of Visit 14 (Week 23)

- Concomitant therapy.
- Blood sampling for PK study (before the injection of BCD-057/Humira®) (6 mL).
- Injection of BCD-057/Humira®.
- Documenting, recording, and reporting AE/SAE, including injection site reactions.

Total blood volume taken at the visit: 6 mL (limited population of patients involved in the PK study.

Procedures of Visit 15 (Week 24)

- Concomitant therapy.
- Physical examination.
- BP, wrist pulse, and body temperature.

- CBC (2 mL of blood).
- Blood biochemistry (4 mL of blood).
- Urinalysis.
- ECG.
- BSA.
- PASI score.
- Static Physicians Global Assessment (sPGA).
- NAPSI.
- Itch intensity VAS score (0 to100 mm).
- Quality of life (DLQI and SF-36).
- Documenting, recording, and reporting AE/SAE, including injection site reactions.
- Re-randomization for Period II.

Total blood volume taken at the visit: 6 mL (each patient)

Procedures of Visit 16 (Week 25)

- Concomitant therapy.
- Chest x-ray / fluorography.
- Diaskintest® / QuantiFERON test / T-Spot.TB.
- Injection of BCD-057/Humira®.
- Documenting, recording, and reporting AE/SAE, including injection site reactions.

Total blood volume taken at the visit: If the QuantiFERON or T-Spot.TB assay is done during this visit, the volume of blood taken will be at least 4 or 6 mL, respectively.

Procedures of Visit 17 (Week 27)

- Concomitant therapy.
- Injection of BCD-057/Humira®.
- Documenting, recording, and reporting AE/SAE, including injection site reactions.

Procedures of Visit 18 (Week 29)

- Concomitant therapy.
- Physical examination.
- BP, wrist pulse, and body temperature.
- BSA.
- PASI score.
- Static Physicians Global Assessment (sPGA).

- Injection of BCD-057/Humira®.
- Documenting, recording, and reporting AE/SAE, including injection site reactions.

Procedures of Visit 19 (Week 31)

- Concomitant therapy.
- Injection of BCD-057/Humira®.
- Documenting, recording, and reporting AE/SAE, including injection site reactions.

Procedures of Visit 20 (Week 33)

- Concomitant therapy.
- Physical examination.
- BP, wrist pulse, and body temperature.
- Blood sampling for immunogenicity study (8 mL).
- BSA.
- PASI score.
- Static Physicians Global Assessment (sPGA).
- NAPSI.
- Itch intensity VAS score (0 to100 mm).
- Quality of life (DLQI, SF-36). The patient fills out the questionnaires before the injection of BCD-057/Humira®).
- Injection of BCD-057/Humira®.
- Documenting, recording, and reporting AE/SAE, including injection site reactions.

  Total blood volume taken at the visit: 8 mL (each patient).

Procedures of Visit 21 (Week 35)

- Concomitant therapy.
- Injection of BCD-057/Humira®.
- Documenting, recording, and reporting AE/SAE, including injection site reactions.

Procedures of Visit 22 (Week 37)

- Concomitant therapy.
- Physical examination.
- BP, wrist pulse, and body temperature.
- CBC (2 mL of blood).
- Blood biochemistry (4 mL of blood).

- BSA.
- PASI score.
- Static Physicians Global Assessment (sPGA).
- Injection of BCD-057/Humira®.
- Documenting, recording, and reporting AE/SAE, including injection site reactions.

Total blood volume taken at the visit: 6 mL (each patient).

Procedures of Visit 23 (Week 39)

- Concomitant therapy.
- Injection of BCD-057/Humira®.
- Documenting, recording, and reporting AE/SAE, including injection site reactions.

Procedures of Visit 24 (Week 41)

- Concomitant therapy.
- Physical examination.
- BP, wrist pulse, and body temperature.
- Injection of BCD-057/Humira®.
- Documenting, recording, and reporting AE/SAE, including injection site reactions.

Procedures of Visit 25 (Week 43)

- Concomitant therapy.
- Injection of BCD-057/Humira®.
- Documenting, recording, and reporting AE/SAE, including injection site reactions.

Procedures of Visit 26 (Week 45)

- Concomitant therapy.
- Physical examination.
- BP, wrist pulse, and body temperature.
- BSA.
- PASI score.
- Static Physicians Global Assessment (sPGA).
- Injection of BCD-057/Humira®.
- Documenting, recording, and reporting AE/SAE, including injection site reactions.

Procedures of Visit 27 (Week 47)

- Concomitant therapy.
- Injection of BCD-057/Humira®.
- Documenting, recording, and reporting AE/SAE, including injection site reactions.

Procedures of Visit 28 (Week 49)

- Concomitant therapy.
- Physical examination.
- BP, wrist pulse, and body temperature.
- CBC (2 mL of blood).
- Blood biochemistry (4 mL of blood).
- Injection of BCD-057/Humira®.
- Documenting, recording, and reporting AE/SAE, including injection site reactions.

Total blood volume taken at the visit: 6 mL (each patient).

Procedures of Visit 29 (Week 51)

- Concomitant therapy.
- Injection of BCD-057/Humira®.
- Documenting, recording, and reporting AE/SAE, including injection site reactions.

Procedures of Visit 30 (Week 55)

- Concomitant therapy.
- Physical examination.
- BP, wrist pulse, and body temperature.
- CBC (2 mL of blood).
- Blood biochemistry (4 mL of blood).
- Blood sampling for immunogenicity study (8 mL).
- Urinalysis.
- Chest x-ray / fluorography.
- ECG.
- Diaskintest® / QuantiFERON test / T-Spot.TB
- BSA.
- PASI score.
- Static Physicians Global Assessment (sPGA).
- NAPSI.

- Itch intensity VAS score (0 to100 mm).
- Quality of life (DLQI and SF-36).
- Documenting, recording, and reporting AE/SAE, including injection site reactions.

Total blood volume taken at the visit: 14 ml (each patient). If the QuantiFERON or T-Spot.TB assay is done during this visit, the volume of blood taken will be at least 18 or 20 mL, respectively.

4.7. Description of individual study procedures

All scheduled clinical and laboratory procedures and timing are presented in Table 13.

Table 19. Clinical and laboratory examinations in the study

Test	Tested variable	Frequency	Where the test is performed
Physical examination	Height (at screening only)<br>Body weight (at screening only)<br>Nervous system<br>Skin, mucosal membranes (visual examination)<br>Respiratory system (auscultation of lungs)<br>Cardiovascular system (auscultation of heart)<br>GI tract (abdominal palpation)<br>Liver and spleen size (palpation)<br>Bowel and bladder habits (normal / abnormal)	× 17:<br>at screening,<br>Day 1 of Week 0<br>Day 1 of Week 1<br>Day 1 of Week 3<br>Day 1 of Week 5<br>Day 1 of Week 9<br>Day 1 of Week 13<br>Day 1 of Week 16<br>Day 1 of Week 21<br>Day 1 of Week 24<br>Day 1 of Week 29<br>Day 1 of Week 33<br>Day 1 of Week 37<br>Day 1 of Week 41<br>Day 1 of Week 45<br>Day 1 of Week 49<br>Day 1 of Week 55	Study site
Vital signs	BP<br>Pulse<br>Body temperature	× 17:<br>at screening,<br>Day 1 of Week 0<br>Day 1 of Week 1<br>Day 1 of Week 3<br>Day 1 of Week 5<br>Day 1 of Week 9<br>Day 1 of Week 13<br>Day 1 of Week 16<br>Day 1 of Week 21<br>Day 1 of Week 24<br>Day 1 of Week 29<br>Day 1 of Week 33<br>Day 1 of Week 37<br>Day 1 of Week 41<br>Day 1 of Week 45<br>Day 1 of Week 49<br>Day 1 of Week 55	Study site
CBC	Hemoglobin (g/L)<br>Hematocrit (%)<br>RBC ($\times 10^{12}$/L)<br>Platelets ($\times 10^{9}$/L)<br>WBC ($\times 10^{9}$/L)	× 10:<br>at screening,<br>Day 1 of Week 5<br>Day 1 of Week 9<br>Day 1 of Week 13	Study site or Central Laboratory

Test	Tested variable	Frequency	Where the test is performed
	Neutrophils (×10^9/L)<br>Lymphocytes (×10^9/L)<br>Eosinophils (×10^9/L)<br>ESR (mm/h)<br>Volume of blood drawn: at least 2 mL.	Day 1 of Week 16<br>Day 1 of Week 21<br>Day 1 of Week 24<br>Day 1 of Week 37<br>Day 1 of Week 49<br>Day 1 of Week 55	
Blood biochemistry	Glucose<br>Total bilirubin<br>ALT<br>AST<br>GGT<br>Alkaline Phosphatase<br>Creatinine<br>Volume of blood drawn: at least 4 mL.	× 10:<br>at screening,<br>Day 1 of Week 5<br>Day 1 of Week 9<br>Day 1 of Week 13<br>Day 1 of Week 16<br>Day 1 of Week 21<br>Day 1 of Week 24<br>Day 1 of Week 37<br>Day 1 of Week 49<br>Day 1 of Week 55	Study site or<br>Central Laboratory
Serology[19]	HIV Ag/Ab Combo<br>anti-HCV<br>HBsAg<br>anti-HbCor (total IgM and IgG)<br>Microprecipitaion + direct hemagglutination to *T. pallidum*<br>Volume of blood drawn: at least 12 mL.<br><br>Additional examinations[20]:<br>Qualitative PCR for HCV RNA<br>Qualitative PCR for HBV DNA, anti-HbCor IgM and anti-HbCor IgG<br>Volume of blood drawn: at least 10 mL.<br>Specific tests for syphilis: $ELISA_{total}$ or ELISA(IgG)+ELISA(IgM) (blood volume at least 6 mL) or immunofluorescence reaction with absorption (blood volume at least 6 mL) or the T. pallidum immobilization test (blood volume at least 6 mL)<br>Non-specific tests for syphilis: Repeated microprecipitation assay (blood volume drawn is at least 2 mL) or VDRL (blood volume drawn is at least 2 mL)	× 1:<br>at screening	Study site<br>or Central Laboratory<br>or Local Laboratory
Urinalysis	General properties (color, clarity, specific gravity, pH, protein, glucose)<br>Urinary sediment microscopy (epithelium, erythrocytes, leukocytes, cylinders, bacteria, salts).	× 3:<br>at screening,<br>Day 1 of Week 24<br>Day 1 of Week 55	Study site<br>rr Central Laboratory
Pregnancy test	Urine HCG (only in women of childbearing potential; not applicable to post-menopausal women).	× 1: at screening	Study site
Instrumental examinations	ECG	× 3:<br>at screening,	Study site or an external clinic

[19] HIV, HBV, HCV, and syphilis test results are considered valid for screening if they were obtained within 1 month before signing the ICF and if the test result fully meets the requirements of this Protocol.
[20] Additional examinations are performed if anti-HCV or anti-HbCor (IgM + IgG) have been detected in blood or in the case of a positive reaction for syphilis (microprecipitation or direct hemagglutination to *T. pallidum*).

Test	Tested variable	Frequency	Where the test is performed
		Day 1 of Week 24<br>Day 1 of Week 55	
	Lung pathology according to[21] the chest X-ray (posterior-anterior projection) or fluorography	× 3:<br>at screening,<br>Day 1 of Week 25<br>Day 1 of Week 55	Study site or an external clinic
Diaskintest® / QuantiFERON test / T-Spot.TB	Appearance and size of the plaque at the injection size / QuantiFERON test results / T-Spot.TB test results	× 3:<br>at screening,<br>Day 1 of Week 25<br>Day 1 of Week 55	Study site or Central Laboratory
Psoriasis Area and Severity Index (PASI)	Total score showing the severity and area of psoriasis	× 13:<br>at screening,<br>Day 1 of Week 0<br>Day 1 of Week 5<br>Day 1 of Week 9<br>Day 1 of Week 13<br>Day 1 of Week 16<br>Day 1 of Week 21<br>Day 1 of Week 24<br>Day 1 of Week 29<br>Day 1 of Week 33<br>Day 1 of Week 37<br>Day 1 of Week 45<br>Day 1 of Week 55	Study site
BSA	% Affected area		
sPGA	Severity of psoriasis symptoms		
Nail Psoriasis Severity Index (NAPSI)	Score for nail matrix and nail bed involvement	× 5:<br>at screening,<br>Day 1 of Week 16<br>Day 1 of Week 24<br>Day 1 of Week 33<br>Day 1 of Week 55	Study site
Itch severity (VAS)	0 to 100 mm VAS, where 0 refers to no itch and 100 refers to unbearable itch		
PK assessment (in a limited population)	Adalimumab concentration in the serum (blood volume: 6 mL).	× 24:<br>Day 1 of Week 0 (PK0)<br>Day 1 of Week 1 (PK1)<br>Day 1 of Week 3 (PK2)<br>Day 1 of Week 5 (PK3)<br>Day 1 of Week 7 (PK4)<br>Day 1 of Week 9 (PK5)<br>Day 1 of Week 11 (PK6)<br>Day 1 of Week 13 (PK7)<br>Day 1 of Week 15, 15 min ± 5 min before the 9th injection of adalimumab (PK8),<br>Day 1 of Week 15, 3 h ± 15 min after the 9th injection of adalimumab (PK9),<br>Day 1 of Week 15, 12 h ± 30 min after the 9th injection of adalimumab (PK10),	Separate subdivision of JSC BIOCAD (Central Laboratory)

[21] At screening, fluorography may be done instead of the chest X-ray. Screening chest X-ray is not required if the patient has results of the chest X-ray or fluorography or CT or MRI performed within 1 month before signing the informed consent form.

Test	Tested variable	Frequency	Where the test is performed
		Day 2 of Week 15, 24 h ± 30 min after the 9th injection of adalimumab (PK11),<br>Day 3 of Week 15, 48 h ± 90 min after the 9th injection of adalimumab (PK12),<br>Day 4 of Week 15, 72 h ± 120 min after the 9th injection of adalimumab (PK13),<br>Day 5 of Week 15, 96 h ± 120 min after the 9th injection of adalimumab (PK14),<br>Day 6 of Week 15, 120 h ± 120 min after the 9th injection of adalimumab (PK15),<br>Day 7 of Week 15, 144 h ± 120 min after the 9th injection of adalimumab (PK16),<br>Day 2 of Week 16, 192 h ± 120 min after the 9th injection of adalimumab (PK17),<br>Day 4 of Week 16, 240 h ± 120 min after the 9th injection of adalimumab (PK18),<br>Day 6 of Week 16, 288 h ± 120 min after the 9th injection of adalimumab (PK19),<br>Day 1 of Week 17, 15 min before the injection of adalimumab (PK20),<br>Day 1 of Week 19, 15 min before the injection of adalimumab (PK21),<br>Day 1 of Week 21, 15 min before the injection of adalimumab (PK22),<br>Day 1 of Week 23, 15 min before the injection of adalimumab (PK23),	
Immunogenicity assessment	Binding / neutralizing anti-adalimumab antibodies in the serum (blood volume: 8 mL).	× 4:<br>Day 1 of Week 0<br>Day 1 of Week 16<br>Day 1 of Week 33<br>Day 1 of Week 55	JSC BIOCAD, separate subdivision<br>Central Laboratory
Quality of life	DLQI and SF-36 questionnaires	× 5:<br>Day 1 of Week 0<br>Day 1 of Week 16<br>Day 1 of Week 24	Study site

Test	Tested variable	Frequency	Where the test is performed
		Day 1 of Week 33<br>Day 1 of Week 55	

Table 14 shows the estimated volume of blood to be taken on each visit and over the entire study.

Table 20. Volume of blood to be taken from each subject in the study

Visit	Laboratory tests (frequency / blood taken per 1 sample)	Total blood volume
Screening	CBC (1 / 2 mL)<br>Biochemistry (1 / 4 mL)<br>Blood tests for hepatitis, HIV and syphilis (1 / 12 mL)<br>If the QuantiFERON assay is done (4 / 1 mL); if the T-Spot.TB is done (1 / 6 mL)<br>Blood sampling can be repeated once if the specimen is lots or spoiled.	At least 18[22] mL (if the Diaskintest is done)<br>22 mL (if the QuantiFERON assay is performed) or 24 mL (if the T-Spot.TB test is performed)
Week 0 / Day 1	PK (1 / 6 mL)<br>IG (1 / 8 mL)	8 mL or<br>14 mL (patients from the PK population)
Week 1 / Day 1	PK (1 / 6 mL)	6 mL (patients from the PK population)
Week 3 / Day 1	PK (1 / 6 mL)	6 mL (patients from the PK population)
Week 5 / Day 1	CBC (1 / 2 mL)<br>Biochemistry (1 / 4 mL)<br>PK (1 / 6 mL)	6 mL or<br>12 mL (patients from the PK population)
Week 7 / Day 1	PK (1 / 6 mL)	6 mL (patients from the PK population)
Week 9 / Day 1	CBC (1 / 2 mL)<br>Biochemistry (1 / 4 mL)<br>PK (1 / 6 mL)	6 mL or<br>12 mL (patients from the PK population)
Week 11 / Day 1	PK (1 / 6 mL)	6 mL (patients from the PK population)
Week 13 / Day 1	CBC (1 / 2 mL)<br>Biochemistry (1 / 4 mL)<br>PK (1 / 6 mL)	6 mL or<br>12 mL (patients from the PK population)
Week 15 / Day 1	PK (3 / 6 mL)	18 mL (patients from the PK population)
Week 15 / Day 2	PK (1 / 6 mL)	6 mL (patients from the PK population)
Week 15 / Day 3	PK (1 / 6 mL)	6 mL (patients from the PK population)
Week 15 / Day 4	PK (1 / 6 mL)	6 mL (patients from the PK population)
Week 15 / Day 5	PK (1 / 6 mL)	6 mL (patients from the PK population)
Week 15 / Day 6	PK (1 / 6 mL)	6 mL (patients from the PK population)
Week 15 / Day 7	PK (1 / 6 mL)	6 mL (patients from the PK population)

[22] CBC and blood biochemistry can be repeated once. Additional tests for syphilis can be performed. In this case, the total blood volume will be more than 18 mL (depending on the list of tests performed).

Visit	Laboratory tests (frequency / blood taken per 1 sample)	Total blood volume
Week 16 / Day 1	CBC (1 / 2 mL)<br>Biochemistry (1 / 4 mL)<br>IG (1 / 8 mL)	14 mL
Week 16 / Day 2	PK (1 / 6 mL)	6 mL (patients from the PK population)
Week 16 / Day 4	PK (1 / 6 mL)	6 mL (patients from the PK population)
Week 16 / Day 6	PK (1 / 6 mL)	6 mL (patients from the PK population)
Week 17 / Day 1	PK (1 / 6 mL)	6 mL (patients from the PK population)
Week 19 / Day 1	PK (1 / 6 mL)	6 mL (patients from the PK population)
Week 21 / Day 1	CBC (1 / 2 mL)<br>Biochemistry (1 / 4 mL)<br>PK (1 / 6 mL)	6 mL or<br>12 mL (patients from the PK population)
Week 23 / Day 1	PK (1 / 6 mL)	6 mL (patients from the PK population)
Week 24 / Day 1	CBC (1 / 2 mL)<br>Biochemistry (1 / 4 mL)	6 mL
Week 25 / Day 1	If the QuantiFERON assay is done (4 / 1 mL); if the T-Spot.TB is done (1 / 6 mL)	4 mL (if the QuantiFERON assay is performed) or 6 mL (if the T-Spot.TB test is performed) No blood is drawn for the Diaskintest.
Week 33 / Day 1	IG (1 / 8 mL)	8 mL
Week 37 / Day 1	CBC (1 / 2 mL)<br>Biochemistry (1 / 4 mL)	6 mL
Week 49 / Day 1	CBC (1 / 2 mL)<br>Biochemistry (1 / 4 mL)	6 mL
Week 55 / Day 1	CBC (1 / 2 mL)<br>Biochemistry (1 / 4 mL)<br>IG (1 / 8 mL)<br>If the QuantiFERON assay is done (4 / 1 mL); if the T-Spot.TB is done (1 / 6 mL)	14 mL (if the Diaskintest is performed), 18 mL (if the QuantiFERON assay is performed) or 20 mL (if the T-Spot.TB test is performed)
Total blood volume collected from one patient over the study (does not include the volume of blood taken for re-testing, if any)		104 mL (116 mL / 122 mL if the QuantiFERON / T-Spot.TB test is performed) or 248 mL in patients from the PK population (260 mL /266 mL if the QuantiFERON / T-Spot.TB test is performed).

4.7.1. History taking, complaints, demographics

The following parameters will be documented based on the patient’s medical records and questioning:

- Date of birth
- Gender
- Age
- Ethnic origin
- Reproductive potential (use of contraception with methods specified, menopause and its duration, sterilization, if applicable0

- Prior and/or concurrent diseases (infections, chronic infections and inflammatory diseases, tuberculosis, contacts with a tuberculosis patient, immune deficiency, concurrent conditions, allergic reactions) with dates of onset/resolution (if applicable).
- Psoriasis history (approximate date of onset, approximate date of diagnosis, number and duration of relapses and remissions, triggering factors, insolation).
- Medication history:

A) Specify all medications that the patient has received for the treatment of psoriasis, with doses, dosing regimens, therapy duration, and effectiveness of each medication as judged by the investigator. If the medication was discontinued, specify the reason. To assess whether the patient is eligible for the study, a history of the following medications should be elucidated:

- Systemic oral anti-infectious agents within 28 days before signing the informed consent form.
- Systemic IV and/or IM anti-infectious agents within 8 weeks before signing the informed consent form.
- Adalimumab or any other inhibitors of tumor necrosis factor alpha.
- Two or more biologics for the treatment of psoriasis.
- Systemic glucocorticoids, systemic retinoids (acitretin) within 4 weeks before signing the informed consent form.
- Systemic non-biologic medications including (but not limited to) methotrexate, sulphasalazine, cyclosporine, apremilast, mycophenolate mofetil, etc. within 4 weeks before signing the informed consent form; OR leflunomide or cyclosporine within 6 months before signing the informed consent form.
- Phototherapy [including selective phototherapy (UVB) and photochemotherapy (PUVA)] within 4 weeks before signing the informed consent form,
- Vaccination with live or attenuated vaccines within 8 weeks before signing the informed consent form.

B) Specify any medications that the patient has received within 30 days before screening. Specify the dose, dosage regimen, treatment duration, and reason for discontinuation (if applicable).

4.7.2. Physical examination

The following organs and systems must be assessed during the physical exam:

- Height (at screening only)
- Body weight (at screening only)
- Nervous system (abnormalities, if any)
- Skin, mucosal membranes (visual examination)

- Respiratory system (auscultation of lungs)
- Cardiovascular system (auscultation of heart)
- GI tract (abdominal palpation)
- Liver and spleen size (palpation)
- Bowel and bladder habits (normal/abnormalities).

4.7.3. Vital signs

Vital signs include axillary body temperature (ºC), blood pressure (on one arm, mm Hg), and heart rate (wrist pulse, bpm).

4.7.4. Laboratory tests

Laboratory tests include CBC, blood biochemistry, and urinalysis. In addition, the screening examination includes QuantiFERON or T-Spot.TB test and tests for HIV, HBV, HCV, and syphilis.

4.7.4.1. CBC

CBC is tested in accordance with the standard procedure. The test is performed at fasting (fasting means at least 8 hours after the last meal). In this study, the following CBC parameters will be tested:

- Hemoglobin (g/L)
- Hematocrit (%)
- RBC ($\times 10^{12}$/L)
- WBC ($\times 10^{9}$/L)
- Platelets ($\times 10^{9}$/L)
- Neutrophils ($\times 10^{9}$/L)
- Lymphocytes ($\times 10^{9}$/L)
- Eosinophils (x10^{9}/L)
- ESR (mm/h)

Volume of blood taken: at least **2 mL**. At screening, CBC can be once re-tested. If so, the volume of blood taken will be at least 4 mL. On other visits, no re-tests are allowed.

Blood sampling is performed using standard procedures.

4.7.4.2. Blood biochemistry

Blood biochemistry is performed according to the standard procedure at a fasting state (8 hours after the last meal including any sweat or alcohol drinks).

The following variables are to be evaluated:

- Glucose (mmol/L)
- ALT (U/L)
- AST (U/L)
- GGT (U/L)
- Alkaline phosphatase (U/L)
- Bilirubin total (μmol/L)
- Creatinine (μmol/L)

Volume of blood taken: at least **4 mL**. At screening, blood biochemistry can be once re-tested. If so, the volume of blood taken will be at least 8 mL. On other visits, no re-tests are allowed.

Blood sampling is performed using standard procedures.

4.7.4.3. Serology

The test for HIV infection is a qualitative assessment for HIV p24 antigen and HIV Ag/Ab Combo in the serum or plasma.

The screening examination will include tests for HBsAg and total antibodies to HBcor antigen (IgG+IgM). If the patient tests negative for these markers (HBsAg and anti-HBcor total), he/she is considered eligible for the study with respect to this criterion. If the patient tests positive for HBsAg, the patient cannot be included in the study regardless of the results for anti-HBcor. If the patient tests negative for HBsAg but positive for anti-HBcore-total, the patient has to undergo additional examinations. Additional examinations include but were not limited to the following: qualitative PCR for HBV DNA, anti-HBcor-IgG, anti-HBcor-IgM, blood biochemistry, and a consultation with an infectious disease specialist. Having considered the examinations and test results, the Sponsor decides whether to approve such a patient for the study.

The screening examination includes a test for anti-HCV antibodies in the blood. If the patient tested negative for anti-HCV, he/she is considered eligible for the study with respect to this criterion. If the patient tests positive for anti-HCV, the patient has to undergo additional examinations. Additional examinations include but were not limited to the following: qualitative PCR for HCV RNA, blood biochemistry, and consultation with an infectious disease specialist. Having considered the examinations and test results, the Sponsor decides whether to approve such a patient for the study.

The screening will also include syphilis diagnostics. If the patient shows negative results of the microprecipitation reaction and direct hemagglutination to *T. pallidum*, he/she is considered eligible for the study by this criterion. If results of any of the two assays are positive, the patient has to undergo additional examinations. In the case of a positive *T. pallidum* hemagglutination test and negative microprecipitation test, additional examinations include as follows:

- If the patient has a history of syphilis, appropriate medical records should be provided confirming the adequate therapy for syphilis.
- ELISA [ELISA$_{total}$ or ELISA(IgG)+ELISA(IgM)], or immunofluorescence reaction with absorption, or *T. pallidum* immobilization test.
- The dermatology/venereology specialist confirms that the patient has or has no syphilis.

If the *T. pallidum* hemagglutination test is negative and the microprecipitation test is positive, further examinations include as follows:

- Repeated microprecipitation (or VDRL) and a specific assay should be performed: immunofluorescence assay or ELISA (ELISA$_{total}$ or ELISA(IgG)+ELISA (IgM)).
- The dermatology/venereology specialist confirms that the patient has or has no syphilis.

Having considered the report from the dermatology/venereology specialist and the results of additional examinations, the Sponsor decides whether to include this patient in the study.

All these tests are performed using routine procedures of the study site or Central Laboratory. The patient should fast for 8 hours before blood sampling.

Volume of blood taken: **12 mL**. If additional tests are performed, more blood will be taken from the patient.

Serologic tests will be performed once at screening. One re-testing is allowed at screening if the biospecimen is lost or spoiled during the transportation to the Central Laboratory.

4.7.4.4. Urinalysis

Urine sampling and urinalysis will be performed using standard procedures. Urinalysis includes testing of the general urine properties (color, clarity, specific gravity, pH, protein, glucose) and urinary sediment microscopy (epithelium, erythrocytes, leukocytes, cylinders, bacteria, salts).

Frequency and timing:

Urine samples will be taken at screening, at Week 24, and Week 55. Urinalysis should be performed with the mid-stream sample of the first morning urine.

4.7.5. ECG

A 12-lead ECG is recorded according to the standard procedure.

Frequency and timing:

ECG will be performed at screening, at Week 24, and Week 55.

4.7.6. Tests for tuberculosis

In this study, results of the Diaskintest® or QuantiFERON or T-Spot.TB assays obtained with standard procedures will be accepted. Only Diaskintest® or QuantiFERON or T-Spot.TB test results obtained at screening will be accepted for the screening purpose. The Diaskintest® or QuantiFERON or T-Spot.TB test and the test interpretation should be performed by a certified healthcare professional.

Diaskintest® is an intra-skin diagnostic test with a recombinant protein containing two antigens (ESAT6 and CFP10) of *Micobacterium tuberculosis* and *Micobacterium bovis,* virulent strains of mycobacteria. The procedure and of Diaskintest® and results assessment are similar to those of the Mantoux test (PPD-L). The test is done with a thin needle, intradermally, into the middle third of the antebrachium. After 72 hours, the physician or nurse evaluates the response by measuring the transverse (with reference to the brachium axis) size of hyperemia and infiltrate (papula) in mm with a transparent ruler. Hyperemia is taken into account only if no infiltrate is seen. The following responses for the test can be seen (source http://www.diaskintest.ru/page_2.html):

- Negative: no infiltrate or hyperemia. A "prick reaction" of up to 2 mm may occur.
- Indeterminant: hyperemia with no infiltrate.
- Positive: an infiltrate (papula) of any size.

The QuantiFERON test allows evaluating plasma levels of the specific gamma interferon to confirm or exclude the tuberculosis infection.

Procedure of the QuantiFERON test is as follows:

Blood should be collected in the morning. Patients must be fasting (at least 8 hours after the last food intake). Blood samples will be collected into three vacuum test tubes from a QuantiFERON Assay Kit (positive control, negative control, test), with 1 mL of blood transferred in each tube. Remove the tube from the syringe 2 to 3 sec after the blood has reached a black mark on the edge of the label (a measure showing 1 mL of blood). If the blood does not reach the mark, take a new blood sample. The tubes with the blood must be kept at room temperature (+17 °C to +25 °C). Do not refrigerate or freeze the blood samples. Start incubating the tubes within 10 hours after the blood was collected.

Shake well the tubes after blood sampling. **NB! Shake each tube 10 times for 5 seconds so that the internal surface of the tube is coated with blood.**

Incubate the tubes for 16-24 h in a vertical position at 37 °C. During the incubation, lymphocytes recognize mycobacterial antigens. This recognition process includes the production of gamma interferon. If the tubes are not incubated shortly after blood collection, shake them again 10 times before incubation. If the tubes are shaken well, blood foams. This does not affect the test results

and must be seen if the assay is correct. Document the start and end of the incubation in the *BCD-057-2 Incubation Log*, which is attached to the Investigator's Guide provided by the Central Laboratory.

When the incubation is complete, spin the tubes in a centrifuge at 2000-3000 g for 15 min. After spinning, plasma should be clear and red (but not dark red). If plasma does not look correct, re-spin the tubes in the centrifuge at a higher rate.

Before the delivery service arrives, store the tubes chilled in a bag labeled "QuantiFERON Assay Tubes Kit" (+2…+8 °C).

The biospecimens must be sent to the Central Laboratory on the day when the incubation is completed. The tubes must be shipped in a vertical position.

The Central Laboratory uses ELISA assay to test plasma for gamma interferon, which is formed as an immune response to the antigens.

Use only tubes from the QuantiFERON Assay Kit for the QuantiFERON test because each of its tube contains a specific antigen. Do not use any tubes of the same colors but not from the QuantiFERON Assay Kit.

Interpretation of QuantiFERON test results. There are three possible results: Positive, negative, and indeterminant. Indeterminant results may occur due to the sensitivity to the tuberculosis antigen.

The T-Spot.TB is an immunobiologic diagnostic test for tuberculosis. It is based on counting the T cells that have been specifically activated by Mycobacterium tuberculosis.

The patient must be fasting before providing a blood sample for T-Spot.TB test. The sample should be collected in sodium or lithium heparin.

Volume of blood drawn: at least 6 mL.

Rotate the tube upside down 3-4 times immediately after sampling. Do not shake the tube and avoid foaming.

Before delivered to the laboratory for testing, blood samples (no preservatives added) can be stored for not more than 30 hours in a vertical position at a temperature from 18ºC to 25ºC. Do not refrigerate or freeze the biospecimens.

Patients with indeterminant and/or positive results of the QuantiFERON/Diaskintest® are allowed in the study only if all of the following three criteria are met:

- The patient tested negative in one additional QuantiFERON assay.
- No signs of active tuberculosis are seen on the chest X-ray (or fluorography/CT/MRI) done any time within 1 month before signing the informed consent form.
- The TB specialist's report that the patient has no tuberculosis; the report should be given during the screening period.

The patient who has indeterminant or positive results of the QuantiFERON assay or T-Spot.TB test or Diaskintest® at Week 25 is allowed to continue the study only if all three requirements are met:

- The patient tests negative in one additional QuantiFERON assay.
- The chest X-ray shows no signs of active tuberculosis.
- The TB specialist confirms that the patient has no tuberculosis.

Otherwise, the patient has to be removed from the study.

Frequency and timing:

The test will be performed at screening, at Week 25, and at Week 55.

4.7.7. Chest x-ray and fluorography

Chest X-ray is performed to rule out pulmonary tuberculosis. The examination is conducted according to the site's standard procedure. Images are taken in the front view. If necessary, the Sponsor's representative may request the images (if the subject develops active TB while in the study).

Frequency and timing:

The test will be performed at screening, at Week 25, and at Week 55. At screening, fluorography may be done instead of the chest X-ray. Screening chest X-ray is not required if the patient has results of the chest X-ray or fluorography or CT or MRI performed within 1 month before signing the informed consent form.

4.7.8. PASI and BSA assessment

The **PASI** allows evaluating the extent and severity of skin symptoms of psoriasis. Table 15 presents the worksheet used for PASI scoring.

Table 21. Psoriasis area and severity index (PASI). Lesion score: 0 - no symptoms; 1 – mild; 2 – moderate; 3 – severe; 4 – very severe

1		Head and neck	Trunk	Upper limbs	Lower limbs
	Erythema	0<br>1<br>2<br>3<br>4	0<br>1<br>2<br>3<br>4	0<br>1<br>2<br>3<br>4	0<br>1<br>2<br>3<br>4
2		Head and neck	Trunk	Upper limbs	Lower limbs
	Induration	0<br>1<br>2<br>3<br>4	0<br>1<br>2<br>3<br>4	0<br>1<br>2<br>3<br>4	0<br>1<br>2<br>3<br>4

3	Scaling	Head and neck	Trunk	Upper limbs	Lower limbs
		0<br>1<br>2<br>3<br>4	0<br>1<br>2<br>3<br>4	0<br>1<br>2<br>3<br>4	0<br>1<br>2<br>3<br>4
4	Total				

Table 22. Percentage area affected, %

5	0 refers to no symptoms; 1 is < 10%; 2 is from 10% to 29%; 3 is from 30% to 49%; 4 is from 50% to 69%; 5 is from 70% to 89%; 6 is from 90% to 100%				
6	Area score				
7	Multiply the value in line 4 by the value in line 6				
8	Body surface area	×0.1	×0.3	×0.2	×0.4
9	Multiply the value in line 7 by the value in line 8				
Total value (add together each of the scores for each body region)					

Each symptom (erythema, induration, and scaling) is assessed with a 4-point scale from 0 (none) to 4 (very severe), separately for the skin of the head and neck, body, upper and lower limbs (lines 1-3). The assessment was performed for the area mostly affected by the evaluated symptom. The scores were summarized (line 4).

The BSA affected by psoriasis is then estimated for each anatomical region. The BSA is estimated with the palm method. One percent of BSA is approximately equal to the palm of the patient’s hand excluding fingers. It is assumed that the skin of the head and neck corresponds to 10% of all skin area, trunk – to 30%, upper limbs – to 20%, and lower limbs – to 40%. Thus, one palm makes up 10% of the scalp skin, 3.3% of trunk skin, 5% of the skin of the upper limbs, and 2.5% of the skin of the lower limbs.

To estimate the BSA score for each section, the affected area (%) from each section is matched to the values from line 5, and the score was entered to line 6.

After that, the value in line 7 is multiplied by the weight of the respective section (0.1 for the head, 0.2 for upper limbs, 0.3 for the trunk, and 0.4 for the lower limbs).

The values in line 9 are then summed up to obtain the PASI score, which can vary from 0 (no rashes) to 72 (the most severe psoriatic process).

The PASI score is calculated automatically when the investigator enters the following data in the eCRF: severity of erythema, induration, and scaling by sections and the BSA. The total PASI score and severity of individual symptoms by anatomical regions (lines 1-4 of Table 15) must be recorded in source documents

The investigator determines the BSA during the study visits and records it in source documents and eCRF as a total body surface affected by psoriasis (%). Assessment is performed with the “palm” method.

Frequency and timing:

The PASI and BSA are determined at screening, at Visit 1 (Week 0), Visit 4 (Week 5), Visit 6 (Week 9), Visit 8 (Week 13), Visit 10 (Week 16), Visit 13 (Week 21), Visit 15 (Week 24), Visit 18 (Week 29), Visit 20 (Week 33), Visit 22 (Week 37), Visit 26 (Week 45), and Visit 30 (Week 55).

4.7.9. Static Physician Global Assessment (sPGA)

The clinical course of psoriasis is assessed with the sPGA scale. The investigator chooses the sPGA item that best describes the patient’s symptoms. The scale is shown in Appendix 3.

The scale includes three criteria (induration, scaling, and erythema). Not all three criteria may be present. Induration is considered the most resistant symptom, while the presence of scaling or erythema can vary. In this case, the index is a sum of the scores for induration in the lesions and one of the prevailing criteria (erythema or scaling).

The investigator determines sPGA score at each visit and records it in the source documents and eCRF.

Frequency and timing:

The sPGA score is determined at screening, at Visit 1 (Week 0), Visit 4 (Week 5), Visit 6 (Week 9), Visit 8 (Week 13), Visit 10 (Week 16), Visit 13 (Week 21), Visit 15 (Week 24), Visit 18 (Week 29), Visit 20 (Week 33), Visit 22 (Week 37), Visit 26 (Week 45), and Visit 30 (Week 55).

4.7.10. Nail psoriasis severity index (NAPSI)

The investigator evaluates the matrix involvement (pitting, leukonychia, red spots in the lunula, and nail plate crumbling) and/or nail bed involvement (onycholysis, nail bed hyperkeratosis, hemorrhages, and “oil drops”). The most affected nail is divided into quadrants. In each quadrant, the severity of nail matrix and nail bed involvement is given a score from 0 to 4. The sum of these two scores (symptoms of nail bed and nail matrix involvement) is the total NAPSI score.

The higher is the NAPSI, the more severe are the nail changes due to psoriasis. The maximum score (8 points) refers to the total involvement of the fingernail.

Table 23. Nail psoriasis severity index (NAPSI).

**Right hand:** Nail matrix involvement<br>*(pitting, leukonychia, red spots, crumbling)*<br>0 — no symptoms, 1 — symptoms are present in one nail quadrant, 2 — symptoms are present in two nail quadrants, 3 — symptoms are present in three nail quadrants, 4 — symptoms are present in four nail quadrants.

Nail of the little finger	0 □ 1 □ 2 □ 3 □ 4□
Nail of the ring finger	0 □ 1 □ 2 □ 3 □ 4□
Nail of the third finger	0 □ 1 □ 2 □ 3 □ 4□
Nail of the index finger	0 □ 1 □ 2 □ 3 □ 4□
Nail of the thumb	0 □ 1 □ 2 □ 3 □ 4□
**Right hand:** Nail bed involvement<br>*(onycholysis, subungual hyperkeratosis, hemorrhages, "oil drops")*<br>0 — no symptoms, 1 — symptoms are present in one nail quadrant, 2 — symptoms are present in two nail quadrants, 3 — symptoms are present in three nail quadrants, 4 — symptoms are present in four nail quadrants.	
Nail of the little finger	0 □ 1 □ 2 □ 3 □ 4□
Nail of the ring finger	0 □ 1 □ 2 □ 3 □ 4□
Nail of the third finger	0 □ 1 □ 2 □ 3 □ 4□
Nail of the index finger	0 □ 1 □ 2 □ 3 □ 4□
Nail of the thumb	0 □ 1 □ 2 □ 3 □ 4□
**Left hand:** Nail matrix involvement<br>*(pitting, leukonychia, red spots, crumbling)*<br>0 — no symptoms, 1 — symptoms are present in one nail quadrant, 2 — symptoms are present in two nail quadrants, 3 — symptoms are present in three nail quadrants, 4 — symptoms are present in four nail quadrants.	
Nail of the little finger	0 □ 1 □ 2 □ 3 □ 4□
Nail of the ring finger	0 □ 1 □ 2 □ 3 □ 4□
Nail of the third finger	0 □ 1 □ 2 □ 3 □ 4□
Nail of the index finger	0 □ 1 □ 2 □ 3 □ 4□
Nail of the thumb	0 □ 1 □ 2 □ 3 □ 4□
**Left hand:** Nail bed involvement<br>*(onycholysis, subungual hyperkeratosis, hemorrhages, "oil drops")*<br>0 — no symptoms, 1 — symptoms are present in one nail quadrant, 2 — symptoms are present in two nail quadrants, 3 — symptoms are present in three nail quadrants, 4 — symptoms are present in four nail quadrants.	
Nail of the little finger	0 □ 1 □ 2 □ 3 □ 4□
Nail of the ring finger	0 □ 1 □ 2 □ 3 □ 4□
Nail of the third finger	0 □ 1 □ 2 □ 3 □ 4□
Nail of the index finger	0 □ 1 □ 2 □ 3 □ 4□
Nail of the thumb	0 □ 1 □ 2 □ 3 □ 4□

The NAPSI is from 0 to 8 for each nail. All 8 components can be assessed for one target nail (in this case, the index will be from 0 to 32). In this study, the nail involvement will be assessed only for hands, so the total NAPSI for all fingernails can be from 0 to 80 (hands only). The investigator should perform a complete assessment of hand NAPSI. The questionnaires will be provided to document the scores for each fingernail. The total score is calculated automatically when data from the questionnaire are entered in the eCRF. The score should also be recorded in the source documents.

The test is performed at screening, Visit 10 (Week 16), Visit 15 (Week 24), Visit 20 (Week 33), and Visit 30 (Week 55).

4.7.11. Patient assessment of itching

The patient will assess the itch severity with a visual analog scale ranging from 0 (no itch) to 100 mm (unbearable itch). It is required that the patient fills out the scale while at the study site at the day of the visit. The completed VAS should be stored in the Investigator's File. The score is to be recorded in the source documents. The investigator enters the VAS score in the eCRF.

Figure 5. An example of the visual analog scale for itch assessment

How severe is an itch you have now due to psoriasis?

No itch ——————————————————— 100 Unbearable itch

The test is performed at screening, Visit 10 (Week 16), Visit 15 (Week 24), Visit 20 (Week 33), and Visit 30 (Week 55).

4.7.12. Quality of life assessment

The quality of life in the study subjects will be assessed with the DLQI and SF-36 questionnaires (in Russian for Russian-speaking patients and in English for all other patients).

The patient should fill out the questionnaires during the visit at the study site. The investigator must ensure that the patient fills out the questionnaire at the beginning of the visit before injection of the study drug (if the injection is scheduled for this certain visit).

In the DLQI survey, each question is given a score from 0 to 3. Score 3 means “very much / very often”, score 2 means “a lot / often”, score 1 means “a little”, and score 0 means “not at all / do not know”. For question 7, the answer “yes” gives 3 points and the answer “not at all” gives 0. If more than two questions are left unanswered, the questionnaire is considered invalid. The DLQI is calculated by summing the score of each question resulting in a maximum of 30 and a minimum of 0. The higher the score, the more quality of life is impaired.

The SF-36 questionnaire is filled out using a special worksheet. The results are transferred to the eCRF.

Templates of the questionnaires are given in Appendices 1 and 2. The questionnaire must be filled out only by the patient or his/her legal representative. DLQI and SF-36 questionnaires must not be given to patients for completing at home. These questionnaires must by filled at the study site on the day of the visit. Completed questionnaires must be stored in the Investigator’s File.

The DLQI and SF-36 scores are calculated automatically when the data are entered in the eCRF. The DLQI and SF-36 scores must be recorded in the source documents.

Frequency and timing:

The QoL questionnaires will be filled out at Visit 1 (Week 0), Visit 10 (Week 16), Visit 15 (Week 24), Visit 20 (Week 33), and Visit 30 (Week 55).

4.7.13. Collecting blood samples for PK assessment

[redacted]

[redacted]

[redacted]

[redacted]	[redacted]	[redacted]	[redacted]	[redacted]	[redacted]	[redacted]
[redacted]	[redacted]	[redacted]	[redacted]	[redacted]	[redacted]	[redacted]
[redacted]	[redacted]	[redacted]	[redacted]	[redacted]	[redacted]	[redacted]
[redacted]	[redacted]	[redacted]	[redacted]	[redacted]	[redacted]	[redacted]
[redacted]	[redacted]	[redacted]	[redacted]	[redacted]	[redacted]	[redacted]
[redacted]	[redacted]	[redacted]	[redacted]	[redacted]	[redacted]	[redacted]
[redacted]	[redacted]	[redacted]	[redacted]	[redacted]	[redacted]	[redacted]
[redacted]	[redacted]	[redacted]	[redacted]	[redacted]	[redacted]	[redacted]
[redacted]	[redacted]	[redacted]	[redacted]	[redacted]	[redacted]	[redacted]
[redacted]	[redacted]	[redacted]	[redacted]	[redacted]	[redacted]	[redacted]
[redacted]	[redacted]	[redacted]	[redacted]	[redacted]	[redacted]	[redacted]
[redacted]	[redacted]	[redacted]	[redacted]	[redacted]	[redacted]	[redacted]
[redacted]	[redacted]	[redacted]	[redacted]	[redacted]	[redacted]	[redacted]

[redacted]	[redacted]	[redacted]	[redacted]	[redacted]	[redacted]
[redacted]	[redacted]	[redacted]	[redacted]	[redacted]	[redacted]
[redacted]	[redacted]	[redacted]	[redacted]	[redacted]	[redacted]
[redacted]	[redacted]	[redacted]	[redacted]	[redacted]	[redacted]
[redacted]	[redacted]	[redacted]	[redacted]	[redacted]	[redacted]
[redacted]	[redacted]	[redacted]	[redacted]	[redacted]	[redacted]
[redacted]	[redacted]	[redacted]	[redacted]	[redacted]	[redacted]
[redacted]	[redacted]	[redacted]	[redacted]	[redacted]	[redacted]
[redacted]	[redacted]	[redacted]	[redacted]	[redacted]	[redacted]
[redacted]	[redacted]	[redacted]	[redacted]	[redacted]	[redacted]
[redacted]	[redacted]	[redacted]	[redacted]	[redacted]	[redacted]
[redacted]	[redacted]	[redacted]	[redacted]	[redacted]	[redacted]
[redacted]	[redacted]	[redacted]	[redacted]	[redacted]	[redacted]

[redacted]

4.7.14. Blood sampling for immunogenicity assessment

[redacted]

[redacted]

[redacted]

[redacted]

[redacted]	[redacted]	[redacted]	[redacted]	[redacted]
[redacted]	[redacted]	[redacted]	[redacted]	[redacted]
[redacted]	[redacted]	[redacted]	[redacted]	[redacted]
[redacted]	[redacted]	[redacted]	[redacted]	[redacted]
[redacted]	[redacted]	[redacted]	[redacted]	[redacted]

4.7.15. Storage and shipment of PK and immunogenicity specimens

[redacted]

[redacted]

[redacted]

4.7.16. Adalimumab assay in the serum

[redacted]

4.7.17. Unscheduled visits

At the discretion of the investigator, the patient can come to the study site for an unscheduled visit to undergo additional safety tests (repeated laboratory tests, AE assessment, etc.) or other

additional tests. If such a visit occurs, the investigator must recorded it in the source documents and in an Unscheduled Visit Form in the eCRF, providing the justification of the visit and specifying all the procedures performed.

4.7.18. Data entering to eCRF

The principal investigator and the study team members will be provided logins and passwords to enter the eCRF system.

The eCRF should be completed within 5 working days after the visit. Screening data are recorded in the eCRF all at once after all the screening procedures are completed but before the patient is randomized.

In the IWRS, the investigator should document the receipt of the investigational product and register the study subjects after they have signed their informed consent forms. The investigator enters the screening results into the eCRF and sends the paper copy of the Screening Form to JSC BIOCAD. If JSC BIOCAD approves the patient for the study, the investigator will stratify and randomize the patient with the IWRS, which will assign the patient with the Subject ID and individual lot number of the investigational product. In addition, the IWRS maintains records of the investigational products used after each visit when adalimumab injection is scheduled.

4.8. Stop rules and criteria for premature withdrawal for study subjects, study periods, and study as a whole

4.8.1. Stop rules for study as a whole

The study may be stopped in the following situations:

1. JSC BIOCAD decides to stop the study due to safety reasons, ethical considerations, compliance issues, or any other reasons.
2. Local ethics committees or regulatory authorities decide to stop the study.

4.8.2. Criteria for premature withdrawal of study subjects

The patient will be withdrawn from the study in the following circumstances:

1. A major violation of inclusion/exclusion criteria is revealed after the patient was enrolled in the study (withdrawn by the decision of JSC BIOCAD)[23].
2. The patient recalls his/her consent to participate in the study.
3. The patient develops adverse events or serious adverse events, laboratory abnormalities or comorbidities that, in the investigator's opinion, make further participation in the study

[23] Deviations from inclusion/exclusion criteria justified by the investigator and approved by the Sponsor are not considered a reason for withdrawal.

impossible, dangerous or non-beneficial regarding patient's well-being and/or safety.

4. The patient misses more than 4 injections and/or visits during the study (compliance < 85%) or misses 2 consecutive injections and/or visits, or breaches the specified time windows for more than 5 visits.
5. The patient develops active tuberculosis. If the patient has indeterminant or positive results of the QuantiFERON assay or T-Spot.TB or Diaskintest® at Week 25, he/she can continue the study only if all three of the following requirements are met:
   - The patient tests negative in one additional QuantiFERON / T-Spot.TB assay.
   - The chest X-ray shows no signs of active tuberculosis.
   - The TB specialist confirms that the patient has no tuberculosis.

   Otherwise, the patient has to be removed from the study.
6. The patient gets pregnant. If pregnancy is suspected, the urine test for HCG must be performed. If the test gives a positive result, the patient must be withdrawn from the study (follow-up procedures are described in section 5.4).
7. The patient has a major depressive disorder or suicidal ideation or makes suicidal attempts.
8. The study is terminated by JSC BIOCAD, local ethics committees, or regulatory authorities.
9. The patient uses medications prohibited by the Protocol.
10. The patient dies.

The investigator must inform JSC BIOCAD within 24 h about a patient's premature withdrawal and specify the reason.

If the subject discontinues the study, an Early Withdrawal Form should be filled out in the CRF. The follow-up procedures for the discontinued subjects are described in section 5.4 *Procedures by Visits*.

Patients who discontinued the study due to safety-related reasons will not be replaced.

For further details, refer to section 11.5 *Study termination.*

4.9. Drug accountability

The investigator is responsible for the investigational product accountability at the trial site. Throughout the study, the investigator must ensure proper accountability of the investigational product as required by regulatory authorities. The investigator must maintain accurate records regarding the receipt of the investigational product from JSC BIOCAD and its dispensing to/ return by study subjects.

When the investigator or the pharmacist receives the investigational product, he/she must check the delivery, sign and date a receipt form (2 copies) provided by JSC BIOCAD and return one copy to JSC BIOCAD. Another copy of this documentation must be kept in the Investigator's File.

The delivery should be recorded in the IWRS. The amount of investigational product delivered to the study site must be recorded in the Drug Accountability Log provided by JSC BIOCAD. This Log will be used as an investigational product accountability record.

Accurate accountability records should be available to the CRA during each monitoring visit. Drug accountability records should include:

1. Delivery confirmation,
2. Inventory at the study sit,
3. Use of investigational product by each study subject,
4. Return of unused drug product to JSC BIOCAD.

Records should include dates, amounts, lot numbers, and expiry dates of the investigational products (if applicable). The investigator must maintain accountability records to ensure that:

- Study subjects get the investigational product at doses specified by the Protocol or Protocol Amendment;
- The total amount of the investigational product provided by JSC BIOCAD is accurately checked and delivered undamaged.

Unused investigational product must not be used for any other purpose except this study. The test drug/reference drug must not be distributed to study subjects for self-administration.

The BIOCAD's CRA will regularly collect the Accountability Log for monitoring.

4.9.1. Handling of investigational products

The investigational products should be stored in a refrigerator at a temperature from 2°C to 8°C. The investigational product must be kept in a limited access area. Only the principal investigator, co-investigators, study team nurses, and an authorized representative of the site administration will have access to the storage area.

The investigational product may be stored only at the study sites authorized for this clinical trial. The investigator must ensure safe storage of the investigational drug to prevent loss, theft, or improper environmental conditions (temperature) specified by the Sponsor and described in the Investigator Brochure. The investigator must maintain a temperature log (in-house or provided by the Sponsor).

All injections of BCD-057/Humira® should be performed at the study site and should be given by an authorized member of the study team.

4.10. Procedure for keeping and unblinding randomization codes

As the study is double-blind, the study product and the reference product will be identified only by lot number on the packs. JSC BIOCAD will prepare randomization envelops with the information unblinding the therapy arm codes for each drug lot. The envelopes will be provided to the study site and stored in the Investigator's File or in a limited access place. The investigator is allowed to unblind the treatment only in case of an emergency. The investigator must exercise his/her best efforts to prevent the loss or unblinding of study codes when unnecessary.

The treatment can be unblinded only in case of an emergency, when this is necessary to give an appropriate treatment to the patient and ensure his/her well-being. If a medical situation that requires unblinding occurs, the principal investigator must contact a representative from JSC BIOCAD (medical expert, pharmacovigilance officer, or clinical study manager) specify the reasons for unblinding reasons and obtain an approval for this procedure. All the contact information is presented on the title page of this Protocol. If JSC BIOCAD approves unblinding, the investigator unblinds a study code, records this fact in the source document, and notifies the clinical study manager by phone or e-mail

All patients participating in the study will receive Subject ID cards containing the protocol code, study site number, investigator's contact information, contact information of the BIOCAD's responsible medical officer and pharmacovigilance officer (available 24 h daily). Patients should be aware that they should carry their Subject ID cards all the time and notify medical staff at any other medical facility that they participate in this clinical study. If necessary (for example, to administer another medicinal product), a physician at another medical facility (other than the study center) can contact the investigator to discuss benefits and potential risks related to concomitant therapy.

4.11. Data entered directly into CRF (i.e. no prior written or electronic record of data) and considered as source data

All data entered to the CRF must be recorded in the source documentation.

In this study, no data will be entered directly to the CRFs without prior entering to the source documents.

5. Eligibility and exclusion of study subjects

5.1. Inclusion criteria

1. The patient signed the informed consent form.
2. The patient is 18 to 75 years old at screening.
3. The patient has moderate-to-severe plaque psoriasis and was diagnosed at least 6 months before signing the informed consent form.
4. The patient received at least one course of phototherapy or systemic therapy for psoriasis and, in the opinion of the investigator, responded inadequately, or the patient was a candidate for any of these therapies.
5. The BSA ≥ 10%, PASI ≥ 12, and sPGA ≥ 3 at screening.
6. Laboratory values at screening:
   - Hb ≥ 10 g/dL (100 g/L)
   - WBC count ≥ 3000/μL (3.0×10^9/L)
   - Platelet count ≥ 100 000/μL (100×10^9/L)
   - Neutrophil count ≥ 2000/μL (2×10^9/L)
   - AST, ALT, and alkaline phosphatase ≤ 2.5 × ULN
   - Serum creatinine < 176.8 μmol/L (2.0 mg/dL)
7. The patient tested negative for serum and virologic markers of active and latent hepatitis B (HBV)[24] and hepatitis C (HCV)[25].
8. The patient has a negative urine pregnancy test at screening (the test is not performed for women who are post-menopausal for at least 2 years or those who underwent surgical sterilization).
9. The patient tested negative for tuberculosis:
   - The negative result of the QuantiFERON or T-Spot.TB.

   OR
   - The negative result of the Diaskintest®.
10. Patients with indeterminant and/or positive results of the QuantiFERON/Diaskintest® are allowed in the study only if all of the following three criteria are met:

---

[24] The screening examination includes tests for HBsAg and total antibodies to HBcor antigen (IgG+IgM). If the patient tested negative for these markers (HBsAg and anti-HBcor total), he/she is considered eligible for the study with respect to this criterion. If the patient tested positive for HBsAg, the patient cannot be included in the study regardless of the results for anti-HBcor. If the patient tested negative for HBsAg but positive for anti-HBcore-total, the patient has to undergo additional examinations. Additional examinations include but were not limited to the following: qualitative PCR for HBV DNA, anti-HBcor-IgG, anti-HBcor-IgM, blood biochemistry, and a consultation with an infectious disease specialist. Having considered the examinations and test results, the Sponsor decides whether to approve such a patient for the study.

[25] The screening examination includes a test for anti-HCV antibodies in the blood. If the patient tests negative for anti-HCV, he/she is considered eligible for the study with respect to this criterion. If the patient tests positive for anti-HCV, the patient has to undergo additional examinations. Additional examinations include but were not limited to the following: qualitative PCR for HCV RNA, blood biochemistry, and consultation with an infectious disease specialist. Having considered the examinations and test results, the Sponsor decides whether to approve such a patient for the study.

- The patient tests negative in one additional QuantiFERON / T-Spot.TB assay.
- No signs of active tuberculosis are seen on the chest X-ray (or fluorography or CT or MRI) done any time within 1 month before signing the informed consent form.
- The TB specialist reported that the patient has no tuberculosis. The report has to be obtained during the screening.

11. The investigator considers the patient able to carry out all the procedures per protocol.
12. The patient and his/her sexual partner with retained reproductive potential are ready to implement reliable contraception throughout the screening period (starting from signing the informed consent) and during four weeks after the last dose of BCD-057/Humira®. This requirement does not apply to the patients who had undergone surgical sterilization. Reliable contraception means one barrier method in combination with one of the following: spermicides, intrauterine device and/or oral contraceptives used by the patient or patient's partner.

5.2. Exclusion criteria

1. Baseline erythrodermic or pustular psoriasis or any other skin diseases (e.g. eczema) that can affect and/or complicate the assessment of psoriasis treatment with the study drugs.
2. The patient had been exposed to any monoclonal antibodies inhibiting TNFα.
3. The patient had used two or more monoclonal antibodies (or their fragments) against other targets.
4. Less than 12 weeks have passed between the patient stopped using the monoclonal antibody therapy and signing the informed consent form for this study.
5. The patient had used or now uses any of the following concomitant therapies:
   - Systemic (oral or parenteral) glucocorticoids or systemic retinoids (acitretin) within 4 weeks before the date of signing the informed consent form or during the screening period.
   - Systemic non-biologic medications including (but not limited to) methotrexate, sulphasalazine, cyclosporine, mycophenolate mofetil, apremilast, etc. within 4 weeks before signing the informed consent form; OR leflunomide or cyclosporine within 6 months before signing the informed consent form.
   - Phototherapy [including selective phototherapy (UVB) and photochemotherapy (PUVA)] within 4 weeks before signing the informed consent form.
6. Vaccination with live or attenuated vaccines any time within 8 weeks before signing the informed consent form.

7. The patient had a major surgery[26] within 30 days before signing the informed consent form or a major surgery is planned during the study.
8. The patient has an active infection at screening or has a history of any infections meeting the criteria below:
   - Any active infection that required systemic anti-infectious agents within 28 days before signing the informed consent form.
   - Any infection that required hospitalization or intravenous and/or intramuscular administration of anti-infectious agents within 8 weeks before signing the informed consent form.
   - Recurring, chromic or any other active infection if the investigator believes that the study drug can harm the patient.
9. Documented HIV-infection or a history of a severe immunodeficiency of any origin.
10. HBs antigen, antibodies to HBcor[27] antigen and/or antibodies to hepatitis C virus[28] revealed at screening.
11. Ongoing tuberculosis or a history of tuberculosis.
12. A positive microprecipitation reaction (MPR) together with a positive *T. pallidum* hemagglutination assay (TPHA)[29]) at screening. If results of any of the two assays are positive, the patient has to undergo additional examinations. In the case of a positive *T. pallidum* hemagglutination test and negative microprecipitation test, additional examinations include as follows:
    - If the patient has a history of syphilis, appropriate medical records should be provided confirming the adequate therapy for syphilis.
    - ELISA [$ELISA_{total}$ or ELISA(IgG)+ELISA(IgM)], or immunofluorescence reaction with absorption, or *T. pallidum* immobilization test.
    - The dermatology/venereology specialist confirms that the patient has or has no syphilis.

---

[26] A surgery associated with a high risk of severe blood loss (> 10% of the circulating blood volume) or any other life-threatening event.
[27] The screening examination includes tests for HBsAg and total antibodies to HBcor antigen (IgG+IgM). If the patient tested negative for these markers (HBsAg and anti-HBcor total), he/she is considered eligible for the study with respect to this criterion. If the patient tested positive for HBsAg, the patient cannot be included in the study regardless of the results for anti-HBcor. If the patient tested negative for HBsAg but positive for anti-HBcore-total, the patient has to undergo additional examinations. Additional examinations include but were not limited to the following: qualitative PCR for HBV DNA, anti-HBcor-IgG, anti-HBcor-IgM, blood biochemistry, and a consultation with an infectious disease specialist. Having considered the examinations and test results, the Sponsor decides whether to approve such a patient for the study.
[28] The screening examination includes a test for anti-HCV antibodies in the blood. If the patient tests negative for anti-HCV, he/she is considered eligible for the study with respect to this criterion. If the patient tests positive for anti-HCV, the patient has to undergo additional examinations. Additional examinations include but were not limited to the following: qualitative PCR for HCV RNA, blood biochemistry, and consultation with an infectious disease specialist. Having considered the examinations and test results, the Sponsor decides whether to approve such a patient for the study.
[29] If hemagglutination test cannot be performed, ELISA total or ELISA(IgG)+ELISA(IgM) should be done instead. In the case of positive results, the algorithm is the same as after a positive hemagglutination assay.

- If the *T. pallidum* hemagglutination test is negative and the microprecipitation test is positive, further examinations include as follows:
- Repeated microprecipitation (or VDRL) and a specific assay should be performed: immunofluorescence assay or ELISA ($ELISA_{total}$ or ELISA(IgG)+ELISA (IgM)).
- The dermatology/venereology specialist confirms that the patient has or has no syphilis.
- Having considered the report from the dermatology/venereology specialist and the results of additional examinations, the Sponsor decides whether to include this patient in the study.

13. Baseline concomitant medical conditions that can increase the risk of adverse events during the study therapy, affect the assessment of the severity of psoriasis, mask, aggravate, or alter the symptoms of psoriasis, or result in the same clinical and/or laboratory and/or instrumental symptoms as those of psoriasis:
    - Acute inflammatory diseases or relapses of chronic inflammatory diseases other than psoriasis.
    - Stable angina class III-IV, unstable angina or a history of myocardial infarction within 1 year before signing the informed consent.
    - Moderate to severe cardiac failure (NYHA classes III and IV).
    - Severe treatment-resistant hypertension[30];
    - Atopic asthma and/or a history of angioedema.
    - Moderate to severe respiratory failure and/or COPD grade 3/4.
    - Decompensated diabetes mellitus.
    - Systemic autoimmune diseases (including systemic lupus erythematosus, Crohn’s disease, ulcerative colitis, systemic scleroderma, inflammatory myopathy, mixed forms of inflammatory diseases of the connective tissue, overlap syndrome, etc.).
    - Active neurological diseases, such as multiple sclerosis, Guillain-Barre syndrome, optic nerve neuritis, transverse myelitis, or a history of neurological symptoms suggesting demyelinating diseases of the central nervous system.
    - Any other concurrent diseases (including but not limited to metabolism, blood, kidney, liver, lung, neurological, endocrine, heart, and GI disorders and/or infections) that, in the investigator’s opinion, may affect the course of psoriasis, confound the assessment of its symptoms, or put the study subject at unacceptable risk during the treatment with BCD-057/Humira®.

[30] Resistant hypertension includes all cases of hypertension that are not controlled by the the concurrent use of three anti-hypertensive drugs of different classes, including a diuretic, and non-medication methods (salt-free diet, controlled physical exercise).

14. Malignancies in remission for less than 5 years, except for adequately treated (cured) squamous and basal cell carcinoma, cervical cancer *in situ* or ductal breast carcinoma *in situ*.
15. A history of hypersensitivity to adalimumab or any excipients of Humira® and/or BCD-057 (mannitol, citric acid monohydrate, sodium citrate, sodium hydrogen phosphate dihydrate, sodium dihydrogen phosphate dihydrate, sodium chloride, polysorbate 80, sodium hydroxide).
16. Known allergy to monoclonal antibodies (murine, chimeric, humanized or fully human).
17. Pregnancy, breastfeeding or planning for pregnancy while participating in the study.
18. Any psychiatric disorders, including a history of major depression and/or suicidal thoughts that can, in the investigator’s opinion, put the patient at risk or affect patient’s ability to follow the study protocol.
19. Use of recreational drugs, alcohol and/or medications and/or psychoactive substance abuse problems.
20. Participation in any other clinical study within 3 months before screening or simultaneous participation in other clinical studies[31].
21. Patients who were randomized to this study and then discontinued the study due to any reasons (i.e. patients who met all other inclusion/exclusion criteria), cannot be re-enrolled in this study.

5.3. Withdrawal criteria

The patient will be withdrawn from the study in the following circumstances:

1. Major violations of inclusion and/or exclusion criteria revealed after the enrollment (patients removed at the discretion of JSC BIOCAD)[32].
2. The patient recalls his/her consent to participate in the study.
3. The patient develops adverse events or serious adverse events, laboratory abnormalities or comorbidities that, in the investigator’s opinion, make further participation in the study impossible, dangerous or non-beneficial regarding patient’s well-being and/or safety.
4. The patient misses more than 4 injections and/or visits during the study (compliance < 85%) or misses 2 consecutive injections and/or visits, or breaches the specified time windows for more than 5 visits.
5. The patient develops active tuberculosis. If the patient has indeterminant or positive results of the QuantiFERON assay or T-Spot.TB or Diaskintest® at Week 25, he/she can continue the study only if all three of the following requirements are met:

[31] Patients whose screening was interrupted due to force majeure circumstances (external events beyond the patient’s and investigator’s control) could be re-screened in this study.
[32] Deviations from inclusion and/or exclusion criteria justified by the investigator and approved by the Sponsor are not considered a reason for withdrawal.

- The patient tests negative in one additional QuantiFERON / T-Spot.TB assay.
- The chest X-ray shows no signs of active tuberculosis.
- The TB specialist confirms that the patient has no tuberculosis.
- Otherwise, the patient has to be removed from the study.

6. The patient gets pregnant. If pregnancy is suspected, the urine test for HCG must be performed. If the test gives a positive result, the patient must be withdrawn from the study (follow-up procedures are described in section 5.4).
7. The patient has a major depressive disorder or suicidal ideation or makes suicidal attempts.
8. The study is terminated by the decision of JSC BIOCAD, local ethics committees or regulatory authorities.
9. The patient used medications prohibited by the Protocol.
10. The patient dies.

The investigator must inform JSC BIOCAD within 24 h about a patient's premature withdrawal and specify the reason.

5.4. Follow-up of subjects withdrawn from the study or subjects who discontinued the study treatment but remain in the study for follow-up

5.4.1. Follow-up of patients who received at least one dose of BCD-057/Humira®

The following should be done if the patient who received at least one injection of BCD-057/Humira® discontinues the study early:

- At the withdrawal day, the investigator should perform the Early Termination Visit and fill out the source documents together with the Unscheduled Visit section of the CRF. On this visit, the following procedures should be performed: collecting data on concomitant therapy, physical examination, and measuring blood pressure and body temperature.

The Early Withdrawal visits are mandatory for all subjects who prematurely withdraw from the study, except for the subjects who are lost to follow-up or are physically unable to attend the visit. All records must be supported with appropriate source documents.

If the subject discontinues the study due to an AE/SAE, the investigator should conduct further treatment and follow-up after the Early Withdrawal Visit in accordance with the study site standards for the treatment of a certain AE/SAE. The patient should be followed up until **the AE or SAE resolves completely**. In addition, an Additional Early Termination Visit is performed 28±2 days later (procedures are described above).

The female patient withdrawn from the study due to pregnancy that occurred during the study period should be followed up through the entire pregnancy and for 6 months after delivery to evaluate the mother's and child's health. These follow-up procedures can be performed only if the woman has given her consent. Information on pregnancy course and outcome should be recorded in the source documentation. During the entire period of pregnancy, the investigator in collaboration with the attending Ob/Gyn specialist should monitor the patient's overall health, the course of pregnancy, and laboratory values including ultrasound. When the child is born, the investigator together with the attending pediatrician should monitor the newborn for 6 months evaluating the child's clinical status and the laboratory/instrumental findings.

Information regarding the health state of all dropouts should be recorded in their source documentation and CRFs.

5.4.2. Follow-up of patients who did not receive a single dose of BCD-057/Humira®

If the subject discontinues the study before dosing, the Early Withdrawal Form (eCRF) should be filled out on the day of withdrawal. Non-dosed patients are followed-up only if they discontinue the study because of AEs/SAEs. In this case, the follow up is performed according to the study site standards.

If the subject is withdrawn from the study due to any reason after receiving at least one dose of BCD-057/Humira®, his/her data will be included in the analysis. If the subject is withdrawn from the study before dosing, his/her information will be removed from the analysis.

6. Treatment of study subjects

6.1. Study therapy

6.1.1. Treatment regimen and duration

Patients in both arms will receive adalimumab as a SC injections according to the following scheme: 80 mg on Day 1 of Week 0 followed by 40 mg on Day 1 of Week 1, Week 3, Week 5, Week 7, Week 9, Week 11, Week 13, Week 15, Week 17, Week 19, Week 21, Week 23, Week 25, Week 27, Week 29, Week 31, Week 33, Week 35, Week 37, Week 39, Week 41, Week 43, Week 45, Week 47, Week 49, and Week 51.

The patients will be followed up until Week 55.

6.1.1.1. Use of the test drug and reference drug

The patients who met the eligibility criteria will be centrally randomized (with double blinding) into one of the study arms at a 1:1 ratio.

- Patients allocated to Arm 1 (n = 172) will receive BCD-057 (JSC BIOCAD) at a dose of 80 mg on Day 1 of Week 0 followed by a dose of 40 mg on Day 1 of weeks 1, 3, 5, 7, 9, 11, 13, 15, 17, 19, 21, and 23.
- Patients allocated to Arm 2 (n = 172) will receive Humira® at a dose of 80 mg on Day 1 of Week 0 followed by a dose of 40 mg on Day 1 of weeks 1, 3, 5, 7, 9, 11, 13, 15, 17, 19, 21, and 23.

To evaluate the effects of switching from the originator to the biosimilar, patients will be re-randomized at Week 24. As a result of this second randomization, patients from the Humira® arm will be assigned at a 1:1 frequency to one of the two sub-arms.

- Patients in sub-Arm 1 will stay on Humira® 40 mg and receive injections on Day 1 of weeks 25, 27, 29, 31, 33, 35, 37, 39, 41, 43, 45, 47, 49, and 51.
- Patients in sub-Arm 2 will receive injections of BCD-057 40 mg on Day 1 of weeks 25, 27, 29, 31, 33, 35, 37, 39, 41, 43, 45, 47, 49, and 51.

To maintain the double-blind study design, patients from the BCD-057 arm will also be invited for re-randomization. However, in this case, this will be just a nominal procedure where patients will be assigned new randomization IDs and lot numbers. They will continue BCD-057 according to the following regimen: 40 mg on Day 1 of weeks 25, 27, 29, 31, 33, 35, 37, 39, 41, 43, 45, 47, 49, and 51.

Regardless of the treatment arm to which they are assigned, patients will be followed up until Week 55 (if the patient has not been earlier removed from the study).

Patients who drop out due to reasons other than AEs/SAEs (not safety-related) can be replaced before Week 16 at the discretion of the Sponsor.

Injections of BCD-057/Humira® will be given at the study sites by a responsible qualified member of the study team (nurse, doctor, etc.).

6.1.1.1.1. Packaging and labeling of the investigational products

In this study, each patient will use two individual study kits for the whole therapy cycle.

Each study kit contains 14 syringes (one kit for each study period) and has its own unique lot number labeled on the primary and secondary packaging.

All syringes will be packed in blisters. The study drug and the reference drug will have the same primary and secondary packaging. They will be identified only by the lot number. The syringes will not differ by color.

6.1.2. Preparation of the investigational products for administration, and administration procedure

6.1.2.1. Test drug and reference drug

Injections of BCD-057/Humira® will be given at the study sites by a responsible qualified member of the study team (nurse, doctor, etc.).

- Take the syringe from its packaging. Do not shake the syringe.
- Choose an injection site. In can be the abdomen or the anterior aspects of the thigh. Rotate injection sites to different areas for subsequent injections. Do not inject the drug into areas where the skin is swollen, hard or painful.
- Disinfect the skin at the planned injection site.
- Hold carefully the skin at the injection site and slightly pull it up.
- Remove the cap from the needle. With one quick, short motion, push the needle into the skin at a 90° angle.
- Inject the drug slowly by pushing the plunger for 2 to 5 seconds until the syringe is empty.
- Remove the needle from the skin.
- Press a cotton ball at the injection site.
- Do not massage or rub the injection site.
- There may be a small amount of blood at the injection site.

6.1.3. Adjustment and discontinuation of study therapy

6.1.3.1. Dose modification and discontinuation for the test or reference drug

No dose modifications are allowed either for BCD-057 or Humira®. The patients should not compensate for missed doses of BCD-057/Humira® (e.g. the patient should not double the next dose of BCD-057/Humira® if he/she have missed the previous one).

During the treatment with adalimumab, the following events have been reported: infections (including tuberculosis and reactivation of hepatitis B), neurological disorders in patients with demyelinating diseases of the CNS, malignancies, allergies, hematological complications (including pancytopenia and aplastic anemia), lupus-like syndrome, and allergic reactions. Adalimumab should be discontinued if the patient develops a serious infection or sepsis, tuberculosis, a severe fungal infection, symptoms suggesting a demyelinating disease, a malignancy, serious allergic reactions, significant blood abnormalities, severe CHF and/or symptoms of lupus-like syndrome.

The investigator decides whether to discontinue the therapy (temporarily or permanently) taking into account the patient’s complaints and general health state, physical exam results, laboratory values, and other factors indicating that continuation of the study therapy is not possible, dangerous to the patient or does not benefit the patient’s well-being and safety.

6.1.3.2. Acceptable changes in visit schedule

All patients

In the case of adverse events - for not more than 14 days. If the AE does not resolve in 14 days and, in the investigator’s opinion, poses the risk to patient’s health, the patient has to be withdrawn from the study. However, if the investigator and the CRA believe that the patient who discontinued adalimumab for more than 14 days may get benefit from the therapy, **adalimumab treatment can be continued**.

Patients not involved in the PK study

Due to any reasons not related to treatment - for not more than 7 days (this window can be extended if justified by the investigator and approved by the Sponsor).

Patients involved in the PK study

Any deviations from the visit schedule are strongly discouraged. If necessary, the visit can be postponed for 3 days within the following periods: From Visit 1 (Week 0) to Visit 8 (Week 13) and from Visit 11 (Week 17) to Visit 14 (Week 23).

Deviations from the visit schedule starting from Visit 9 (Week 15) and to Visit 10-3 (Day 6 of Week 16) are allowed only due to AEs/SAEs.

Starting from Visit 15 (Week 24), visits can be postponed for not more than 7 days due to any reasons not related to therapy. This period can be extended if the investigator provides an appropriate justification and the Sponsor approved re-scheduling.

If the second or subsequent doses of BCD-057 are postponed, the dates of the next visits will be postponed to account for the actual date of the postponed dose, according to the study schedule. If the Protocol stipulates no injection on the postponed visit, the subsequent visits are performed according to the original schedule.

6.1.3.3. Follow-up for toxicity

In case of temporary or permanent therapy discontinuation, the patient should be followed up until the resolution or stabilization of the event. If the therapy is suspended for more than 14 days, it should be discontinued permanently. However, if the investigator and the CRA believe that the patient who discontinued adalimumab for more than 14 days may get benefit from the therapy, **adalimumab treatment can be continued**.

To detect AEs and SAEs, all patients will be followed up for 28 days from the last dose of BCD-057/Humira®.

6.1.4. Overdose

6.1.4.1. Overdose with the test drug and/or the reference drug

The maximum tolerable dose of adalimumab in humans has not been established. Multiple doses of up to 10 mg/kg did not produce any toxicity that would require dose reduction. No specific antidote is available to adalimumab.

In case of an overdose, the subject should be monitored for any toxicity signs. If any toxicity signs are observed, standard symptomatic therapy should be administered.

6.2. Concomitant therapy, medications allowed and prohibited by the Protocol

6.2.1. Allowed concomitant therapy

Study subjects should tell the study team about any new medications that they have received after the initiation of the study therapy. All medications (except for BCD-057/Humira®) and significant non-medication therapies (including physical therapy, herbal medicines/agents of natural origin, and blood transfusion) used during the study had to be documented in the CRF (sections *Concomitant Therapy* and/or *Surgery or Other Medical Procedures*).

Study subjects may take any medications that are not prohibited by the Protocol.

Patients are allowed to use topical moisturizers, emollients, oils, salicylic acid products, topical antibacterial and antimycotic agents, and topical glucocorticoids as needed. All patients must discontinue all topical skin products (medications or cosmetics) 24 hours before the planned PASI assessment.

If the patient has been using any allowed concomitant therapy, he/she has to continue it at the same dose and regimen throughout the entire study (if reasonable from the medical point of view). On days when PK blood samples are taken (in a limited population), the patient has to continue the usual regimen of his/her concomitant therapy. However, if the patient uses occasionally any concomitant medication during the study, he/she will be requested to avoid it on the days of blood sampling, if possible.

6.2.2. Prohibited concomitant therapy

Treatments prohibited in this study:

- Phototherapy (including selective phototherapy (UVB) and PUVA).
- Systemic[33] glucocorticoids (oral or parenteral glucocorticoids are prohibited regardless of the indication).
- Genetically engineered biological medicinal products (tumor necrosis factor alpha blockers, anti-cytokine agents, etc.).
- Systemic non-biological anti-inflammatory agents (methotrexate, sulfasalazine, chlorambucil, leflunomide, cyclosporine A, azathioprine, cyclophosphamide, cyclosporine, apremilast, mycophenolate mofetil, etc.).
- Aromatic retinoids (acitretin).
- Live or attenuated vaccines.

If, in the investigator’s opinion, the patient needs any of the medications listed above, the patient has to be withdrawn from the study before the initiation of prohibited therapy.

6.3. Compliance

The patient is considered non-compliant if he/she misses more than 4 injections and/or visits during the study (compliance < 85%) or misses 2 consecutive injections and/or visits or breaches the specified time windows for more than 5 visits. Patients are not allowed to miss the first blood sampling.

---

[33] The Protocol allows single doses of systemic glucocorticoids to manage acute anaphylactic reactions, shock or other life-threatening events that developed as adverse events in patients during the study.

7. Efficacy evaluation

7.1. List of efficacy variables

7.1.1. Definition of variables

The efficacy will be assessed based on the indicators of the disease activity and patient's general health.

The PASI is a commonly used tool to assess the area and severity of psoriasis in clinical trials. The PASI score is an integral value from 0 (no symptoms) to 72 (the most severe psoriatic process). It shows the area affected by psoriasis (on the head, legs, arms, and trunk) and includes the severity of erythema, scaling, and induration. There are several modifications of PASI scoring, but the most conventional one is a scale considering all the three signs given above. The information value of the method decreases is the body surface area affected by psoriasis is too small.

The PGA scale is another common tool used to assess the severity of psoriasis. The scale includes 5, 6, or 7 items that the investigator uses to assess the severity of the disease. In most versions of the PGA scale, the morphology of plaques and the area of skin affected by psoriasis are not taken into account. This makes the assessment rather intuitive. The PGA score 0 refers to no signs of psoriasis, while the maximum score refers to the highest disease severity. This scale has several modifications, and the sPGA (static PGA) is the most commonly used one. The sPGA scale allows assessing psoriasis signs at a certain time point. The dPGA (dynamic PGA) scale can be also used in clinical studies. However, when using the dPGA, the physician should remember how he/she assessed the severity of psoriasis during the previous assessment, and this is not always possible. This study uses the sPGA scale consisting of 5 questions.

The body surface area (BSA) affected by psoriasis is another conventional endpoint used in clinical studies. This measure allows an objective assessment of the extent of psoriasis.

This study also evaluates the health-related quality of life. The SF-36 is a standard validated and commonly used questionnaire to assess this parameter. The DLQI questionnaire allows specifically assessing the quality of life in patients with skin diseases.

7.1.2. Efficacy endpoints

Primary endpoint:

- The proportion of patients with a 75% or greater PASI improvement (reduction) from baseline (PASI 75) at Week 16.

Secondary endpoints:

- Changes from baseline in PASI (%) at weeks 16, 24, 33, and 55, by study arms.
- The proportion of patients with a 75% reduction from baseline in psoriasis area and severity (PASI 75 response) at weeks 24, 33, and 55.
- The proportion of patients with a 50% and 90% PASI reduction from baseline at weeks 16. 24, 33, and 55.
- The proportion of patients with sPGA score reduced to 0 (complete clearance) or 1 (minimal disease) at weeks 16, 24, 33, and 55.
- Changes from baseline in affected BSA (%) at weeks 16, 24, 33, and 55.
- Changes from baseline in NAPSI (%) at weeks 16, 24, 33, and 55.
- Change from baseline in the itch severity (VAS score from 0 to 100) at weeks 16, 24, 33, and 55.
- Changes from baseline in health-related quality of life (DLQI and SF-36 scores) at weeks 16, 24, 33, and 55.

7.1.3. Justification of the efficacy endpoints

The primary efficacy endpoint in this study is the proportion of patients who achieved a PASI 75 response at Week 16. This is a sensitive endpoint to assess the efficacy of BCD-057/Humira® in patients with moderate-to-severe plaque psoriasis. This measure is widely used in clinical studies of medicinal products for the treatment of psoriasis and is recommended by the EMA's *Guideline on Clinical Investigation of Medicinal Products Indicated for the Treatment of Psoriasis* (EMEA/CHMP/EWP/2454/02). The time of the primary endpoint evaluation was chosen based on the PK of adalimumab (time to steady state) and is appropriate to evaluate the drug effects according to the EMA's guideline. The use of PASI alone may be not enough for the comprehensive efficacy assessment, so the clinical course of psoriasis in the study will be assessed in detail by analyzing the changes in the BSA, changes in the sPGA score, changes in the absolute PASI scores, and other indicators. This agrees with the EMA's *Guideline on Clinical Investigation of Medicinal Products Indicated for the Treatment of Psoriasis* (EMEA/CHMP/EWP/2454/02).

The follow-up of patients does not stop after the data necessary for the primary endpoint analysis are obtained. The above said variables are assessed throughout the entire study (for about 1 calendar year). The duration of this study allows for assessing the long-term efficacy measures. It agrees with the recommendations provided in the EMA's *Guideline on Clinical Investigation of Medicinal Products Indicated for the Treatment of Psoriasis* (EMEA/CHMP/EWP/2454/02).

7.2. Methods and timeframes for assessment, documenting, and analysis of efficacy variables

7.2.1 Timeframes for analysis of efficacy variables

The analysis of the primary efficacy endpoint will be performed at Week 16 of the blinded therapy with BCD-057/Humira®.

The secondary endpoints (at weeks 24, 33, and 55) will be analyzed when these results are obtained.

7.2.2 Methods and timeframes for assessment and documenting of efficacy variables

To assess whether the study has achieved its objective and to evaluate the treatment efficacy, the analysis will be performed in in two populations: the ITT (intent-to-treat) population (all randomized patients) and the PP (per protocol) population (includes subjects who have no major protocol deviations up to and including Week 16).

The treatment efficacy will be assessed with the PASI, sPGA, BSA, and the health-related QoL scores (DLQI and SF-36).

8. Safety evaluation

8.1. List of safety variables

8.1.1. Terms and Definitions

8.1.1.1. Adverse events

An Adverse Event (AE) is any untoward medical occurrence in a patient or clinical investigation subject administered a pharmaceutical product and which does not necessarily have to have a causal relationship with this treatment.

An adverse event (AE) can therefore be any unfavorable and unintended sign (including an abnormal laboratory finding), symptom, or disease temporally associated with the use of a medicinal product, whether or not related to the medicinal product: from treatment initiation through 28 days after patient's withdrawal from the clinical study.

8.1.1.2. Serious adverse events

A Serious Adverse Event (SAE) is any untoward medical occurrence that at any dose:

- Results in death
- Is life-threatening
- Requires hospitalization or its prolongation
- Results in persistent or significant incapacitation or disability
- Is a congenital anomaly or birth defect.

In case of any uncertainty whether the event meets the seriousness criteria or not, it should be treated as as a serious adverse event.

An immediate risk of death from a reported event is considered life-threatening. A life-threatening event does not include an adverse event that, had it occurred in a more severe form, might have caused death, but is not associated with an immediate risk of death in the form it occurred. For example, hepatitis resolved without signs of hepatic insufficiency will not be considered life-threatening in spite of the fact that more severe hepatitis can lead to fatal outcome. Similarly, an allergic reaction resulting in face angioedema will not be considered life-threatening, although larynx angioedema, allergic bronchospasm or anaphylaxis can lead to fatal outcome.

Hospitalization is an official admission to the hospital. Hospitalization or its prolongation is a criteria of AE seriousness; however, hospitalization itself is not a serious adverse event. Hospitalization or its prolongation not associated with an AE should not be reported by the investigator as a serious adverse event. This is applicable (including but not limited) to the following cases:

- Hospitalization or prolongation of existing hospitalization is necessary to perform procedures required by the Protocol.
- Hospitalization or prolongation of existing hospitalization is a part of routine procedures in this study site (for example, stent removal after a surgery). An appropriate confirmation should be kept in a study file.
- Hospitalization due to a pre-existing condition which did not aggravate.

Disability is a significant impairment of the patient's ability to live normal life.

Other reportable information. Certain information not considered an SAE must be recorded, presented in the report, and followed up as an SAE. This includes:

The use of the investigational product during pregnancy. If the pregnancy is confirmed, the study therapy should be discontinued immediately.

- The use of the investigational product during breastfeeding, regardless of AE development.
- An overdose with the study drug as defined in the Protocol, whether or not it was associated with an AE.
- Unintentional or accidental use of the investigational product, whether or not considered related to an AE.
- Medication errors whether or not considered related to an AE (including confusion or possible confusion during the administration of investigational products).
- Death whether or not associated with an AE.

8.1.1.3. Unexpected adverse events

An unexpected adverse reaction is an adverse reaction, the nature or severity of which is not consistent with the applicable product information (e.g., Investigator's Brochure for an unapproved investigational medicinal product).

8.1.2. Safety endpoints

- The proportion of patients who developed AEs/SAEs that, in the investigator’s opinion, are related to Humira® or BCD-057, by study arms.
- The proportion of patients who experienced grade 3/4 AEs (CTCAE v. 4.03) that, in the investigator’s opinion, are related to Humira® or BCD-057.
- The proportion of patients who developed AEs potentially due to TNFα inhibitors, by study arms. Such AEs include infections, serious infections, opportunistic infections (infections caused by opportunistic pathogenic viruses or cellular organisms), tuberculosis, malignancies, demyelinating diseases, lupus-like syndrome, congestive heart failure, allergic reactions, injection site reactions, and CBC or blood biochemistry abnormalities.

- The proportion of patients who discontinued the study due to AEs/SAEs, by study arms.

8.2. Methods and timeframes for assessment, documenting and analysis of safety variables

8.2.1. Timeframes for analysis of safety variables

Safety endpoints will be analyzed throughout the entire study.

8.2.2. Methods and timeframes for assessment and documenting of safety variables

The safety population will include all randomized patients (ITT population). In addition, the study report will contain data on serious adverse events reported during screening (if any).

The safety evaluation is based on the frequency and pattern of AEs/SAEs, laboratory abnormalities, and vital sign abnormalities in the therapy arms. Patients will undergo regular physical examination (including blood pressure, pulse, and body temperature checks), CBC and biochemistry tests, urinalysis, ECG, and chest X-ray and/or fluorography.

The frequency of these procedures is shown in Table 12, section 4.6.1 *Study Visits and Procedures*. AEs/SAEs will be documented and reported in accordance with the Sponsor's Manual.

The investigator is responsible for recording adverse events in the clinical study.

AEs must be captured starting from the first dose of the investigational product and through 4 weeks after the last dose. AEs related to the protocol procedures must be recorded from the moment the subject signed the ICF. SAEs must be captured from the moment the patient signed the ICF and through 4 weeks after the last dose of the investigational product. The study investigator can report SAEs that occurred after the required follow-up if he/she considers them related to the investigational product or study procedures.

The study investigator must document non-serious AEs in the source documents and in the CRF, and show them to the CRA at the next monitoring visit. All serious adverse events (SAEs) must be reported to the Sponsor immediately **(within 24 hours**). Reports must be sent to the Sponsor immediately (within 24 hours) by fax (495) 992 82 98 (ext. 116), addressed to PV Officer, JSC BIOCAD, or by e-mail safety@biocad.ru (with "To PV Officer" in the subject field). The investigator should receive a receipt/delivery confirmation. Information regarding unexpected adverse reactions must be presented to the CRA at the next scheduled visit.

8.3. Requirements for reports, procedures for registration and reporting AEs, and filling out AE Report Forms

8.3.1. Documenting and Reporting AEs/SAEs

At every visit, a record must be made whether or not any AEs occurred during the period from the previous visit. Any adverse event reported for a patient since he/she signed the ICF must be recorded in source documents and in an Adverse Event Report Form (in the CRF). AEs will be documented in accordance with the Sponsor's Manual.

AEs should be registered and numbered consecutively as they occur. Each AE is reported in an *AE Report Form*. Rules for reporting AE/SAE are described in details in the Sponsor's Manual.

Adverse events must be recorded regardless of their seriousness and causal relationship with the study therapy. If an adverse event re-emerges, it must be recorded as a new AE and assigned a new number.

Laboratory and vital sign abnormalities

Laboratory and/or vital sign abnormalities considered to be adverse events (assessed as clinically meaningful, including those of CTCAE 4.03 grade 1, inducing clinical symptoms or complaints requiring concomitant therapy or changes in study therapy) must be recorded in the CRF, *Adverse Events* section. All adverse events of CTCAE 4.03 grade 2 or higher must be registered regardless of their clinical significance. It is preferable to specify a diagnosis rather than individual symptoms (for example, anemia instead of decreased hemoglobin). Laboratory abnormalities meeting the criteria for an adverse event should be followed up until they return to normal or until an adequate explanation is obtained.

8.3.2. AE/SAE reporting

The AE Report Form should be filled out during the study visit (or the investigator can do it later the same day), except for data not available/not known at that moment. All sections of the form must be filled out. If certain data are not available, the following should be entered: "Data not available" or "N/A". However, all actions should be taken to receive all necessary data on emerging adverse events.

The following information is recorded in source documents and the AE Report Form of the CRF:

- AE No. as it occurred
- Visit No.
- A brief description of the AE

- Seriousness (yes/no)
- Seriousness criterion
- CTCAE 4.03 grade
- Onset date
- Date of resolution (return to normal or baseline)
- Outcome
- Actions taken due to the AE
- Causal relationship with the drug (test drug or reference drug)
- Comments specifying any clinically relevant (in the investigator's opinion) information related to AE development or therapy.

If a medication therapy is administered, its components should be described in section "Concomitant therapy" of the CRF, with a notice that a medication was used to manage the AE.

Columns *Grade*, *Outcome*, *Measures*, and *Causal Relationship* should be filled using digital codes explained in the *Notes* section. If the investigator considers an AE to be an SAE, he/she must fill out an SAE Report Form in addition to an AE Report From (printed copies).

One SAE Report Form is filled out for one SAE. If the AE remains unresolved at the next visit, the checkbox in the *Adverse Events* section of the eCRF should be ticked for this visit and marked as "unresolved". In this case, a new SAE Report Form should be filled out marked as "Follow-up".

The SAE Report Form must contain the following information:

1. Study information:
   - Protocol ID
2. SAE information:
   - SAE name
   - Initial or Follow-up
   - Internal SAE code assigned by the Sponsor (this field is filled out by the Sponsor)
3. Information on the investigator who reported the SAE, and information on the study site:
   - Full name of the investigator who reported the SAE
   - Contact information (tel. and e-mail)
   - Site name and code
   - Principal Investigator's name
4. Information on the study subject:
   - Patient ID
   - Sex
   - Body weight

- Height
- Date of birth
- Renal/hepatic impairment
- Pregnancy (yes / no)
- Allergies

5. Information on the study drug:
    - INN and trade name (if this is a blind study, names are listed with "/")
    - Indication
    - Date when the investigational product was first administered
    - Number of therapy cycles received (if applicable)
    - Drug batch (after which the SAE developed)
    - Dose (with which the SAE occurred)
    - Treatment start date and time (followed by the SAE)
    - Treatment stop date and time (followed by the SAE)
    - Route of administration
    - Dosage and dosing frequency
    - Is the therapy code disclosed or not (for blind studies)
6. Information about recent (within one month) concomitant therapy or that used at the time of SAE onset;
    - INNs and brand names of concomitant medications
    - Indication
    - Treatment start date
    - Treatment stop date
    - Dosages, frequency and route of administration
    - Any suspected causal relationship between the SAE and concomitant medications

7. SAE narrative:
    - SAE description with all symptoms and laboratory/instrumental abnormalities and time frames indicated.
    - Time after the last dose of the investigational product
    - Autopsy data if the subject died (specifying the cause of death according to postmortem conclusion). If no autopsy findings are available, the cause of death should be specified according to the clinical conclusion.

- Time of hospitalization (if applicable)

8. Severity (CTCAE 4.03)
9. Seriousness
10. Medical history (with dates)
11. Investigations of particular interest at the onset of the SAE
    - Investigation
    - Normal limits
    - Date of the analysis
    - Result
12. Actions taken to resolve the SAE
    - Medication / non-medication
    - For medication therapy the following should be specified: INNs and brand names of drugs, treatment start and stop dates, dosages, frequencies and routes of administration.
13. Actions taken regarding the investigational product
    - Discontinued/ dose reduce / unchanged
    - Dechallenge/rechallenge test results (if applicable)
14. Outcome
15. Signatures

The investigator **must** sign (put a signature and a full name) each page of the SAE Report Form to verify his/her responsibility for the reported information. Signed SAE Report Form is then submitted to the Sponsor.

8.4. Methods and duration of follow-up for study subjects after the onset of AE/SAE

If the patient is withdrawn from the study due to an AE/SAE, the investigator should perform further patient's treatment and follow-up as per standard institutional practices for the treatment of the certain AE or SAE. The patient should be followed up until **the AE/SAE resolves completely**. In case of any laboratory, instrumental or vital sign abnormality, the patient should be followed up until the abnormality resolves completely (in other words, until the abnormality resolves or improves to the baseline level).

See section 5.4 for further details.

The patient withdrawn from the study due to pregnancy that occurred during the study should be monitored through the entire pregnancy and for 6 months after delivery to evaluate the mother's and child's health. Information on pregnancy course and outcome should be recorded in the source documentation. During the entire period of pregnancy, the investigator in collaboration with

the attending Ob/Gyn specialist should monitor the patient's overall health, the course of pregnancy, and laboratory values including ultrasound. When the child is born, the investigator together with the attending pediatrician should monitor the newborn for 6 months evaluating the child's clinical status and the laboratory/instrumental findings.

If the study subject's partner becomes pregnant, the study subject should provide the contacts of attending Ob/Gyn specialist to the investigator. The investigator should monitor woman's health over the entire course of pregnancy. Monitoring should be performed via phone calls to attending Ob/Gyn specialist once every 3 months to analyze the overall woman's health, pregnancy course, and laboratory and instrumental findings including the ultrasound findings. When the child is born, the investigator together with the attending pediatrician should monitor the newborn for 6 months evaluating the child's clinical status and the laboratory/instrumental findings.

8.5. Immunogenicity study

Immunogenicity of BCD-057/Humira® will be assessed using the proportions of patients who develop antibodies to adalimumab (binding and/or neutralizing).

The immunogenicity analysis will include all patients who received at least one dose of BCD-057/Humira®. Patients whose serum samples taken on Day 1 of Week 0 and at least one serum sample taken on any subsequent visit (weeks 16, 33, and 55) are missing/lost/spoiled, will be removed from immunogenicity analysis.

Immunogenicity of BCD-057/Humira will be assessed by evaluating titers of binding and neutralizing anti-adalimumab antibodies (BAbs and NAbs).

9. Statistics

9.1. Description of statistical methods

Quantitative data

The quantitative data in this study include the following:

Efficacy:

- PASI
- BSA
- sPGA
- NAPSI
- Itch severity (VAS, 0 mm to 100 mm).
- Health-related quality of life (DLQI and SF-36)

Safety:

CBC results

- Biochemistry results
- Urinalysis results
- Vital signs (BP, pulse, body temperature)

Pharmacokinetics:

- Adalimumab concentration in the serum

The statistical analysis will be performed using two-tailed hypothesis tests. The statistical significance level is 0.05.

The Shapiro-Wilk test will be used to test the qualitative data for normality.

Normally distributed data will be compared using the following tests: the two-tailed Student's *t*-test, Welch's *t*-test, and ANOVA.

Non-normally distributed data will be compared using the following tests: the Mann-Whitney test, Wilcoxon test, Kruskal-Wallis test, and Friedman's test.

To describe the normally distributed quantitative variables, the following parameters will be used: means, SDs, min, max, and coefficient of variation. For non-normally distributed quantitative data, it is planned to use median and quartiles, minimum, maximum, and coefficient of variance.

The statistical hypothesis of the equivalence by the primary endpoint will be tested by comparing limits of the 95% CI with the equivalence margin.

The null hypothesis (H_0: $|\varepsilon| \geq \delta$, where δ is the equivalence margin) is rejected, and the drugs are considered equivalent if the bounds of the 95% CI for the 95% CI for the difference in the mean proportions of PASI 75 responders fall within the equivalence margin (δ = 0.15).

Categorical data

The categorical data in this study include the following:

Efficacy:

- The proportion of patients with a 75% PASI reduction from baseline.
- The proportion of patients with 50% and 90% PASI reduction from baseline.
- The proportion of patients with sPGA score reduced to 0 (complete clearance) or 1 (minimal disease).

Safety:

- ECG findings.
- Urinalysis results.
- The proportion of patients who experienced AEs that, in the investigator's opinion, are related to Humira® or BCD-057.
- The proportion of patients who experienced grade 3/4 AEs (CTCAE v. 4.03) that, in the investigator's opinion, are related to Humira® or BCD-057.
- The proportion of patients in each study arm who experienced AEs potentially due to TNFα inhibitors. These AEs include infections, serious infections, opportunistic infections (infections caused by opportunistic pathogenic viruses or cellular organisms), tuberculosis, malignancies, demyelinating diseases, lupus-like syndrome, congestive heart failure, allergic reactions, injection site reactions, and CBC or blood biochemistry abnormalities.
- The proportion of patients who discontinued the study due to AEs/SAEs, by study arms.

Immunogenicity:

- The proportion of patients with binding or neutralizing anti-adalimumab antibodies.
- The proportion of patients with binding or neutralizing anti-adalimumab antibodies and inadequate therapeutic response (achieved PASI 50 or less).

The categorical data will be processed using frequency tables, contingency tables, exact Fisher's test, test for equality of frequencies, χ^2 test, and Cochran-Mantel-Haenszel test. The categorical data will be described using percentages or proportions.

Correction for multiple comparisons will be performed with the Benjamini-Yekutieli procedure.

Statistical methods will be chosen based on the type and distribution of raw data. Appropriate statistical methods will be determined when all the data are obtained, since data distribution and sample homogeneity cannot be assessed in advance. A list of methods may be expanded if necessary for conducting a high-quality study.

9.2. Statistical analysis steps and timelines for reports

Efficacy analysis using the primary endpoint

The final report will present analysis of the efficacy and safety endpoints after the 16-week observation of 344 included patients.

Supplementary report No. 1

First supplementary report will present analytical results for PK, efficacy, safety, and immunogenicity using data obtained after 33 weeks.

Supplementary report No. 2

Second supplementary report will present analytical results for efficacy, safety, and immunogenicity using data obtained from the entire study (55 weeks).

Supplementary report

The supplementary report will present analytical results for the secondary efficacy, safety, PK, and immunogenicity endpoints assessed using data obtained over the entire study (55 weeks).

9.3. Planned number of subjects. Justification of sample size, including reasoning or calculations to justify statistical power, and clinical justification of the study

Efficacy assessment

The primary endpoint in this study is the efficacy measure, namely the proportion of PASI 75 responders by study arms.

The recent EMA's recommendations (*Guideline on Similar Biological Medicinal Products Containing Biotechnology-Derived Proteins as Active Substance: Non-Clinical and Clinical Issues* [EMEA/CHMP/BMWP/42832/2005 Rev1, 2014] and *Guideline on Similar Biological Medicinal Products Containing Monoclonal Antibodies – Non-Clinical and Clinical Issues* [EMA/CHMP/BMWP/403543/2010, 2012]) state that, in general, clinical studies of biosimilars should use an equivalence design. While the study was being planned, the hypothesis of the test product being equivalent to the reference product ($H_0 : |\varepsilon| \geq \delta$, $H_1 : |\varepsilon| < \delta$, where ε is the true

differences in the mean values of the efficacy endpoint between the arms, and δ is the equivalence margin of the test and reference products) was tested using the following error values: type I error is 5% (α=05), type II error: 20% (β=2); the power of test is 80%.

[redacted]

[redacted]

[redacted]

[redacted]

[redacted]

[redacted]

[redacted]

[redacted]

[redacted]

[redacted]

[redacted]

9.4. Suitable significance level

The level of significance is set as 0.05 (5%); the power of the test is set as 0.8 (80%).

9.5. Statistical criteria for stopping and/or terminating the study

Not specified by the Protocol.

9.6. Handling of missing, unevaluable or uncertain data

All information specified in the eCRF has to be confirmed by appropriate data in the source documents.

After entering all data in the electronic database, an employee keeping the database checks it for inconsistencies, errors, and missing data points. To collect missing data or correct wrong data, the BIOCAD's Data Manager and Medical Expert generate queries, which are patient- and site-specific, i.e. generated for each subject individually. The CRA sends queries to the study site by fax or e-mail. The investigator must respond within 5 working days from the date when the query was received. Copies of responses to queries must be kept at the study site; original responses must be stored at JSC BIOCAD.

When responses to queries are received from investigators, the employee keeping the database checks them for inconsistencies, errors, and missing data points When all the data from all the sites are collected and entered, the database is locked, and the statistical processing can be performed.

Missing, unevaluable or uncertain data are not subject to replacement.

Uncertain or unevaluable data will be detected during the outlier analysis by examination of Mahalanobis or Cook distance, visual analysis of scattering diagrams and box-plots. Data suspected to be outliers will be processed by the biomedical statistician together with the medical expert and, if necessary, the principal investigator.

9.7. Reporting any deviations from the initial statistical plan

If the initial study plan requires modifications, all changes will be described and explained in a protocol amendment or interim/final clinical study report.

If initially defined statistical methods cannot be used, the changes should be explained in the final statistical report and the clinical study report. Justification of these changes should be given with the references to calculations, statistical parameters, and analysis of a situation that led to these changes. Decisions regarding emergency deviations (data-modifying allowances) can be made only by the Sponsor. These decisions must be explained and justified, including in the final study report.

9.8. Selection of subjects for analysis

Efficacy analysis

To assess whether the study has achieved its objective and to evaluate the treatment efficacy, the analysis will be performed in two populations: the ITT (intent-to-treat) population (all randomized patients) and the PP (per protocol) population (includes subjects who have no major protocol deviations up to and including Week 16).

Safety analysis

The safety population will include all randomized patients (ITT population). In addition, the study report will contain data on serious adverse events reported during screening (if any).

Immunogenicity analysis

The immunogenicity analysis will include all patients who received at least one dose of BCD-057/Humira®. Patients whose serum samples taken on Day 1 of Week 0 and at least one serum sample taken on any subsequent visit (weeks 16, 33, and 55) are missing/lost/spoiled, will be removed from the immunogenicity analysis.

PK analysis

The PK study will involve a limited population (90 patients from each study arm, 180 subjects total). The PK analysis will include all of these 180 patients who:

- Completed all visits for PK assessment starting from Day 1 of Week 15 to Day 6 of Week 16, except for those who missed more than two PK blood samplings during this period.
- Provided a blood specimen for adalimumab assay on Day 1 of Week 0 (before the first injection of BCD-057/Humira®);
- Missed not more than one dose of adalimumab within the period from the study start to Week 15 inclusive (injections 1 to 9).

10. Direct access to source data/documents

The investigator/institution involved in the study must ensure direct access to source data/documents by the monitor, the auditor, the LEC/IRB or regulatory authorities.

11. Quality control and quality assurance

11.1. Data quality assurance

According to the ICH GCP and regulatory requirements, the Sponsor, a third party on its behalf, regulatory authorities, or local ethics committees can perform audits (inspections) to assure quality any time during the study or after its completion. The investigator must give the auditor/inspector direct access to all study documents (including source documents) and discuss (personally, or designate someone from the study team) the audit/inspection results and other matters with the auditor/inspector.

11.2. Investigator's adherence to the Protocol

Before beginning the study, the investigator must read and accept all provisions of this Protocol. The investigator must conduct the study in accordance with this Protocol, the ICH GCP, and other regulatory requirements of participating countries.

No protocol deviations are allowed during the study without a previous written approval from JSC BIOCAD, the Ministry of Healthcare of the Russian Federation and Local Ethics Committees, except for the cases when the deviation is necessary to protect a patient from an immediate hazard.

The investigator should have enough time to accurately perform and complete the study within the timeframes specified by JSC BIOCAD, enough employees of appropriate qualification, and adequate equipment to conduct the study according to the Protocol.

Each sub-investigator participating in the study must read the Protocol and be aware of his/her responsibilities/functions in the study. If the principal investigator delegates some of his/her functions to sub-investigators, this must be documented in a relevant section of the Investigator's File.

11.3. Investigator's responsibility to comply with the Protocol

At each study site, the decision regarding patient's early withdrawal from the study has to be approved by JSC BIOCAD.

If the investigator decides to withdraw a patient from the study, he/she must send a request, specifying a reason for withdrawal, to BIOCAD's Medical Advisory Division by fax: +7 (495) 992-82-98. Within 48 hours (except for weekends and public holidays) from the time when the request was received by the Medical Advisory Department, JSC BIOCAD should notify the investigator of the decision regarding patient withdrawal. If a patient has to be immediately withdrawn from the study due to an SAE, the investigator must inform JSC BIOCAD about the SAE within 24 hours but does not have to wait for approval from JSC BIOCAD.

If a subject does not attend a scheduled visit or makes unauthorized changes in the dose of the investigational product, the investigator must report the violation to the Medical Advisory Division

of JSC BIOCAD within 24 hours from the time of awareness (by fax: + 7 (495) 992-82-98). Medical advisors will instruct the investigator on further case management and on how to document the causes of the violations in the CRF/source documents.

If the investigator fails to follow these procedures or if multiple Protocol violations occur, JSC BIOCAD may suspend or terminate the study at this particular study site.

11.4. Study monitoring

Before the study beginning (at the Study Initiation Visit or investigators' meeting), a representative (CRA) of JSC BIOCAD (or an authorized CRO) will explain the Protocol and the CRF to the investigators and members of the study teams. During the study, the CRA will regularly visit the study site to check the completeness of patient records, accuracy of information in the CRF, adherence to the Protocol and GCP, process of patients' recruitment, and storage, dispensing, and accounting of the investigational products according to applicable requirements. During these visits, key members of the study team should be available to assist the CRA and resolve arising issues (if any).

Study monitoring is performed according to appropriate SOPs of JSC BIOCAD.

For each study subject, the investigator should keep source documents containing the subject data and records made during visits (medical records of the study center), including demographics, medical information, laboratory findings, ECG, and all other tests or examinations. Any information contained in the CRF should be also recorded in the patient's source documents. The investigator must keep the original ICF. A copy of the signed ICF will be given to the patient.

The investigator should provide the CRA with all relevant patient source documents to confirm that data in the source documents is consistent with the data in the CRF. The investigator should ensure the timely completeness of the CRFs before the CRA's visit.

The CRA will check the CRFs and other study materials comparing them against the source data to confirm that the study complies with the Declaration of Helsinki, the ICH GCP, regulatory requirements of participating countries, and the Study Protocol, and to confirm the authenticity, accuracy, and completeness of data.

Upon the study completion, a representative of JSC BIOCAD (CRA) should visit the study site to perform the study closeout visit. During this visit, the Sponsor's representative will collect all necessary documentation in accordance with the SOP of JSC BIOCAD.

11.5. Data management and quality control

In the trials that involve eCRFs, JSC BIOCAD employees (or employees of an authorized CRO) will check the data entered by the study team members for accuracy and completeness. If there are

any inconsistent or missing data points, queries will be generated with a request for clarification. All queries are sent to the study site. A designated member of the study team must immediately answer the request and make all required changes to the database.

At the end of the study, any protocol deviations will be determined. After clarifying protocol deviations and confirming the completeness and accuracy of the data, the database is locked, the blind codes are opened, and the data are ready for analysis.

11.6. Study termination

JSC BIOCAD can suspend or terminate the study due to safety or ethical issues, Protocol compliance issues, or due other reasons. If JSC BIOCAD suspends or terminates the study, the study site will be notified in advance. In case of suspension or termination, JSC BIOCAD and the investigator have to inform Ethics Committees and regulatory authorities in due time. If the study is suspended or terminated, all study information must be transferred to and all unused investigational product must be returned to JSC BIOCAD.

12. Ethics

12.1. Ethical aspects of the study

The study will be conducted in compliance with the ethical principles stated in the World Medical Association Declaration of Helsinki (*Recommendations Guiding Physicians in Biomedical Research Involving Human Subjects*, 1964-1996) and the ICH GCP principles.

Before the initiation of the study, the final version of the Protocol (including the Patient Information Sheet and Informed Consent Form) will be submitted for approval to the Ministry of Healthcare of the Russian Federation and the Local Ethics Committees.

All subsequent protocol amendments (other than administrative amendments) must be approved before implementation.

Informed consent must be obtained from patients before starting any study procedures. The Patient Information Sheet contains all the information that a patient may need to make a conscious and independent decision about whether to participate.

During the study, all cases of SAEs will be reported to JSC BIOCAD within 24 hours. JSC BIOCAD will analyze the reports and may suspend the study if considered necessary. Local Ethics Committees will also be notified of all SAEs that are related, in the investigator’s opinion, to the investigational product.

All patient personal information is confidential and can be disclosed only if required by law (including court decisions).

All study subjects will be insured. If a subject gets injured directly due to the investigational product, the Sponsor will cover all reasonably justified treatment expenses.

12.2. Confidentiality of study subjects

The investigator shall protect confidentiality of the trial subjects, the text of this Protocol, and all other study materials and results.

The investigator must ensure subject anonymity. In the CRFs and other documents provided by JSC BIOCAD, patients should be identified by ID codes and/or initials, but never by their names.

The investigator should keep a separate log with Subject IDs, last names, addresses, phone numbers, and medical record numbers (if applicable). The investigator must keep confidentiality of the data not intended for submission to JSC BIOCAD.

All study materials proprietary to JSC BIOCAD cannot be transferred to a third party unless required by the law of the Russian Federation.

13. Data handling and record keeping

13.1. Record keeping at the study site

All study documents must be archived at the study site or at the central archive of the institution. A list of all study subject identifiers should be made.

According to the ICH GCP, essential documents include: signed protocol and amendments; copies of completed CRFs; signed ICFs for all patients; medical records; diaries and other source documents; approvals from LECs and regulatory authorities and all correspondence including approved documents; drug accountability records; study correspondence; and the list of patient names and addresses. These are the essential documents that must be kept in the Investigator's File.

The investigator must retain copies of all essential documents for 5 years.

By the end of this period, the Sponsor will inform the investigator(s) about the date when the documents may be destroyed.

Study subject documentation will be archived in accordance with the site in-house SOPs.

The investigator must inform the Sponsor about the place where essential documents are stored and request an approval from JSC BIOCAD before destroying any of essential documents. Appropriate measures must be taken to prevent accidental or premature destruction of these documents.

13.2. Confidentiality of data

All information about study subjects will be kept confidential. The information will be processed in compliance with all applicable laws and regulations. These regulations require informing study subjects and obtaining their written authorization regarding the following: questions:

- What protected health information will be collected in this study?
- Who will have access to this information and on what grounds?
- Who will use or disclose this information?
- Do study subjects have the right to recall their consent for using their confidential health information?

According to the current regulations, if the patient recalls his/her authorization to collect or use his/her protected health information, the investigator still can use all information obtained before the authorization was withdrawn. If the patient recalls authorization to collect or use his/her protected health information, the investigator should do as much as possible to get patient's permission for collecting at least the safety information (i.e. onset of new or aggravation of existing adverse events) until the scheduled end of the study period.

To prevent unauthorized access to protected subject information, the data management system uses integrated safety elements encrypting all the data when sending them in both directions. The access to the system will be controlled with a sequence of individually assigned identification codes and user passwords. These codes and passwords will be given only to authorized members of the study team who have received a special training.

13.3. Collection of data

This trial uses an electronic data capture (EDC) system. Designated study team members will enter the data required by the Protocol to eCRFs. The eCRFs have been developed with a validated and safe web-based software. The study team members will get access to the EDC system only after being appropriately trained. An automated validation program inspects the eCRFs for inconsistencies and allows the study team members to change or verify the entered data.

The principal investigator is responsible for completeness and accuracy of all the data entered to the eCRFs and for entering and updating the information in a timely manner.

Members of the study team will collect blood samples for the assessments of PK, immunogenicity, and safety of the investigational products. The samples will be then sent to the Central Laboratory for processing.

14. Finance and insurance

[redacted]

[redacted]

[redacted]

[redacted]

[redacted]

[redacted]

15. Publications

After completion of the study, its results will be summarized and prepared for publication. The investigator must not publish any study results, including those obtain at his/her study site, without a permission from JSC BIOCAD. Results from individual study sites must not be published before the publication of the overall study results.

16. Appendices

Appendix 1. Dermatology Life Quality Index, DLQI

1	Over the last week, how itchy, sore, painful or stinging has your skin been?	Very much ☐<br>A lot ☐<br>A little ☐<br>Not at all ☐	
2	Over the last week, how embarrassed or self-conscious have you been because of your skin?	Very much ☐<br>A lot ☐<br>A little ☐<br>Not at all ☐	
3	Over the last week, how much has your skin interfered with you going shopping or looking after your home or garden?	Very much ☐<br>A lot ☐<br>A little ☐<br>Not at all ☐	Not applicable ☐
4	Over the last week, how much has your skin influenced the clothes you wear?	Very much ☐<br>A lot ☐<br>A little ☐<br>Not at all ☐	Not applicable ☐
5	Over the last week, how much has your skin affected any social or leisure activities?	Very much ☐<br>A lot ☐<br>A little ☐<br>Not at all ☐	Not applicable ☐
6	Over the last week, how much has your skin made it difficult for you to do any sport?	Very much ☐<br>A lot ☐<br>A little ☐<br>Not at all ☐	Not applicable ☐
7	Over the last week, has your skin prevented you from working or studying?	Yes ☐<br>No ☐	Not applicable ☐
	If “No”, over the last week how much has your skin been a problem at work or studying?	A lot ☐<br>A little ☐<br>Not at all ☐	
8	Over the last week, how much has your skin created problems with your partner or any of your close friends or relatives?	Very much ☐<br>A lot ☐<br>A little ☐<br>Not at all ☐	Not applicable ☐
9	Over the last week, how much has your skin caused any sexual difficulties?	Very much ☐<br>A lot ☐<br>A little ☐<br>Not at all ☐	Not applicable ☐
10	Over the last week, how much of a problem has the treatment for your skin been, for example by making your home messy, or by taking up time?	Very much ☐<br>A lot ☐<br>A little ☐<br>Not at all ☐	Not applicable ☐

Appendix 2. SF-36 questionnaire

**Instructions: Note:** This survey contains questions to find out how do you feel about your health. This information will help us to monitor your health and how easily you perform your usual activities. Please answer each question by marking your response as shown below. If you are not sure how to answer, please choose one option that reflects your opinion the best. Please encircle only one digit in each line.

1. In general, would you say your health as:

Excellent	Very good	Good	Fair	Poor
1	2	3	4	5

2. Compared to one year ago, how would you rate your health in general now?

Much better now than one year ago	Somewhat better now than one year ago	About the same	Somewhat worse now than one year ago	Much worse now than one year ago
1	2	3	4	5

3. The following items are about activities you might do during a typical day. Does your health now limit you in these activities? If so, how much?

	Yes, limited a lot	Yes, limited a little	No, not limited at all
a. Vigorous activities, such as running, lifting heavy objects, participating in strenuous sports	1	2	3
b. Moderate activities, such as moving a table, pushing a vacuum cleaner, picking berries and mushrooms [bowling, or playing golf]	1	2	3
c: Lifting or carrying groceries	1	2	3
d. Climbing several flights of stairs	1	2	3
e. Climbing one flight of stairs	1	2	3
f. Bending, kneeling, or stooping	1	2	3
g. Walking more than a kilometer	1	2	3
h. Walking several blocks	1	2	3
i. Walking one block	1	2	3
j. Bathing or dressing yourself	1	2	3

4. During the past 4 weeks, have you had any of the following problems with your work or other regular daily activities as a result of your physical health?

	Yes	No
a. Cut down the amount of time you spent on work or other activities	1	2
b. Accomplished less than you would like	1	2
c. Were limited in the kind of work or other activities	1	2
d. Had difficulty performing the work or other activities (for example, it took extra effort)	1	2

5. During the past 4 weeks, have you had any of the following problems with your work or other regular daily activities as a result of any emotional problems (such as feeling depressed or anxious)?

	Yes	No
a. Cut down the amount of time you spent on work or other activities	1	2
b. Accomplished less than you would like	1	2
c. Didn't do work or other activities as carefully as usual	1	2

SF-36 QUESTIONNAIRE (continued)

6. During the past 4 weeks, how much did your physical health or emotional problems interfere with your normal social activities with family, friends, neighbors, or groups?

Not at all	Slightly	Moderately	Severe	Very severe

1	2	3	4	5

7. How much bodily pain have you had during the past 4 weeks?

None	Very mild	Mild	Moderate	Severe	Very severe
1	2	3	4	5	6

8. During the past 4 weeks, how much did pain interfere with your normal work (including both work outside the home and housework)? Please give one answer.

Not at all	A little bit	Moderately	Quite a bit	Extremely
1	2	3	4	5

9. These questions are about how you feel and how things have been with you during the past 4 weeks. For each question, please give the answer that comes closest to the way you have been feeling. How much of the time during the past 4 weeks...

	All of the time	Most of the time	A good bit of the time	Some of the time	A little of the time	None of the time
a. Did you feel full of pep?	1	2	3	4	5	6
b. Have you been a very nervous person?	1	2	3	4	5	6
c. Have you felt so down in the dumps that nothing could cheer you up?	1	2	3	4	5	6
d. Have you felt calm and peaceful?	1	2	3	4	5	6
e. Did you have a lot of energy?	1	2	3	4	5	6
f. Have you felt downhearted and blue?	1	2	3	4	5	6
g. Did you feel worn out?	1	2	3	4	5	6
h. Have you been a happy person?	1	2	3	4	5	6
i. Did you feel tired?	1	2	3	4	5	6

10. During the past 4 weeks, how much of the time has your physical health or emotional problems interfered with your social activities (like visiting with friends, relatives, etc.)? Please give one answer.

All of the time	Most of the time	Some of the time	A little bit of the time	None of the time
1	2	3	4	5

11. How TRUE or FALSE is each of the following

statements for you?	Definitely true	Mostly true	Don't know	Mostly false	Definitely false
A. I seem to get sick a little easier than other people	1	2	3	4	5
B. I am as healthy as anybody I know	1	2	3	4	5
C. I expect my health to get worse	1	2	3	4	5
D. My health is excellent	1	2	3	4	5

Appendix 3. Static Physicians Global Assessment (sPGA)

Score	Category	Description
0	Clear	Plaque elevation = 0 (no elevation over normal skin)<br>Scaling = 0 (no scale)<br>Erythema = 0 (residual post-inflammatory hyper- or hypopigmentation)
1	Minimal	Plaque elevation = +/- (possible but difficult to ascertain whether there is a slight elevation above normal skin)<br>Scaling = +/- (surface dryness with some white coloration)<br>Erythema = up to moderate (up to definite red coloration)
2	Mild	Plaque elevation = slight (slight but definite elevation, typically edges are indistinct or sloped)<br>Scaling = fine (fine scale partially or mostly covering lesions)<br>Erythema = up to moderate (up to definite red coloration)
3	Moderate	Plaque elevation = moderate (moderate elevation with rough or sloped edges)<br>Scaling = coarser (coarse scale covering most of all of the lesions)<br>Erythema = moderate (definite red coloration)
4	Severe	Plaque elevation = marked (marked elevation typically with hard or sharp edges)<br>Scaling = coarse (coarse, non-tenacious scale predominates covering most or all of the lesions)<br>Erythema = severe (very bright red coloration)
5	Very severe	Plaque elevation = very marked (very marked elevation typically with hard sharp edges)<br>Scaling = very coarse (coarse, thick tenacious scale over most of all of the lesions; rough surface)<br>Erythema = very severe (extreme red coloration; dusky to deep red coloration)

Appendix 4. Injection Site Reaction Form

INJECTION SITE REACTION FORM

Visit No: __ __

Product Lot: ___________ Dose ______ Injection date ___/___/201__

Brief description:

Severity* 1☐; 2☐; 3☐; 4☐.

First signs appeared _______ after injection

Clinically meaningful? Yes / No Does it meet the SAE criteria?** Yes / No

Description	Revealed	Size cm	Onset date	End date
Hyperemia	Yes / No		___/___/201__	___/___/201__
Edema	Yes / No		___/___/201__	___/___/201__
Blister	Yes / No		___/___/201__	___/___/201__
Infiltration	Yes / No		___/___/201__	___/___/201__
Necrosis	Yes / No		___/___/201__	___/___/201__
Ulcer	Yes / No		___/___/201__	___/___/201__
Cyanosis/bruise	Yes / No		___/___/201__	___/___/201__
Pain	Yes / No	Score____***	___/___/201__	___/___/201__
Other: _______________	Yes / No		___/___/201__	___/___/201__

Had a consultation with a surgeon/dermatologist Yes/No

Results of a consultation:

Performed medication and non-medication treatment, outcome:

* classification of severity grades of injection site reactions

1. Mild: transient symptoms or mild discomfort lasting for less than 2 days and not requiring treatment or any other intervention.
2. Moderate: mild or moderate limitation of everyday activity; assistance may be required; medication therapy is minimum or not required.
3. Severe: significant limitation of everyday activity; assistance is required; medication therapy is required; hospitalization may be required.
4. Life-threatening: severe limitation of everyday activity; significant assistance is required; medication therapy is required; hospitalization is required.

**In the case of an SAE, fill out the SAE Report Form in the CRF

*** Subjective pain assessment with VAS. Where 0 refers to no pain and 100 refers to the most severe, insupportable, intolerable pain, as judged by the patient.
