# Supplementary material for: Long-term data on the proposed adalimumab biosimilar BCD-057 in patients with moderate to severe psoriasis: A randomized controlled trial
Source: PLoS One. 2022 Feb 7;17(2):e0263214. doi: 10.1371/journal.pone.0263214 (PMC8820628; doi:10.1371/journal.pone.0263214)
Supplement: S2 File — (PDF) [file pone.0263214.s003.pdf]

S3 Table. Study Centers and Ethics Committees.

**The Russian Federation**
**Study site #1**<br>Federal State Budgetary Healthcare Institution Siberian Regional Medical Center of the Federal Medical and Biological Agency<br>Local Ethics Committee of Federal State Budgetary Healthcare Institution Siberian Regional Medical Center of the Federal Medical and Biological Agency<br>Submission number: 9/2016<br>Date of approval: 19 December 2016
**Study site #2**<br>Federal State Budgetary Educational Institution of Higher Professional Education Tver' State Medical University of the Ministry of Healthcare of the Russian Federation<br>Ethics Committee of Federal State Budgetary Educational Institution of Higher Professional Education Tver' State Medical University of the Ministry of Healthcare of the Russian Federation<br>Submission number: NA (not assigned as not required by local regulations)<br>Date of approval: 26 December 2016
**Study site #3**<br>Regional State Budgetary Healthcare Institution Smolensk Dermatology and Venereology Clinic<br>Multiregional Independent Ethics Committee at Institute for Clinical Pharmacology<br>Submission number: 63<br>Date of approval: 07 March 2017
**Study site #6**<br>State Budgetary Educational Institution of Higher Professional Education N.I. Pirogov Russian National Research Medical University of the Ministry of Healthcare of the Russian Federation<br>Local Ethics Committee of Federal State Budgetary Educational Institution of Higher Education N.I. Pirogov Russian National Research Medical University of the Ministry of Healthcare of the Russian Federation<br>Submission number: 160<br>Date of approval: 19 December 2016
**Study site #7**<br>State Budgetary Institution of Sverdlovsk Region Ural Research Institute of Dermatovenereology and Immunopathology<br>Ethics Committee of State Budgetary Institution of Sverdlovsk Region Ural Research Institute for Dermatovenereology, and Immunopathology<br>Submission number: 1<br>Date of approval: 10 January 2017
**Study site #10**<br>Limited Liability Company Sanavita<br>Independent Committee for Ethical Review of Clinical Trials Pharmexpert<br>Submission number: 1<br>Date of approval: 23 February 2017
**Study site #11**<br>State Autonomous Healthcare Institution Republican Clinical Dermatology and Venereology Clinic

Local Ethics Committee of State Autonomous Healthcare Institution Republican Clinical Dermatology and Venereology Clinic<br>Submission number: 40<br>Date of approval: 27 December 2016
**Study site #13**<br>Federal State Budgetary Military Educational Institution of Higher Education S.M. Kirov Military Medical Academy of the Ministry of Defense of the Russian Federation<br>Independent Ethics Committee at Federal State Budgetary Military Educational Institution of Higher Education S.M. Kirov Military Medical Academy of the Ministry of Defense of the Russian Federation<br>Submission number: 183<br>Date of approval: 20 December 2016
**Study site #14**<br>State Budgetary Institution of Ryazan Region Regional Clinical Dermatology and Venereology Clinic<br>Ethics Committee at State Budgetary Institution of Ryazan Region Regional Clinical Dermatology and Venereology Clinic<br>Submission number: 9<br>Date of approval: 15 December 2016
**Study site #15**<br>St. Petersburg State Budgetary Healthcare Institution Dermatology and Venereology Center No. 10 - Dermatology and Venereology Clinic<br>Independent Ethics Committee at St. Petersburg State Budgetary Healthcare Institution Dermatology and Venereology Center No. 10 - Dermatology and Venereology Clinic<br>Submission number: 30<br>Date of approval: 02 May 2017
**Study site #16**<br>State Healthcare Institution Regional Dermatology and Venereology Clinic<br>Local Ethics Committee at State Healthcare Institution Regional Dermatology and Venereology Clinic<br>Submission number: 6<br>Date of approval: 23 December 2016
**Study site #17**<br>State Budgetary Educational Institution of Higher Education Kazan State Medical University of the Ministry of Health of the Russian Federation<br>Local Ethics Committee at Federal State Budgetary Educational Institution of Higher Education Kazan State Medical University of the Ministry of Healthcare of the Russian Federation<br>Submission number: 10<br>Date of approval: 20 December 2016
**Study site #18**<br>State Budgetary Healthcare Institution Clinical Dermatology and Venereology Clinic of the Ministry of Healthcare of Krasnodar Region<br>Local Ethics Committee at State Budgetary Healthcare Institution Clinical Dermatology and Venereology Clinic of the Ministry of Healthcare of Krasnodar Region<br>Submission number: 65<br>Date of approval: 14 December 2016
**Study site #19**

State Budgetary Clinical Healthcare Institution of Yaroslavl Region N.A. Semashko City Hospital<br>Ethics Committee at State Budgetary Clinical Healthcare Institution of Yaroslavl Region N.A. Semashko City Hospital<br>Submission number: 13<br>Date of approval: 29 December 2016
**Study site #20**<br>Limited Liability Company Novaya Klinika<br>Local Ethics Committee at State Budgetary Healthcare Institution of Stavropol Region Pyatigorsk Multiregional Cancer Clinic<br>Submission number: 28<br>Date of approval: 16 December 2016
**Study site #22**<br>Pierre Wolkenstein Skin Diseases Clinic Limited Liability Company<br>Independent Ethics Committee at Limited Liability Company Pierre Wolkenstein Skin Diseases Clinic<br>Submission number: 13/2016<br>Date of approval: 15 December 2016
**Study site #23**<br>Federal State Budgetary Educational Institution of Higher Education V.I. Razumovskiy Saratov State Medical University of the Ministry of Healthcare of the Russian Federation<br>Local Ethics Committee at Federal State Budgetary Educational Institution of Higher Education V.I. Razumovskiy Saratov State Medical University of the Ministry of Healthcare of the Russian Federation<br>Submission number: 4<br>Date of approval: 06 December 2016
**Study site #25**<br>Limited Liability Company Medical and Sanitary Clinic No. 157<br>Independent Ethics Committee at Limited Liability Company Clinical Research Partner<br>Submission number: 01/2017<br>Date of approval: 13 January 2017
**Study site #26**<br>Limited Liability Company Baltiyiskaya Meditsina<br>Independent Ethics Committee at Limited Liability Company Clinical Research Partner<br>Submission number: 01/2017<br>Date of approval: 13 January 2017
**Study site #27**<br>State Budgetary Healthcare Institution Chelyabinsk Regional Clinical Dermatology and Venereology Clinic<br>Local Ethics Committee at State Budgetary Healthcare Institution Chelyabinsk Regional Clinical Dermatology and Venereology Clinic<br>Submission number: 6<br>Date of approval: 03 March 2017
**Study site #30**<br>Federal State Budgetary Research Institution V.A. Nasonova Research Institute for Rheumatology

Ethics Committee at Federal State Budgetary Research Institution V.A. Nasonova Research Institute for Rheumatology<br>Submission number: 06<br>Date of approval: 30 March 2017
**Study site #31**<br>Limited Liability Company Ekologiya Zdoroviya<br>Local Ethics Committee at Limited Liability Company Ekologiya Zdoroviya<br>Submission number: 3<br>Date of approval: 14 February 2017
**Study site #35**<br>State Budgetary Healthcare Institution of Nizhniy Novgorod Region N.A. Semashko Nizhniy Novgorod Regional Clinical Hospital<br>Ethics Committee at State Budgetary Healthcare Institution of Nizhniy Novgorod Region N.A. Semashko Nizhniy Novgorod Regional Clinical Hospital<br>Submission number: 1<br>Date of approval: 26 January 2017
**Study site #41**<br>Federal State Budgetary Educational Institution of Higher Education V.F. Voyno-Yasenetskiy Krasnoyarsk State Medical University of the Ministry of Healthcare of the Russian Federation<br>Local Ethics Committee at Federal State Budgetary Educational Institution of Higher Education V.F. Voyno-Yasenetskiy Krasnoyarsk State Medical University of the Ministry of Healthcare of the Russian Federation<br>Submission number: 74/2016<br>Date of approval: 03 February 2017
**Study site #42**<br>Private Medical Institution Evromedservis<br>Independent Ethics Committee at Limited Liability Company Clinical Research Partner<br>Submission number: 01/2017<br>Date of approval: 13 January 2017
**Study site #43**<br>Limited Liability Company Technologiya Zdoroviya<br>Independent Ethics Committee at Limited Liability Company Clinical Research Partner<br>Submission number: 01/2017<br>Date of approval: 13 January 2017
**The Republic of Belarus**
**Study site #50**<br>Health Institution Vitebsk Regional Clinical Center for Dermatology, Venereology and Cosmetology<br>Local Ethics Committee at Health Institution Vitebsk Regional Clinical Center for Dermatology, Venereology and Cosmetology<br>Submission number: 5<br>Date of approval: 28 April 2017
**Study site #51**<br>Healthcare Institution Mogilev Regional Dermatology and Venereology Clinic<br>Ethics Committee at Healthcare Institution Mogilev Regional Dermatology and Venereology Clinic<br>Submission number: 6

Date of approval: 02 May 2017
**Study site #52**<br>Healthcare Institution City Clinical Dermatology and Venereology Clinic<br>Ethics Committee at Healthcare Institution City Clinical Dermatology and Venereology Clinic<br>Submission number: 5<br>Date of approval: 21 June 2017
